# Genomic Surveillance of SARS-CoV-2 in the Southern Province of Zambia: Detection and Characterization of Alpha, Beta, Delta, and Omicron Variants of Concern

**DOI:** 10.3390/v14091865

**Published:** 2022-08-24

**Authors:** Ben Katowa, Annie Kalonda, Benjamin Mubemba, Japhet Matoba, Doreen Mainza Shempela, Jay Sikalima, Boniface Kabungo, Katendi Changula, Simbarashe Chitanga, Mpanga Kasonde, Otridah Kapona, Nathan Kapata, Kunda Musonda, Mwaka Monze, John Tembo, Matthew Bates, Alimuddin Zumla, Catherine G. Sutcliffe, Masahiro Kajihara, Junya Yamagishi, Ayato Takada, Hirofumi Sawa, Roma Chilengi, Victor Mukonka, Walter Muleya, Edgar Simulundu

**Affiliations:** 1 Macha Research Trust, Choma 20100, Zambia; 2 Department of Biomedical Sciences, School of Veterinary Medicine, University of Zambia, Lusaka 10101, Zambia; 3 Department of Biomedical Sciences, School of Health Sciences, University of Zambia, Lusaka 10101, Zambia; 4 Department of Disease Control, School of Veterinary Medicine, University of Zambia, Lusaka 10101, Zambia; 5 Africa Centre of Excellence for Infectious Diseases of Humans and Animals, School of Veterinary Medicine, University of Zambia, Lusaka 10101, Zambia; 6 Department of Wildlife Sciences, School of Natural Resources, Copperbelt University, Kitwe 50100, Zambia; 7 Department of Biomedical Sciences, School of Medicine, Copperbelt University, Ndola 50100, Zambia; 8 Churches Health Association of Zambia, Lusaka 10101, Zambia; 9 Southern Provincial Health Office, Ministry of Health, Choma 20100, Zambia; 10 Department of Paraclinical Studies, School of Veterinary Medicine, University of Zambia, Lusaka 10101, Zambia; 11 Department of Preclinical Studies, School of Veterinary Medicine, University of Namibia, Windhoek Private Bag 13301, Namibia; 12 School of Life Sciences, College of Agriculture, Engineering and Sciences, University of KwaZulu-Natal, Private Bag X54001, Durban 4000, South Africa; 13 Zambia National Public Health Institute, Ministry of Health, Lusaka 10101, Zambia; 14 Virology Laboratory, University Teaching Hospital, Lusaka 10101, Zambia; 15 HerpeZ Infection Research and Training, University Teaching Hospital, Lusaka 10101, Zambia; 16 School of Life and Environmental Sciences, University of Lincoln, Lincoln, Lincolnshire LN6 7TS, UK; 17 Division of Infection and Immunity, Centre for Clinical Microbiology, University College London, NIHR Biomedical Research Centre, University College London Hospitals NHS Foundation Trust, London NW3 2PF, UK; 18 Department of International Health, Johns Hopkins Bloomberg School of Public Health, Baltimore, MD 21205, USA; 19 Department of Epidemiology, Johns Hopkins Bloomberg School of Public Health, Baltimore, MD 21205, USA; 20 Division of Global Epidemiology, International Institute for Zoonosis Control, Hokkaido University, N20 W10, Kita-ku, Sapporo 001-0020, Japan; 21 Division of Collaboration and Education, International Institute for Zoonosis Control, Hokkaido University, N20 W10, Kita-ku, Sapporo 001-0020, Japan; 22 International Collaboration Unit, International Institute for Zoonosis Control, Hokkaido University, N20 W10, Kita-ku, Sapporo 001-0020, Japan; 23 One Health Research Center, Hokkaido University, N18 W9, Kita-ku, Sapporo 001-0020, Japan; 24 Division of Molecular Pathobiology, International Institute for Zoonosis Control, Hokkaido University, N20 W10, Kita-ku, Sapporo 001-0020, Japan; 25 Division of International Research Promotion, Hokkaido University International Institute for Zoonosis Control, N20 W10, Kita-ku, Sapporo 001-0020, Japan; 26 Global Virus Network, 725 W Lombard Street, Baltimore, MD 21201, USA; 27 Republic of Zambia State House, Lusaka 10101, Zambia

**Keywords:** SARS-CoV-2, COVID-19, variants of concern, spike mutations, whole-genome sequencing, Zambia

## Abstract

Severe acute respiratory syndrome coronavirus 2 (SARS-CoV-2) variants of concern (VOCs) have significantly impacted the global epidemiology of the pandemic. From December 2020 to April 2022, we conducted genomic surveillance of SARS-CoV-2 in the Southern Province of Zambia, a region that shares international borders with Botswana, Namibia, and Zimbabwe and is a major tourist destination. Genetic analysis of 40 SARS-CoV-2 whole genomes revealed the circulation of Alpha (B.1.1.7), Beta (B.1.351), Delta (AY.116), and multiple Omicron subvariants with the BA.1 subvariant being predominant. Whereas Beta, Delta, and Omicron variants were associated with the second, third, and fourth pandemic waves, respectively, the Alpha variant was not associated with any wave in the country. Phylogenetic analysis showed evidence of local transmission and possible multiple introductions of SARS-CoV-2 VOCs in Zambia from different European and African countries. Across the 40 genomes analysed, a total of 292 mutations were observed, including 182 missense mutations, 66 synonymous mutations, 23 deletions, 9 insertions, 1 stop codon, and 11 mutations in the non-coding region. This study stresses the need for the continued monitoring of SARS-CoV-2 circulation in Zambia, particularly in strategically positioned regions such as the Southern Province which could be at increased risk of introduction of novel VOCs.

## 1. Introduction

As of 6 July 2022, the ongoing coronavirus disease 2019 (COVID-19) pandemic has caused over 548,990,094 confirmed cases including 6,341,637 deaths [1]. In Africa, despite having a total population of about 1.3 billion, the official reports show a low burden of severe acute respiratory syndrome coronavirus 2 (SARS-CoV-2) infections when compared with other continents. The total number of confirmed cases and fatalities reported in Africa were 9,138,803 and 173,674, respectively, representing a global burden of 1.7% [1]. However, post-mortem and serological studies in some African countries suggest that the true burden of SARS-CoV-2 infections and deaths may be higher than what is officially reported [2,3,4,5]. Further, a recent systematic review by the World Health Organisation (WHO) on the seroprevalence of SARS-CoV-2 in Africa revealed that over two-thirds of the African population had been infected by SARS-CoV-2 [6]. The analysis further revealed that the true number of SARS-CoV-2 infections on the African continent was 97 times higher than the reported confirmed cases and the sharp rise in incidence was attributed to the introduction of the highly transmissible Alpha and Delta variants [6,7]. 

The first COVID-19 case in Africa was reported in Egypt on 14 February 2020 [8,9] followed by Algeria, with its first case being reported on 25 February 2020 [10] and Nigeria on 27 February 2020 [11]. Most African countries including Cameroon, Morocco, Senegal, South Africa, Togo, and Tunisia reported their first cases by mid-March 2020 [8,12] and most of the index cases were imported cases from Europe which by then had become the epicentre of the pandemic [8,12]. Within three months of Africa’s COVID-19 index case, 54 of 55 African Union (AU) Member States (except Western Sahara) had reported over 100,000 cases which included imported and community transmissions [8]. The early phase of the pandemic in Africa was characterized by the predominance of lineage B.1 which was introduced multiple times in African countries [13]. However, due to a ban on international air travel in most African countries and the world at large in March/April 2020, the number of SARS-CoV-2 importations into Africa decreased and the pandemic entered a phase that was characterized by sustained low levels of within-country spread and occasional international viral dissemination between neighbouring countries, presumably via road and rail links between these countries [13].

As the pandemic progressed, several SARS-CoV-2 variants carrying mutations with concerning phenotypic implications on current pandemic management strategies emerged [14]. Of particular significance to the ongoing pandemic are SARS-CoV-2 variants designated variants of concern (VOCs). Several VOCs have been described including Alpha (B.1.1.7), Beta (B.1.351), Gamma (P.1), Delta (B.1.617.2), and Omicron (B.1.1.529). VOCs are associated with enhanced transmissibility or virulence, reduction in neutralization by antibodies obtained through natural infection or vaccination, the ability to evade detection, or a decrease in therapeutic or vaccination effectiveness [15,16]. Further, all the five reported VOCs have mutations in the receptor-binding domain (RBD) and the N-terminal domain (NTD), of which N501Y mutation located on the RBD is common to all variants except the Delta variant [16]. The N501Y mutation results in increased binding affinity of the spike (S) protein to angiotensin-converting enzyme (ACE) 2 receptors thereby enhancing the viral attachment and its subsequent entry into the host cells [17,18]. Other genomic changes have been reported, including the extensive deletion in the open reading frame (ORF) 7a, ORF8 [19,20,21], and a deletion in the nsp2 genes [22], but these deletions have been associated with mild to moderate clinical symptoms compared to the infection caused by the wildtype SARS-CoV-2 [21,23].

To date, four VOCs, namely Alpha, Beta, Delta, and Omicron have been detected on the African continent. The first VOC, designated Alpha (B.1.1.7), was detected in September 2020 in the United Kingdom (UK) and was introduced into Africa between November 2020 and February 2021 with evidence of local transmission in Nigeria and Ghana [13]. This variant is characterised by nine mutations in the S protein, increased transmissibility, and increased risk of hospitalisation [24,25]. The second VOC was the Beta (B.1.351 lineage) variant which was first detected in South Africa in October 2020 and became the most common variant in many African countries [26]. This VOC is characterized by mutations in the S protein, including in the RBD—K417N, E484K, and N501Y [14,26]. In addition, the Beta variant is known to cause severe disease in young and healthy individuals [26]. Whereas the Beta variant was associated with the second wave of SARS-CoV-2 in Africa, the Alpha variant did not predominate in many African countries possibly due to a lack of selective advantage over the other VOCs [27]. These variants were replaced by the highly transmissible Delta (B.1.617.2 lineage) variant which was initially detected in India in December 2020 and spread worldwide among vaccinated as well as unvaccinated individuals [28]. This variant seeded the third wave of the pandemic in 2021 and was introduced in Africa in June 2021. The Omicron variant, characterised by several mutations in the S protein, including a set of mutations previously observed in other VOCs and novel mutations, was first reported in South Africa on 24 November 2021 and became the dominant driver of the fourth global wave of SARS-CoV-2 [29]. 

In Zambia, the first known COVID-19 cases were reported on 18 March 2020 from travellers returning from Europe [30]. Within days, the government implemented restrictions on international travel, school closures, halting of non-essential business, and confinement of people to their homes. Despite these measures, the virus spread to all parts of the country with over 300,000 cases and over 4000 deaths as of 6 July 2022 [31]. The course of the pandemic in Zambia can be divided into four major waves: the first wave occurred from July to September 2020 and was mainly driven by B.1.1 and its sub-lineages; the second wave occurred from December 2020 to April 2021 and was dominated by the Beta variant, while the Delta variant dominated the third wave from May to September 2021 [32,33]. The Omicron variant has dominated the fourth pandemic wave in Zambia, with cases peaking in early January 2022 and then rapidly decreasing to low levels. In the Southern Province, which shares international borders with Botswana, Namibia, and Zimbabwe and is a major tourist destination, SARS-CoV-2 was first detected in May 2020 [31]. As the course of the pandemic continues to evolve, it remains crucial to monitor and understand the virus evolution and outbreak dynamics, particularly in strategically positioned regions such as the Southern Province which is a trade entry point of Zambia for all imports and exports from Southern Africa. However, there is limited data regarding the molecular epidemiology of SARS-CoV-2 in Zambia, with only two genomic studies reporting the detection of SARS-CoV-2 belonging to lineage B.1.1. [34] and the B.1.351 variant [35]. Moreover, to our best knowledge, no reports have described the genetic characteristics of SARS-CoV-2 VOCs circulating in the Southern Province. Therefore, this study used whole-genome sequencing (WGS) and phylogenetic analyses to describe the genetic characteristics of SARS-CoV-2 in the Southern Province of Zambia.

## 2. Materials and Methods

### 2.1. Study Site and Sample Collection

**Figure 1:**
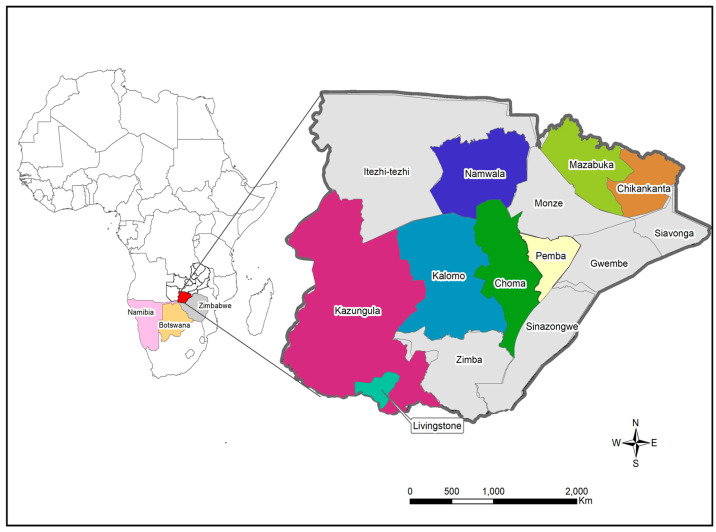
Map showing the location of study sites in Southern Province. The locator map depicts Zambia with neighbouring countries that share the border with Southern Province. The insert map shows the Southern Province of Zambia with the study sites namely Chikankata, Choma, Kalomo, Kazungula, Livingstone, Mazabuka, Namwala and Pemba districts. The maps were generated using Quantum Geographic Information System (QGIS) version 3.10 (http://www.qgis.org (accessed on 8 August 2022).

The study samples were collected between December 2020 and April 2022 from eight districts in the Southern Province (Figure 1). Sample collection was conducted through the Zambia National Public Health Institute under the coordination of the Zambia Genomic Sequencing Consortium. The samples were collected through routine surveillance (i.e., point of entry screening and routine screening for influenza-like illnesses) and targeted surveillance of cluster outbreaks. A total of 198 samples were collected from different parts of the Southern Province and were brought to Macha Research Trust (MRT) for WGS. WGS was conducted in collaboration with the Churches Health Association of Zambia (CHAZ) with 161 samples collected between December 2020 and November 2021 being transported to MRT, while 37 samples collected from December 2021 to April 2022 were transported to CHAZ Complex laboratory for sequencing. Upon receipt, all samples were retested to determine the cycle threshold (Ct) value of each sample. Samples that had a Ct value of ≤30 and were submitted with the relevant metadata were included to undergo WGS. Samples that did not meet the inclusion criteria and those that could not be amplified or had poor genomic coverage were excluded from further analysis.

### 2.2. RNA Extraction and Virus Genome Amplification

Viral RNA was extracted from nasopharyngeal swabs using the QIAamp Viral RNA Mini Kit (Qiagen, Hilden, Germany) or the MagMax kit (Thermo Fisher Scientific, Waltham, MA, USA) on an automated KingFisher Flex platform (Thermo Fisher Scientific, USA) according to manufacturer specifications and protocols. Amplification of the SARS-CoV-2 genome in preparation for WGS was conducted using the Centre for Disease Control and Prevention (CDC) SARS-CoV-2 qRT-PCR assay [36]. 

### 2.3. Next-Generation Sequencing

Whole-genome sequencing was performed using the Oxford Nanopore technologies and Illumina NextSeq platforms. For Oxford Nanopore, a cDNA synthesis reaction was performed on 36 samples (based on cycle threshold values < 30) using SuperScript IV Reverse Transcriptase kit (Invitrogen, Waltham, MA, USA), following the manufacturer’s instructions. Library preparation was conducted using the ARTIC protocol version 3 [37,38]. Whole-genome sequencing was conducted using custom-designed primers (Tables S3 and S4) [34]. The PCR products were cleaned using AMPure XP beads (Beckman Coulter, Brea, CA, USA) and DNA quantification was conducted using a Qubit fluorometer (Thermo Fisher Scientific). End-repair on the amplified samples was conducted using NEBNext Ultra II End Repair Module (New England BioLabs, Ipswich, MA, USA). Native barcode expansion kits 1–12 and 13–24 was used in combination with Ligation Sequencing Kit (SQK-LSK109) (Oxford Nanopore Technologies). Subsequently, genomic sequencing was conducted using the MinION 1MkB (Oxford Nanopore Technologies, Oxford, UK). The RAMPART (v1.0.6) software package was used to monitor sequencing performance in real-time, with runs proceeding until a minimum of approximately 200-fold coverage was achieved across all amplicons. At this point, the run was terminated and the resulting reads were basecalled using Guppy (4.0.14). Consensus sequence generation was conducted using the ARTIC bioinformatics pipeline (https://artic.network/ncov-2019/ncov2019-bioinformatics-sop.html (accessed on 7 October 2021). 

For Illumina NextSeq, V.3 primers pools designed by ARTIC Network were used (https://github.com/joshquick/artic-ncov2019/blob/master/primer_schemes/nCoV-2019/V3/nCoV-2019.tsv (accessed on 7 October 2021)). Sequencing libraries for 37 samples were prepared using the Illumina COVIDSeq kit on the automated Hamilton robotic instrument, ABI 7500 fast, and the Quant Studio thermo-cyclers. After successful library clean-up and pooling, pooled samples were quantified and normalized using a Qubit dsDNA HS Assay kit by diluting from a starting concentration of 4 nM to a final loading concentration of 1 nM. Thereafter, 25 µL was loaded on the Illumina NextSeq 2000 instrument through a cartridge loaded with a flow cell for SARS-CoV-2 genomic sequencing. A customized version of the DRAGEN DNA pipeline was used to perform Kmer-based detection of SARS-CoV-2. The Nextseq 2000 then aligned the reads to a reference genome, calls variants, and generates consensus genome sequences. The NextSeq 2000 optionally performs lineage/clade analysis using Pangolin and NextClade.

### 2.4. Genome Annotation and Phylogenetic Analysis

Whole-genome sequences were annotated using the reference genome of hCoV-19/Wuhan/Hu-1/2019|EPI_ISL_402125 [33]. A dataset of 180 whole genomes was created, which included 40 generated from this study and 140 retrieved from the GISAID database. Audacity *Instant* was used to retrieve SARS-CoV-2 whole-genome sequences from GISAID that were most similar to the sequences generated in this study. We also included reference sequences of VOCs detected in Southern Africa and other parts of the world, targeting those isolated within the same period and belonging to the same lineage as those characterized in this study. Reference sequences with stretches of more than 10% ‘NNNN’ were excluded from the analysis. Multiple sequence alignment of the sequences was performed using the FFT-NS-2 algorithm available in the multiple sequence alignment programme (MAFFT), but otherwise using default settings (https://mafft.cbrc.jp/alignment/server/index.html (accessed on 9 August 2022) [39]. The alignment was inspected in Geneious Prime v2022.0.1 (https://www.geneious.com (accessed on 9 August 2022) and gaps were trimmed. Following alignment, a maximum likelihood (ML) phylogenetic tree was constructed using the PhyML Online server (www.atgc-montpellier.fr/phyml/ (accessed on 9 August 2022) [40] using the smart model selection (SMS) [41] and the Bayesian Information Criterion. Branch support was estimated through the SH-like approximate likelihood ratio test (SH-aLRT). The ML tree was then rooted using TempEst v1.5.3 [42], which estimated the best-fitting root of this phylogeny using the heuristic residual mean squared function, aimed at minimizing the variance of root-to-tip distances. The resultant ML tree file was edited using Interactive Tree of Life (iTOL) v5, an online tool for phylogenetic tree display and annotation [43].

PANGO lineage identification was performed using Pangolin v3.1.16 (https://pangolin.cog-uk.io/ (accessed on 8 August 2022)). Identification of single nucleotide polymorphisms (SNPs) was performed using the coronapp web application http://giorgilab.unibo.it/coronannotator/ (accessed on 8 August 2022). SNPs were identified based on the number of high confidence base calls (consensus sequence variations of the assembly) that do not agree with the reference bases for the genome position of interest. These variations were then exported to a vcf file and visualized in Microsoft Excel. The GISAID accession IDs of the genomes generated in this study can be found in Table S1.

## 3. Results

### 3.1. Characteristics of Patients with COVID-19 from the Southern Province of Zambia

A total of 198 samples were received for WGS from districts in the Southern Province, 74 were negative for SARS-CoV-2, 51 had Ct values > 30, and 33 had a low genome coverage. Only 40 samples were successfully sequenced, 13 samples at MRT and 27 at CHAZ Complex laboratory. 

Demographic data were analyzed for all the 198 samples and the majority of the samples (104/198; 52.5%) were from females as shown in Table 1. The mean age of the participants was 28 (range: 0–82). The data set for gender and age were not available for one and five samples, respectively (Table 1).

### 3.2. SARS-CoV-2 Lineage Assignment and Distribution in Southern Province 

**Figure 2:**
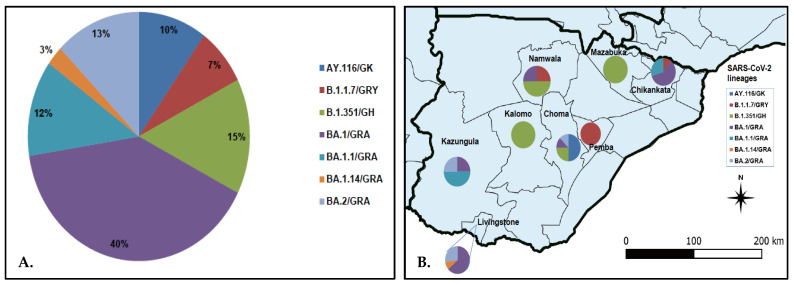
Distribution of SARS-CoV-2 lineage in the Southern Province of Zambia. Panel (**A**) pie chart showing SARS-CoV-2 lineages detected in the Southern Province; panel (**B**) proportionate distribution of SARS-CoV-2 lineages in the eight districts of the Southern Province. The map was generated using Quantum Geographic Information System (QGIS) version 3.10 (http://www.qgis.org (accessed on 8 August 2022)).

SARS-CoV-2 lineage assignment using the PANGOLIN application (https://pangolin.cog-uk.io/ (accessed on 8 August 2022), showed that the 40 genomes detected in this study were distributed into seven lineages, namely AY.116 (Delta), B.1.1.7 (Alpha), B.1.351 (Beta), and Omicron (BA.1, BA.1.1, BA.1.14, and BA.2) (Figure 2A). The largest number of the sequences (*n* = 17, 42.5%) belonged to lineage BA.1/GRA (Figure 2A). All lineage AY.116 sequences came from Choma District, whereas the six B.1.351 lineage was detected in Choma (*n* = 2), Namwala (*n* = 2), Kalomo (*n* = 1), and Mazabuka (*n* = 1) districts (Figure 2B; Table S1). The Alpha variant (B.1.1.7) viruses were found in Namwala, Pemba, and Chikankata districts (Figure 2B; Table S1). Of the 27 Omicron variants, 11 (40.7%) were from Livingstone, 9 (33.3%) from Chikankata, 4 (14.8%) from Kazungula, 2 (7.4%) from Choma and 1 (3.7%) from Namwala. Most lineage BA.1 viruses were detected in Chikankata and Livingstone districts where 7/27 (25.9%) viruses of this lineage were found in each district. Lineage BA.1.1 was detected in Chikankata and Kazungula districts whereas B.1.14 was only detected in Livingstone (Table S1). Three of the five BA.2 lineage viruses were detected in Livingstone whereas the other two were detected in Choma and Kazungula districts as shown in Figure 2B and Table S1. 

### 3.3. Phylogenetic Analysis

**Figure 3:**
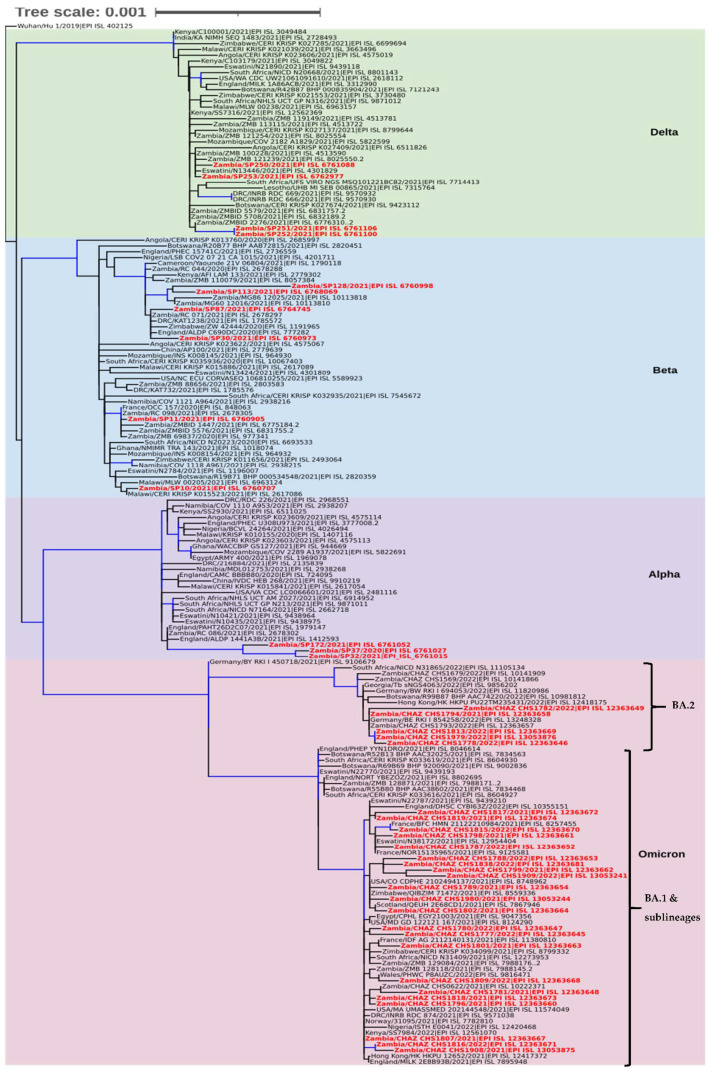
Phylogenetic analysis of SARS-CoV-2 genomes from Zambia and other countries. The genomes generated in this study are indicated in red whereas the shaded areas indicate the clades of variants of concern. Each sequence was named with the country name first followed by the isolate name and then the GISAID accession number. The tree branches highlighted in blue indicate tree branches that had a strong maximum likelihood ratio greater than 0.9, whereas the tree scale represents the nucleotide substitutions per site.

Phylogenetic analysis revealed that the sequences separated into four clades namely Delta, Beta, Alpha, and Omicron (Figure 3). In the Delta clade four Southern Province sequences (Zambia/SP250/2021|EPI ISL 6761088, Zambia/SP253/2021|EPI ISL 6762977, Zambia/SP251/2021|EPI ISL 6761106, and Zambia/SP252/2021|EPI ISL 6761100), separated into two groups of which two formed a distinct cluster with a Zambian isolate whereas the other two clustered with sequences from Angola, Eswatini, and Zambia (Figure 3). Six sequences analysed in this study belonged to the Beta clade and they separated into four distinct clusters. Two of the sequences (Zambia/SP30/2021|EPI ISL 6760973 and Zambia/SP87/2021|EPI ISL 6764745) analysed in this study clustered with isolates from Zambia, Zimbabwe, England, and the Democratic Republic of Congo (DRC), and another set of two formed a distinct cluster with sequences from Zambia. The last two sequences (Zambia/SP11/2021|EPI_ISL_6760905 and Zambia/SP10/2021|EPI_ISL_6760707) belonged to separate clusters with the former sequence grouping with Zambian sequences whereas the latter was closely related to sequences obtained in Malawi, Eswatini, and Botswana (Figure 3). In the Alpha clade, three Southern Province sequences, namely Zambia/SP32/2021|EPI_ISL_6761015, Zambia/SP37/2020|EPI_ISL_6761027, and Zambia/SP172/2021|EPI_ISL_6761052 formed a distinguishable cluster with sequences from England and Zambia (Figure 3). The Omicron clade was separated into two clusters (Figure 3). The majority (22/27; 81.5%) of the Zambian sequences in this clade belonged to the BA.1 sub-lineage cluster whereas the rest (5/27; 18.5%) were of the BA.2 lineage. Phylogenetic analysis further showed that the Omicron sequences from this study were mainly closely related to sequences from European and African countries (Figure 3).

### 3.4. Molecular Analysis

A total of 292 different mutations were detected from the 40 genomes studied when compared to the Wuhan/Hu 1/2019|EPI ISL 402125 reference sequence (Table 2). Most (96.2%) mutations were detected in the coding regions of the genomes. Of the mutations detected in the coding region, 64.8% (182/281) were missense mutations, 23.5% (66/281) were synonymous mutations, 8.2% (23/281) were deletions, 3.2% (9/281) insertions, and one was a stop codon (0.4%), gained with a single nucleotide polymorphism (SNP) on the ORF8 (Table 2 and Table S2). Deletions and insertion included in-frame and out-of-frame mutations. When gene mutations were stratified according to the VOCs, the Alpha variant had a total of 53 different mutations of which 31 (58.5%) were missense mutations and 8 (15.1%) synonymous mutations. The number of mutations in the Alpha variant genomes ranged between 41 and 45 with (EPI_ISL_6761027) having the most mutations. The Beta variant had a total of 68 different mutations with 44 (64.7%) missense mutations and 15 (22.1%) synonymous mutations. The mutations in the Beta variant genomes ranged between 26 and 45 with one sequence (EPI_ISL_6760998) having the most mutations. Further, sequences of the Delta variant had a total of 50 mutations with 37 (74%) missense mutations and 7 (14%) synonymous mutations. The Delta variant mutations ranged between 39 and 44 with two sequences (EPI_ISL_6761106; EPI_ISL_6761100) having the most mutations. Sequences of the Omicron variant had the highest number of mutations; 149 different mutations with 90 (60.4%) missense and 39 (26.2%) synonymous mutations, with the genomes having a mutation range between 48 and 67 with three sequences (EPI_ISL_12363648; EPI_ISL_12363649; EPI_ISL_12363661) having the most mutations. Deletions, insertions, stop-codons, and upstream/downstream gene variants had a frequency below 18% in all the VOCs. 

When the number of mutations per gene was counted only once, the S protein was the most mutated gene with 82 mutations whereas the second mutated gene was the NSP3 protein with 42 mutations (Table S2). Of the 82 mutations in the S protein, 65/82 were missense mutations, 3/82 synonymous mutations, 10/82 deletions, and 4 insertions as shown in Table 2. Among all the SNPs, the most common change was C > T followed by A > G and G > A. Further, a large deletion of 26 nucleotides was observed on position 29734 of the 3′UTR of the four sequences (EPI_ISL_12363646, EPI_ISL_12363658, EPI_ISL_12363649 and EPI_ISL_12363669). 

The most common mutation was the D614G substitution on the S protein and P314L substitution on the NSP12b (RdRp) protein which occurred in all the sequences studied and 67.5% (27/40) showed other amino acid substitution in the S protein including T95I, G339D, S373P, S375F, H655Y, N679K, N764K, D796K, Q954H, and D1146D (Table S2). The second most common amino acid change (39/40; 97.5%) was the F106F substitution on the NSP3 followed by the K417N (31/40; 77.5%) substitution on the S protein T492I substitution on the NSP4, followed by P681H (30/40; 75%), and (29/40; 72.5%) N501Y substitutions on the S protein. In addition to these mutations, several substitutions, deletions, and insertions in other genomic areas were also present (Table S2).

Comparison of mutations on the S protein of the SARS-CoV-2 variants in this study with the wildtype (Wuhan-Hu-1) SARS-CoV-2 revealed that the Omicron variant had the highest number of mutations in this protein compared to the other VOCs in this study. The Omicron variant had 58 amino acid (AA) mutations which included six deletions and four insertions (Table 3). Of the 60 AA mutations in the Omicron variant, 22 were found to be in the RBD of the S protein including G339D, R346K, Y369Y, S371L, S371F, S373P, S375F, T376A, D405N, R408S, K417N, N440K, G446S, T470A, S477N, T478K, E484A, Q493R, G496S, Q498R, N501Y, and Y505H (Table 2 and Table 3). The other AA variations in the RBD included N501Y in Alpha variants, S325P, I326K, V327A, K417N, E484K, and N501Y in the Beta variant and L452R and T478K (Table 3).

## 4. Discussion

In this study, from the 198 samples that were obtained for genomic sequencing in eight districts of the Southern Province of Zambia, 40 SARS-CoV-2 whole genomes were successfully sequenced and analysed. Our dataset revealed that there were more cases of COVID-19 observed in females compared to males. However, other studies have recorded a higher disease burden in males compared to females [44,45,46]. The mean age of patients was 28 with a minimum and maximum age of 0 and 82 years, respectively. However, it cannot be ruled out that the small number of samples analysed in this study may have had an impact on the observed gender distribution and the mean age of COVID-19 patients. Furthermore, lineage assignment revealed that BA.1 was the most prevalent lineage among our sequences followed by B.1.351. This could be explained by the fact that most of the successfully sequenced samples were collected during the Omicron wave. The B.1.351 predominated in the second wave, AY.116 in the third wave, and BA.1 in the fourth wave. The findings corroborate those of other authors who reported the predominance of Beta (B.1.351), Delta (B.1.617.2), and Omicron BA.1 variants in the second, third, and fourth waves of the pandemic in Africa, respectively [13,26,47,48]. Moreover, the detection of AY.116 and B.1.351 coincided with a rapid increase in the number of confirmed cases and deaths in Zambia [35,49]. Despite the small sample size of this study, SARS-CoV-2 lineages were detected in different districts of the Southern Province. The majority of the Omicron variants were detected in Chikankata and Livingstone districts, with the latter having more subvariants. It is plausible that Livingstone, being a border town, a tourist capital, and a major transportation link to Zambia’s neighbouring countries, the area could be at increased risk of the introduction of novel VOCs. Except for the Alpha variant, all the other VOCs detected in this study were found in Choma District. This could be explained by the fact that Macha Research Trust where sequencing was conducted is located in the Choma District and thus the institution was more likely to receive samples throughout the different phases of the COVID-19 waves. 

Phylogenetic analysis revealed that the 40 SARS-CoV-2 genomes generated in this study belonged to four SARS-CoV-2 VOCs namely Alpha, Beta, Delta, and Omicron variants. These VOCs have presented a formidable public health challenge during the COVID-19 pandemic because of their increased viral transmissibility and disease severity [50]. Additionally, the early detection of some of the VOCs in Africa highlights the importance of coordinated molecular surveillance systems in all parts of the world and the role Africa has played in enabling the early detection and characterization of new lineages and informing the global pandemic response. The close phylogenetic relatedness of sequences generated in this study with those from European and African countries supports the idea of possible multiple introductions of the virus from different regions. Phylogenetic analysis further revealed that some sequences from this study clustered together and among other Zambian sequences which may signify the local circulation of these viruses. Notably, sequences obtained in this study that grouped within the Alpha variant clade were phylogenetically distinguishable and were detected in three different districts, which may suggest independent introductions, particularly from Europe, as these sequences were closely related to isolates from England. This introduction could be attributed to the relaxation of flight restrictions at the time these samples were collected. The Zambian Alpha cluster also displayed a longer branch length compared to the other sequences in this clade indicating the continued evolution as the virus circulated. Interestingly, the Alpha variant has not been associated with any COVID-19 wave in Zambia. This observation may suggest that the Alpha variant has no selective advantage over the other VOCs such as the Beta and Delta variants [27]. Although some Beta and Delta variants were closely related to isolates from Europe and Zambia, others showed a close relationship to isolates from Eswatini, DRC, Malawi, and Zimbabwe, suggesting that public health measures implemented by the authorities may have been compromised by porous borders and thus permitting the variants to spread within the region. Phylogenetic analysis also revealed that Omicron variants separated into two major clusters, BA.1 and BA.2, signifying the continued evolution of this VOC. The BA.4 and BA.5 subvariants which have been associated with driving current waves of infection in South Africa [51,52] were not detected in this study.

The S glycoprotein of SARS-CoV-2 plays a pivotal role in viral infection and pathogenesis because of its role in host cell receptor recognition, viral attachment, and entry [53,54,55,56,57]. The present study demonstrated the presence of the D614G mutation in the S protein in all 40 genomes. Similar findings have been reported in many countries including Turkey [58], Oman [59], Egypt [60], and the Comoros Island [61]. In addition to the D614G mutation in the S glycoprotein (23403A > G), a P314L mutation (14408C > T) in the NSP12/RdRp was detected in all the sequences analysed. This finding agrees with previous research which reported a high co-occurrence of these mutations around the globe [62,63,64]. The D614G mutation is associated with a high viral load, infectivity, and transmissibility [50] whereas mutations in the RdRp protein results in a dysfunctional enzyme that generates errors during RNA synthesis, increasing the chances of mutations occurring [62,64,65]. It is also suggested that the co-occurrence of the D614G and NSP12_P314L mutations may enhance viral entry and replication, respectively [66]. Therefore, the S protein mutations and their effects on virulence should be closely monitored and evaluated, as this protein is the main target for vaccine development [67].

Alpha, Beta, and Omicron variants share the N501Y mutation, located in the receptor-binding domain (RBD) of the S protein. It is known to confer an increased binding affinity of the RBD for the ACE2 receptor, raising the viral transmission rate [68]. This mutation was detected in all Alpha, Beta, and 20 Omicron variants of our sequences. Furthermore, the K417N and E484K mutations in the S protein, common to all Beta variants [26] were also detected in our sequences. Other mutations in this present study included the Q27 stop in the ORF8 in all three Alpha variants. This mutation has been observed in the Alpha (B.1.1.7) variant and is known to truncate the ORF8 protein or make it inactive, allowing the accumulation of additional mutations in other regions [68]. Further, eight mutations namely D614G, D950N, F157Δ, L452R, P681R, R158Δ, T19R, and T478K were detected in the S protein in the four sequences of the Delta variant identified in this study. These mutations are identical to those detected in the Indian Delta variants (B.1.617.2) [69]. Deletions, insertions, frameshift variants, and up/downstream variants were much rarer. This observation is also in line with the finding of Malune et al., whose study reported less than 10% of these mutations [70].

Sequences of the Omicron variant obtained in this study were highly mutated, having 149 mutations across the 27 sequences examined. The findings are consistent with the findings of Saxena et al., who detected more mutations in Omicron variants than the Delta variant [71]. When the S protein mutations of the VOCs in this study were compared to the hCoV-19/Wuhan/Hu-1/2019|EPI_ISL_402125, Omicron was highly mutated with 58 mutations and 22 amino acid mutations in the RBD. These mutations are crucial as they are thought to increase the overall risk of reinfection and partial resistance to existing vaccines [72]. In addition to mutations in the S protein, several substitutions and deletions in other genomic regions are also present in all the SARS-CoV-2 variants in this study. Moreover, mutations have an adverse impact on the pathogenicity of SARS-CoV-2 and the development of diagnostic assays, antivirals, and vaccines. Therefore, monitoring of mutations and characterization of their roles in virulence-related conditions in SARS-CoV-2 is very vital in the control and prevention of the spread of the virus.

The limitations of the study are that most of the samples could not be successfully sequenced because they had a Ct > 30, whereas others had poor genomic coverage. We believe poor sample quality was the main reason for the considerably low number of sequences obtained in this study which may have been due to poor storage and transportation conditions (i.e., failure to maintain a good cold chain), as some of the samples came from far-lying rural districts. For improved SARS-CoV-2 genomic surveillance, strengthening the capacity for sample storage and courier in rural areas should be prioritized by the Zambian Ministry of Health. 

## 5. Conclusions

The findings highlighted the circulation of four VOCs in the Southern Province of Zambia namely Alpha (B.1.1.7), Beta (B.1.351), Delta (AY.116), and Omicron (BA.1, BA.1.1, BA.1.14 and BA.2). Phylogenetic analysis revealed that our genomes were closely related to genomes from Europe and Southern Africa indicating intra- and intercontinental introductions of the virus to the country. Additionally, some sequences that clustered with Zambian sequences may signify local transmission of the virus. The Omicron variant exhibited the highest number of amino acid substitutions in the S glycoprotein as compared to the other three variants in this study. Moreover, SARS-CoV-2 with the D614G and P314L mutation was the major circulating virus in Southern Province, Zambia. Our findings stress the need for continued monitoring of SARS-CoV-2 circulation in Zambia, especially in strategically positioned regions such as the Southern Province which could be at increased risk of introduction of novel VOCs. This analysis further represents the first genomic study in the Southern Province of Zambia and highlights the importance of the Zambia Genomic Sequencing Consortium in the expansion of SARS-CoV-2 genomic surveillance in understanding the spread of the virus at national and community levels. It has further contributed to the decentralization of sequencing facilities encompassing among them public, private, and academic public health laboratories which have led to the rapid dissemination of sequences into the public domain.

## Figures and Tables

**Table 1:** Characteristics of the genotyped samples infected with SARS-CoV-2.

Parameters	Sample Distribution *n* (%), Overall, *n* = 198
**Age Group**	
0–14 Years	7 (3.5)
15–50 Years	171 (86.4)
>50 Years	15 (7.6)
Unknown	5 (2.5)
**Gender**	
Female	104 (52.5)
Male	93 (47.0)
Unknown	1 (0.5)

**Table 2:** Distribution of mutations along different genomic regions of SARS-CoV-2 sequences detected in Southern Province.

Genome Segment	Missense Mutation	Synonymous Mutation	Deletion	Insertion	Others	Total Mutation
**Coding Region**						
ORF1ab	74	48	9	3	0	134
Spike	65	3	10	4	0	82
ORF3a	5	4	0	0	0	9
Envelope	5	0	0	0	0	5
Membrane	5	2	0	0	0	7
ORF6	2	2	0	0	0	4
ORF7a	2	0	0	0	0	2
ORF7b	4	1	2	2	0	9
ORF8	4	3	1	0	1 ^1^	9
Nucleocapsid	16	3	1	0	0	20
**Non-coding Region ^2^**						
5′UTR	0	0	0	0	4	4
3′UTR	0	0	0	0	7	7
Total	182	66	23	9	12	292

^1^ Stop codon in the ORF8; ^2^ all the mutations in the non-coding region are extragenic.

**Table 3:** Spike protein mutations in different SARS-CoV-2 variants compared to the wild-type (Wuhan-Hu-1).

SARS-CoV-2 Variants	Spike Mutations ^1^
Wuhan-Hu-1 (wild-type)	-
Alpha (B.1.1.7)	∆H69, ∆Y145, **N501Y**, A570D, D614G, P681H, T716I, T874I, S982A, D1118H
Beta (B.1.351)	L18F, D80A, D215G, ∆L242, T307P, N317F, **S325P, I326K, V327A, K417N, E484K, N501Y**, D614G, A701V, A1087S
Delta (AY.116)	T19R, T95I, G142D, ∆E156, **L452R, T478K,** D614G, P681R, D950N
Omicron (BA.1, BA.1.1, BA.1.14, BA.2)	T19I, ∆L24, ∆A67, ∆A67, ∆I68, T95I, ∆G142, G142D, V193L, Y200C, insI210, ∆N211, N211K, L212C, V213G, insS214, insV213, insR214, insV213, insR214, R214R, A243S, L244S, **G339D, R346K, Y369Y, S371L, S371F, S373P, S375F, T376A, D405N, R408S, K417N, N440K, G446S, T470A, S477N, T478K, E484A, Q493R, G496S, Q498R, N501Y, Y505H,** T547K, D614G, H655Y, N679K, P681H, N764K, D796Y, N856K, Q954H, N969K, L981F, V1104L, D1127G, D1146D, V1264L

^1^ Receptor-binding domain (residues 319–541) is marked as bold in all the variants. Δ Represents deletion, ins represent insertion.

## Data Availability

The sequences have been deposited in the GISAID EpiCoV (https://www.gisaid.org/ (accessed on 8 August 2022) under accession numbers: EPI_ISL_6768069; EPI_ISL_6764745; EPI_ISL_6760707; EPI_ISL_6760905; EPI_ISL_6760973; EPI_ISL_6760998; EPI_ISL_6761015; EPI_ISL_6761027; EPI_ISL_6761052; EPI_ISL_6761088; EPI_ISL_6761100; EPI_ISL_6762977; EPI_ISL_6761106; EPI_ISL_12363645; EPI_ISL_12363646; EPI_ISL_12363647; EPI_ISL_12363648; EPI_ISL_12363649; EPI_ISL_12363652; EPI_ISL_12363653; EPI_ISL_12363654; EPI_ISL_12363658; EPI_ISL_12363660; EPI_ISL_12363661; EPI_ISL_12363662; EPI_ISL_12363663; EPI_ISL_12363664; EPI_ISL_12363667; EPI_ISL_12363668; EPI_ISL_12363669; EPI_ISL_12363670; EPI_ISL_12363671; EPI_ISL_12363672; EPI_ISL_12363673; EPI_ISL_12363674; EPI_ISL_12363681; EPI_ISL_13053241; EPI_ISL_13053244; EPI_ISL_13053875 and EPI_ISL_13053876.

## Supplementary Materials

The following supporting information can be downloaded at: https://www.mdpi.com/article/10.3390/v14091865/s1, Table S1: Characteristics of 40 SARS-CoV-2 whole-genomes from the Southern Province of Zambia; Table S2: Nucleic acid and amino acid mutations observed in the 40 SARS-CoV-2 genomes obtained from the Southern Province of Zambia; Table S3: SARS-CoV-2 primer sequences; Table S4: SARS-CoV-2 genome map.
