# Supplementary material for: Genomic Surveillance of SARS-CoV-2 in the Southern Province of Zambia: Detection and Characterization of Alpha, Beta, Delta, and Omicron Variants of Concern
Source: Viruses. 2022 Aug 24;14(9):1865. doi: 10.3390/v14091865 (PMC9504048; doi:10.3390/v14091865)
Supplement: Supplementary file 1 [file viruses-14-01865-s001.zip › Edited-Supplementary Materials-SARS-CoV-2 Manuscript.pdf]

<u>**Supplementary Materials**</u>

Table S1: Characteristics of 40 SARS-CoV-2 whole-genomes from the Southern Province of Zambia

Patient ID.	Sex	Age	C_T Value	Virus name (GISAID Accession ID)	Collection Date	District	Lineage	Covid-19 Wave
1	Female	17	20.3	hCoV-19/Zambia/SP113/2021|EPI_ISL_6768069	2 February 2021	Choma	B.1.351	Second
2	Male	19	18	hCoV-19/Zambia/SP87/2021|EPI_ISL_6764745	15 February 2021	Namwala	B.1.351	Second
3	Male	38	18.4	hCoV-19/Zambia/SP10/2021|EPI_ISL_6760707	28 January 2021	Mazabuka	B.1.351	Second
4	Male	16	22.3	hCoV-19/Zambia/SP11/2021|EPI_ISL_6760905	3 February 2021	Kalomo	B.1.351	Second
5	Male	34	16.7	hCoV-19/Zambia/SP30/2021|EPI_ISL_6760973	5 February 2021	Namwala	B.1.351	Second
6	Female	17	16.8	hCoV-19/Zambia/SP128/2021|EPI_ISL_6760998	18 February 2021	Choma	B.1.351	Second
7	Male	23	18.2	hCoV-19/Zambia/SP32/2021|EPI_ISL_6761015	15 February 2021	Namwala	B.1.1.7	Second
8	Male	57	19.2	hCoV-19/Zambia/SP37/2020|EPI_ISL_6761027	1 December 2020	Pemba	B.1.1.7	Second
9	Female	44	26.4	hCoV-19/Zambia/SP172/2021|EPI_ISL_6761052	2 September 2021	Chikankata	B.1.1.7	Third
10	Female	23	20.1	hCoV-19/Zambia/SP250/2021|EPI_ISL_6761088	26 June 2021	Choma	AY.116	Third
11	Female	5	28.7	hCoV-19/Zambia/SP252/2021|EPI_ISL_6761100	19 August 2021	Choma	AY.116	Third
12	Male	-	19.5	hCoV-19/Zambia/SP253/2021|EPI_ISL_6762977	17 June 2021	Choma	AY.116	Third
13	Male	44	29.2	hCoV-19/Zambia/SP251/2021|EPI_ISL_6761106	8 September 2021	Choma	AY.116	Third
14	Unknown	0	14.3	hCoV-19/Zambia/CHAZ-CHS1777/2022|EPI_ISL_12363645	Unknown	Chikankata	BA.1	Fourth
15	Male	20	20.5	hCoV-19/Zambia/CHAZ-CHS1778/2022|EPI_ISL_12363646	28 February 2022	Livingstone	BA.2	Fourth
16	Male	0	23	hCoV-19/Zambia/CHAZ-CHS1780/2022|EPI_ISL_12363647	Unknown	Chikankata	BA.1	Fourth
17	Male	46	24.4	hCoV-19/Zambia/CHAZ-CHS1781/2021|EPI_ISL_12363648	21 December 2021	Livingstone	BA.1	Fourth
18	Female	33	26.3	hCoV-19/Zambia/CHAZ-CHS1782/2022|EPI_ISL_12363649	6 April 2022	Choma	BA.2	Fourth
19	Male	32	17.9	hCoV-19/Zambia/CHAZ-CHS1787/2022|EPI_ISL_12363652	5 January 2022	Chikankata	BA.1	Fourth
20	Female	0	28.9	hCoV-19/Zambia/CHAZ-CHS1788/2022|EPI_ISL_12363653	Unknown	Chikankata	BA.1	Fourth

21	Male	17	15	hCoV-19/Zambia/CHAZ-CHS1789/2021|EPI_ISL_12363654	12 December 2021	Livingstone	BA.1	Fourth
22	Female	30	28.9	hCoV-19/Zambia/CHAZ-CHS1794/2021|EPI_ISL_12363658	14 December 2021	Kazungula	BA.2	Fourth
23	Female	52	17.4	hCoV-19/Zambia/CHAZ-CHS1796/2021|EPI_ISL_12363660	7 December 2021	Livingstone	BA.1	Fourth
24	Female	23	22	hCoV-19/Zambia/CHAZ-CHS1798/2021|EPI_ISL_12363661	30 December 2021	Chikankata	BA.1.1	Fourth
25	Male	48	11	hCoV-19/Zambia/CHAZ-CHS1799/2021|EPI_ISL_12363662	28 December 2021	Chikankata	BA.1	Fourth
26	Female	23	28.8	hCoV-19/Zambia/CHAZ-CHS1801/2021|EPI_ISL_12363663	13 December 2021	Livingstone	BA.1.14	Fourth
27	Male	0	28.8	hCoV-19/Zambia/CHAZ-CHS1802/2021|EPI_ISL_12363664	13 December 2021	Livingstone	BA.1	Fourth
28	Male	-	20.5	hCoV-19/Zambia/CHAZ-CHS1807/2021|EPI_ISL_12363667	14 December 2021	Kazungula	BA.1	Fourth
29	Male	40	17.9	hCoV-19/Zambia/CHAZ-CHS1809/2022|EPI_ISL_12363668	20 January 2022	Livingstone	BA.1	Fourth
30	Male	19	25.4	hCoV-19/Zambia/CHAZ-CHS1813/2022|EPI_ISL_12363669	28 February 2022	Livingstone	BA.2	Fourth
31	Male	49	17.3	hCoV-19/Zambia/CHAZ-CHS1815/2022|EPI_ISL_12363670	5 January 2022	Chikankata	BA.1.1	Fourth
32	Female	0	24	hCoV-19/Zambia/CHAZ-CHS1816/2022|EPI_ISL_12363671	Unknown	Chikankata	BA.1	Fourth
33	Male	45	23.7	hCoV-19/Zambia/CHAZ-CHS1817/2021|EPI_ISL_12363672	14 December 2021	Kazungula	BA.1.1	Fourth
34	Male	39	15.2	hCoV-19/Zambia/CHAZ-CHS1818/2021|EPI_ISL_12363673	14 December 2021	Livingstone	BA.1	Fourth
35	Male	39	28.6	hCoV-19/Zambia/CHAZ-CHS1819/2021|EPI_ISL_12363674	14 December 2021	Kazungula	BA.1.1	Fourth
36	Male	25	-	hCoV-19/Zambia/CHAZ-CHS1838/2022|EPI_ISL_12363681	Unknown	Namwala	BA.1	Fourth

37	Male	48	-	hCoV-19/Zambia/CHAZ-CHS1908/2021|EPI_ISL_13053875	28 December 2021	Choma	BA.1	Fourth
38	Female	-	-	>hCoV-19/Zambia/CHAZ-CHS1909/2022|EPI_ISL_13053241	Unknown	Chikankata	BA.1	Fourth
39	Male	19	-	>hCoV-19/Zambia/CHAZ-CHS1979/2022|EPI_ISL_13053876	28 February 2022	Livingstone	BA.2	Fourth
40	Male	46	-	hCoV-19/Zambia/CHAZ-CHS1980/2021|EPI_ISL_13053244	21 December 2021	Livingstone	BA.1	Fourth

Table S2: Nucleic acid and amino acid mutations observed in the 36 SARS-CoV-2 genomes obtained from the Southern Province of Zambia

No.	Position	Genome Region	Number of Mutations	Reference Nucleotide	Sample Nucleotide	AA Change	Frequency	Mutation Type
1	174	**5′UTR**	4	G	T	174	6	extragenic
2	210			G	T	210	4	extragenic
3	222			C	T	222	1	extragenic
4	241			C	T	241	31	extragenic
5	343	**NSP1**	3	.	C	V26	2	Insertion
6	478			C	T	I71I	1	Synonymous
7	670			**T**	**G**	**S135R**	5	**Missense**
8	913	**NSP2**	5	C	T	S36S	3	Synonymous
9	926			**T**	**C**	**F41L**	2	**Missense**
10	940			G	A	K45K	1	Synonymous
11	1059			**C**	**T**	**T85I**	6	**Missense**
12	2692			A	T	T629T	2	Synonymous
13	2790	**NSP3**	42	**C**	**T**	**T24I**	5	**Missense**
14	2832			**A**	**G**	**K38R**	20	**Missense**
15	2903			**A**	**G**	**I62V**	1	**Missense**
16	3037			C	T	F106F	39	Synonymous
17	3177			**C**	**T**	**P153L**	3	**Missense**
18	3267			**C**	**T**	**T183I**	3	**Missense**

19	4150	T	C	V477V	2	Synonymous
20	4181	**G**	**T**	**A488S**	4	**Missense**
21	4184	**G**	**A**	**G489S**	5	**Missense**
22	4255	G	T	P512P	2	Synonymous
23	4276	C	T	Y519Y	2	Synonymous
24	4321	C	T	A534A	5	Synonymous
25	4540	C	T	Y607Y	2	Synonymous
26	4576	A	T	T619T	2	Synonymous
27	4579	T	A	L620L	3	Synonymous
28	4655	**C**	**T**	**R646W**	1	**Missense**
29	5161	**T**	**A**	**F814L**	1	**Missense**
30	5230	**G**	**T**	**K837N**	6	**Missense**
31	5260	T	A	T847T	1	Synonymous
32	5386	T	G	A889A	22	Synonymous
33	5388	**C**	**A**	**A890D**	3	**Missense**
34	5866	C	T	F1049F	1	Synonymous
35	5953	G	A	L1078L	2	Synonymous
36	5986	C	T	F1089F	3	Synonymous
37	6106	T	C	Y1129Y	1	Synonymous
38	6402	**C**	**T**	**P1228L**	4	**Missense**
39	6513	GTT	.	S1265	21	Deletion
40	6568	C	T	D1283D	1	Synonymous
41	6954	**T**	**C**	**I1412T**	3	**Missense**
42	6968	C	T	L1416L	1	Synonymous
43	7069	A	G	E1450E	1	Synonymous
44	7071	**G**	**T**	**G1451V**	1	**Missense**
45	7086	**C**	**T**	**T1456I**	2	**Missense**
46	7124	**C**	**T**	**P1469S**	4	**Missense**
47	7252	**G**	**C**	**L1511F**	1	**Missense**
48	7292	T	C	G1524G	1	Synonymous

49	7787			**G**	**C**	**A1690P**	1	**Missense**
50	7923			**C**	**T**	**S1735F**	1	**Missense**
51	8035			**G**	**T**	**M1772I**	2	**Missense**
52	8290			C	T	L1857L	1	Synonymous
53	8393			**G**	**A**	**A1892T**	22	**Missense**
54	8499			**C**	**T**	**A1927V**	2	**Missense**
55	8723	**NSP4**	9	**A**	**C**	**I57L**	1	**Missense**
56	8986			C	T	D144D	4	Synonymous
57	9053			**G**	**T**	**V167L**	4	**Missense**
58	9344			**C**	**T**	**L264F**	5	**Missense**
59	9424			A	G	V290V	3	Synonymous
60	9474			**C**	**T**	**A307V**	1	**Missense**
61	9534			**C**	**T**	**T327I**	5	**Missense**
62	9866			**C**	**T**	**L438F**	5	**Missense**
63	10029			**C**	**T**	**T492I**	31	**Missense**
64	10181	**NSP5**	7	**A**	**G**	**I43V**	1	**Missense**
65	10198			C	T	D48D	2	Synonymous
66	10323			**A**	**G**	**K90R**	6	**Missense**
67	10447			G	A	R131R	5	Synonymous
68	10449			**C**	**A**	**P132H**	27	**Missense**
69	10691			**A**	**G**	**I213V**	4	**Missense**
70	10892			**A**	**G**	**T280A**	1	**Missense**
71	11083	**NSP6**	9	**G**	**T**	**L37F**	1	**Missense**
72	11109			**C**	**T**	**A46V**	1	**Missense**
73	11201			**A**	**G**	**T77A**	4	**Missense**
74	11286			TGTCTGGTT	.	L105	22	Deletion
75	11288			TCTGGTTTT	.	S106	14	Deletion
76	11332			A	G	V120V	4	Synonymous
77	11537			**A**	**G**	**I189V**	20	**Missense**

78	11653			C	T	L227L	2	Synonymous
79	11713			T	C	S247S	1	Synonymous
80	11941	**NSP7**	1	C	T	V33V	1	Synonymous
81	12406	**NSP8**	4	C	T	N105N	1	Synonymous
82	12452			**C**	**T**	**P121S**	2	**Missense**
83	12473			C	T	K127K	1	Synonymous
84	12557			**A**	**C**	**I156L**	2	**Missense**
85	12786	**NSP9**	2	**C**	**T**	**T34I**	1	**Missense**
86	12880			C	T	I65I	5	Synonymous
87	13195	**NSP10**	2	T	C	V57V	22	Synonymous
88	13339			**T**	**A**	**N105K**	2	**Missense**
89	13592	**NSP12b**	11	**C**	**T**	**T42I**	2	**Missense**
90	13756			**A**	**G**	**I97V**	1	**Missense**
91	14408			**C**	**T**	**P314L**	40	**Missense**
92	14676			C	T	P403P	3	Synonymous
93	15240			C	T	N591N	22	Synonymous
94	15279			C	T	H604H	3	Synonymous
95	15451			**G**	**A**	**G662S**	4	**Missense**
96	15714			C	T	L749L	5	Synonymous
97	15939			T	C	D824D	1	Synonymous
98	15960			C	T	A831A	1	Synonymous
99	16176			T	C	T903T	3	Synonymous
100	16406	**NSP13**	6	**T**	**G**	**V57G**	2	**Missense**
101	16466			**C**	**T**	**P77L**	4	**Missense**
102	16636			**G**	**T**	**A134S**	1	**Missense**
103	17280			G	T	V348V	1	Synonymous
104	17410			**C**	**T**	**R392C**	5	**Missense**
105	17999			**C**	**T**	**T588I**	4	**Missense**
106	18163	**NSP14**	2	**A**	**G**	**I42V**	27	**Missense**

107	19220			**C**	**T**	**A394V**	4	**Missense**
108	19684	**NSP15**	8	**G**	**T**	**V22L**	1	**Missense**
109	19803			C	T	R61R	4	Synonymous
110	19884			C	T	Y88Y	1	Synonymous
111	19955			**C**	**T**	**T112I**	5	**Missense**
112	19999			**G**	**T**	**V127F**	1	**Missense**
113	20055			A	G	E145E	5	Synonymous
114	20094			A	G	K158K	2	Synonymous
115	20132			**C**	**T**	**A171V**	2	**Missense**
116	20741	**NSP16**	23	**A**	**G**	**Q28R**	1	**Missense**
117	21082			**GA**	**CT**	**E142L**	2	**Missense**
118	21110			**C**	**T**	**T151I**	4	**Missense**
119	21512			**A**	**C**	**N285T**	4	**Missense**
120	21517			**AG**	**CA**	**R287Q**	4	**Missense**
121	21520			**G**	**C**	**V288L**	4	**Missense**
122	21523			**GTT**	**TCG**	**V289S**	4	**Missense**
123	21528			T	C	I290I	4	Synonymous
124	21532			**A**	**T**	**S292C**	4	**Missense**
125	21534			.	A	S292	2	Insertion
126	21535			G	.	S292	1	Deletion
127	21536			.	TC	D293	1	Insertion
128	21537			**T**	**A**	**D293E**	1	**Missense**
129	21537			T	.	D293	1	Deletion
130	21539			**T**	**A**	**V294D**	2	**Missense**
131	21542			T	.	L295	1	Deletion
132	21543			**TGTT**	**AACG**	**V296T**	1	**Missense**
133	21543			TGTTA	.	L295	1	Deletion
134	21547			**AA**	**CT**	**N297L**	1	**Missense**
135	21547			AA	.	V296	1	Deletion

136	21549			**C**	**G**	**N297K**	1	**Missense**
137	21550			A	.	N297	1	Deletion
138	21551			**A**	**T**	**N298I**	1	**Missense**
139	21614	**Spike**	82	**C**	**T**	**L18F**	1	**Missense**
140	21618			**C**	**G**	**T19R**	9	**Missense**
141	21618			**C**	**T**	**T19I**	5	**Missense**
142	21633			TACCCCCTG	.	L24	5	Deletion
143	21762			C	.	A67	22	Deletion
144	21764			A	.	A67	22	Deletion
145	21765			TACATG	.	H69	3	Deletion
146	21767			CATG	.	I68	22	Deletion
147	21801			**A**	**C**	**D80A**	6	**Missense**
148	21846			**C**	**T**	**T95I**	26	**Missense**
149	21987			**G**	**A**	**G142D**	9	**Missense**
150	21987			GTGTTTATT	.	G142	22	Deletion
151	21993			ATT	.	Y145	3	Deletion
152	22029			AGTTCA	.	E156	4	Deletion
153	22139			**G**	**C**	**V193L**	1	**Missense**
154	22161			**A**	**G**	**Y200C**	1	**Missense**
155	22193			.	T	I210	16	Insertion
156	22194			ATT	.	N211	2	Deletion
157	22195			**T**	**G**	**N211K**	16	**Missense**
158	22197			**TA**	**GC**	**L212C**	16	**Missense**
159	22200			**T**	**G**	**V213G**	5	**Missense**
160	22201			.	AGC	S214	16	Insertion
161	22202			.	A	V213	1	Insertion
162	22203			.	A	R214	1	Insertion
163	22204			T	A	R214R	16	Synonymous
164	22206			**A**	**G**	**D215G**	6	**Missense**

165	22286	CTTGCTTTA	.	L242	4	Deletion
166	22289	**G**	**T**	**A243S**	1	**Missense**
167	22293	**T**	**C**	**L244S**	1	**Missense**
168	22481	**A**	**C**	**T307P**	1	**Missense**
169	22511	**AA**	**TT**	**N317F**	1	**Missense**
170	22535	**T**	**C**	**S325P**	1	**Missense**
171	22539	**TT**	**AA**	**I326K**	1	**Missense**
172	22542	**T**	**C**	**V327A**	1	**Missense**
173	22578	**G**	**A**	**G339D**	27	**Missense**
174	22599	**G**	**A**	**R346K**	5	**Missense**
175	22669	T	C	Y369Y	1	Synonymous
176	22673	**TC**	**CT**	**S371L**	22	**Missense**
177	22674	**C**	**T**	**S371F**	5	**Missense**
178	22679	**T**	**C**	**S373P**	27	**Missense**
179	22686	**C**	**T**	**S375F**	27	**Missense**
180	22688	**A**	**G**	**T376A**	5	**Missense**
181	22775	**G**	**A**	**D405N**	5	**Missense**
182	22786	**A**	**C**	**R408S**	5	**Missense**
183	22813	**G**	**T**	**K417N**	31	**Missense**
184	22882	**T**	**G**	**N440K**	7	**Missense**
185	22898	**G**	**A**	**G446S**	16	**Missense**
186	22917	**T**	**G**	**L452R**	4	**Missense**
187	22969	**AA**	**GG**	**T470A**	1	**Missense**
188	22992	**G**	**A**	**S477N**	21	**Missense**
189	22995	**C**	**A**	**T478K**	24	**Missense**
190	23012	**G**	**A**	**E484K**	6	**Missense**
191	23013	**A**	**C**	**E484A**	20	**Missense**
192	23040	**A**	**G**	**Q493R**	20	**Missense**
193	23048	**G**	**A**	**G496S**	15	**Missense**
194	23055	**A**	**G**	**Q498R**	20	**Missense**

195	23063			**A**	**T**	**N501Y**	29	**Missense**
196	23075			**T**	**C**	**Y505H**	19	**Missense**
197	23202			**C**	**A**	**T547K**	22	**Missense**
198	23271			**C**	**A**	**A570D**	3	**Missense**
199	23403			**A**	**G**	**D614G**	40	**Missense**
200	23525			**C**	**T**	**H655Y**	27	**Missense**
201	23599			**T**	**G**	**N679K**	27	**Missense**
202	23604			**C**	**G**	**P681R**	29	**Missense**
203	23604			**C**	**A**	**P681H**	5	**Missense**
204	23664			**C**	**T**	**A701V**	6	**Missense**
205	23709			**C**	**T**	**T716I**	3	**Missense**
206	23854			**C**	**A**	**N764K**	27	**Missense**
207	23948			**G**	**T**	**D796Y**	27	**Missense**
208	24130			**C**	**A**	**N856K**	22	**Missense**
209	24183			**C**	**T**	**T874I**	1	**Missense**
210	24410			**G**	**A**	**D950N**	4	**Missense**
211	24424			**A**	**T**	**Q954H**	27	**Missense**
212	24469			**T**	**A**	**N969K**	26	**Missense**
213	24503			**C**	**T**	**L981F**	21	**Missense**
214	24506			**T**	**G**	**S982A**	3	**Missense**
215	24821			**G**	**T**	**A1087S**	1	**Missense**
216	24872			**G**	**T**	**V1104L**	2	**Missense**
217	24914			**G**	**C**	**D1118H**	3	**Missense**
218	24942			**A**	**G**	**D1127G**	1	**Missense**
219	25000			C	T	D1146D	27	Synonymous
220	25352			**G**	**T**	**V1264L**	3	**Missense**
221	25469	**ORF3a**	9	**C**	**T**	**S26L**	4	**Missense**
222	25546			**C**	**T**	**L52F**	4	**Missense**
223	25563			**G**	**T**	**Q57H**	6	**Missense**

224	25584			C	T	T64T	27	Synonymous
225	25587			C	T	L65L	5	Synonymous
226	25665			C	T	Y91Y	1	Synonymous
227	25904			**C**	**T**	**S171L**	6	**Missense**
228	26058			C	T	D222D	1	Synonymous
229	26060			**C**	**T**	**T223I**	5	**Missense**
230	26270	**Envelope**	5	**C**	**T**	**T9I**	27	**Missense**
231	26296			**C**	**A**	**L18I**	1	**Missense**
232	26392			**A**	**T**	**S50C**	1	**Missense**
233	26438			**T**	**C**	**L65P**	2	**Missense**
234	26456			**C**	**T**	**P71L**	6	**Missense**
235	26530	**Membrane**	7	**A**	**G**	**D3G**	19	**Missense**
236	26577			**C**	**G**	**Q19E**	25	**Missense**
237	26709			**G**	**A**	**A63T**	24	**Missense**
238	26767			**T**	**C**	**I82T**	4	**Missense**
239	26858			C	T	F112F	4	Synonymous
240	26997			**T**	**G**	**C159G**	1	**Missense**
241	27059			C	T	Y179Y	4	Synonymous
242	27193	**3′UTR**	1	T	C	27193	1	extragenic
243	27209	**ORF6**	4	**A**	**G**	**H3R**	1	**Missense**
244	27259			A	C	M19M	27	Synonymous
245	27382			**GAT**	**CTC**	**D61L**	5	**Missense**
246	27384			T	C	D61D	1	Synonymous
247	27638	**ORF7a**	2	**T**	**C**	**V82A**	4	**Missense**
248	27752			**C**	**T**	**T120I**	4	**Missense**
249	27807	**ORF7b**	9	C	T	L17L	20	Synonymous
250	27873			.	G	E39	1	Insertion
251	27874			**C**	**A**	**T40N**	1	**Missense**
252	27874			**C**	**T**	**T40I**	4	**Missense**

253	27875			.	C	T40	1	Insertion
254	27879			C	.	C41	1	Deletion
255	27880			**A**	**T**	**H42L**	1	**Missense**
256	27882			G	.	H42	1	Deletion
257	27883			**C**	**T**	**A43V**	1	**Missense**
258	27921	**ORF8**	9	**A**	**T**	**I10F**	1	**Missense**
259	27972			**C**	**T**	**Q27***	3	SNP_stop
260	27998			C	T	D35D	1	Synonymous
261	28048			**G**	**T**	**R52I**	3	**Missense**
262	28079			G	C	V62V	1	Synonymous
263	28111			**A**	**G**	**Y73C**	3	**Missense**
264	28248			**GATTTC**	**.**	**D119**	4	Deletion
265	28253			C	T	F120F	1	Synonymous
266	28253			**CA**	**TC**	**I121L**	5	**Missense**
267	28271	**3′UTR**	2	A	T	28271	27	extragenic
268	28273			A	.	28273	7	extragenic
269	28280	**Nucleocapsid**	20	**GAT**	**CTA**	**D3L**	3	**Missense**
270	28310			**C**	**T**	**P13S**	1	**Missense**
271	28311			**C**	**T**	**P13L**	26	**Missense**
272	28362			**GAGAACGCA**	**.**	**E31**	26	Deletion
273	28461			**A**	**G**	**D63G**	4	**Missense**
274	28881			**G**	**T**	**R203M**	2	**Missense**
275	28881			**GG**	**TT**	**R203I**	2	**Missense**
276	28881			**GGG**	**AAC**	**RG203KR**	30	**Missense**
277	28887			**C**	**T**	**T205I**	6	**Missense**
278	28916			**G**	**T**	**G215C**	4	**Missense**
279	28977			**C**	**T**	**S235F**	3	**Missense**
280	28985			**G**	**T**	**G238C**	1	**Missense**
281	29014			T	C	T247T	1	Synonymous

282	29095			C	T	F274F	1	Synonymous
283	29144			C	T	E290E	1	Synonymous
284	29192			**T**	**C**	**F307L**	2	**Missense**
285	29304			**C**	**T**	**P344L**	5	**Missense**
286	29402			**G**	**T**	**D377Y**	4	**Missense**
287	29433			**A**	**G**	**K387R**	2	**Missense**
288	29510			**A**	**C**	**S413R**	5	**Missense**
289	29545	**3′UTR**	4	C	T	29545	2	extragenic
290	29734			GAGGCCAC GCGGAGTAC GATCGAGTG	.	29734	4	extragenic
291	29736			G	T	29736	1	extragenic
292	29742			G	T	29742	4	extragenic
