# Supplementary material for: Genomic Surveillance of SARS-CoV-2 in the Southern Province of Zambia: Detection and Characterization of Alpha, Beta, Delta, and Omicron Variants of Concern
Source: Viruses. 2022 Aug 24;14(9):1865. doi: 10.3390/v14091865 (PMC9504048; doi:10.3390/v14091865)
Supplement: Supplementary file 1 [file viruses-14-01865-s001.zip › Supplementary Table S4_Primers_mapping.pdf]

Supplementary Material

Table S4: Primer Map Results for linear 29903 residue sequence for Wuhan Seq Sanger V1 primers

```
                                      >>>1_F>>> 35 to 56
  1 ATTAAAGGTTTATACCTTCCCAGGTAACAAACCAACCAACTTTCGATCTCTTGTAGATCT
  1          10        20        30        40        50
  1 TAATTTCCAAATATGGAAGGGTCCATTGTTTGGTTGGTTGAAAGCTAGAGAACATCTAGA
 61 GTTCTCTAAACGAACTTTAAAATCTGTGTGGCTGTCACTCGGCTGCATGCTTAGTGCACT
 61          70        80        90        100       110
 61 CAAGAGATTTGCTTGAAATTTTAGACACACCGACAGTGAGCCGACGTACGAATCACGTGA
121 CACGCAGTATAATTAATAACTAATTACTGTCGTTGACAGGACACGAGTAACTCGTCTATC
121          130       140       150       160       170
121 GTGCGTCATATTAATTATTGATTAATGACAGCAACTGTCCTGTGCTCATTGAGCAGATAG
181 TTCTGCAGGCTGCTTACGGTTTCGTCCGTGTTGCAGCCGATCATCAGCACATCTAGGTTT
181          190       200       210       220       230
181 AAGACGTCCGACGAATGCCAAAGCAGGCACAACGTCGGCTAGTAGTCGTGTAGATCCAAA
241 CGTCCGGGTGTGACCGAAAGGTAAGATGGAGAGCCTTGTCCCTGGTTTCAACGAGAAAAC
241          250       260       270       280       290
241 GCAGGCCCACACTGGCTTTCCATTCTACCTCTCGGAACAGGGACCAAAGTTGCTCTTTTG
301 ACACGTCCAACTCAGTTTGCCTGTTTTACAGGTTCGCGACGTGCTCGTACGTGGCTTTGG
301          310       320       330       340       350
301 TGTGCAGGTTGAGTCAAACGGACAAAATGTCCAAGCGCTGCACGAGCATGCACCGAAACC
                                                      <<<2_R<<< 411 to 432
361 AGACTCCGTGGAGGAGGTCTTATCAGAGGCACGTCAACATCTTAAAGATGGCACTTGTGG
361          370       380       390       400       410
361 TCTGAGGCACCTCCTCCAGAATAGTCTCCGTGCAGTTGTAGAATTTCTACCGTGAACACC
421 CTTAGTAGAAGTTGAAAAAGGCGTTTTGCCTCAACTTGAACAGCCCTATGTGTTCATCAA
421          430       440       450       460       470
421 GAATCATCTTCAACTTTTTCCGCAAAACGGAGTTGAACTTGTCGGGATACACAAGTAGTT
481 ACGTTCGGATGCTCGAACTGCACCTCATGGTCATGTTATGGTTGAGCTGGTAGCAGAACT
481          490       500       510       520       530
481 TGCAAGCCTACGAGCTTGACGTGGAGTACCAGTACAATACCAACTCGACCATCGTCTTGA
541 CGAAGGCATTCAGTACGGTCGTAGTGGTGAGACACTTGGTGTCCTTGTCCCTCATGTGGG
541          550       560       570       580       590
541 GCTTCCGTAAGTCATGCCAGCATCACCACTCTGTGAACCACAGGAACAGGGAGTACACCC
601 CGAAATACCAGTGGCTTACCGCAAGGTTCTTCTTCGTAAGAACGGTAATAAAGGAGCTGG
601          610       620       630       640       650
601 GCTTTATGGTCACCGAATGGCGTTCCAAGAAGAAGCATTCTTGCCATTATTTCCTCGACC
661 TGGCCATAGTTACGGCGCCGATCTAAAGTCATTTGACTTAGGCGACGAGCTTGGCACTGA
661          670       680       690       700       710
661 ACCGGTATCAATGCCGCGGCTAGATTTCAGTAAACTGAATCCGCTGCTCGAACCGTGACT
721 TCCTTATGAAGATTTTCAAGAAAACTGGAACACTAAACATAGCAGTGGTGTTACCCGTGA
721          730       740       750       760       770
721 AGGAATACTTCTAAAAGTTCTTTTGACCTTGTGATTTGTATCGTCACCACAATGGGCACT
781 ACTCATGCGTGAGCTTAACGGAGGGGCATACACTCGCTATGTCGATAACAACTTCTGTGG
781          790       800       810       820       830
781 TGAGTACGCACTCGAATTGCCTCCCCGTATGTGAGCGATACAGCTATTGTTGAAGACACC
                                                             >>>3_F>>> 898 to
917
841 CCCTGATGGCTACCCTCTTGAGTGCATTAAAGACCTTCTAGCACGTGCTGGTAAAGCTTC
841          850       860       870       880       890
841 GGGACTACCGATGGGAGAACTCACGTAATTTCTGGAAGATCGTGCACGACCATTTCGAAG
901 ATGCACTTTGTCCGAACAACTGGACTTTATTGACACTAAGAGGGGTGTATACTGCTGCCG
901          910       920       930       940       950
901 TACGTGAAACAGGCTTGTTGACCTGAAATAACTGTGATTCTCCCCACATATGACGACGGC
              <<<4_R<<< 971 to 991
```

```
 961 TGAACATGAGCATGAAATTGCTTGGTACACGGAACGTTCTGAAAAGAGCTATGAATTGCA
 961          970       980       990      1000      1010
 961 ACTTGTACTCGTACTTTAACGAACCATGTGCCTTGCAAGACTTTTCTCGATACTTAACGT
1021 GACACCTTTTGAAATTAAATTGGCAAAGAAATTTGACACCTTCAATGGGGAATGTCCAAA
1021         1030      1040      1050      1060      1070
1021 CTGTGGAAAACTTTAATTTAACCGTTTCTTTAAACTGTGGAAGTTACCCCTTACAGGTTT
1081 TTTTGTATTTCCCTTAAATTCCATAATCAAGACTATTCAACCAAGGGTTGAAAAGAAAAA
1081         1090      1100      1110      1120      1130
1081 AAAACATAAAGGGAATTTAAGGTATTAGTTCTGATAAGTTGGTTCCCAACTTTTCTTTTT
1141 GCTTGATGGCTTTATGGGTAGAATTCGATCTGTCTATCCAGTTGCGTCACCAAATGAATG
1141         1150      1160      1170      1180      1190
1141 CGAACTACCGAAATACCCATCTTAAGCTAGACAGATAGGTCAACGCAGTGGTTTACTTAC
1201 CAACCAAATGTGCCTTTCAACTCTCATGAAGTGTGATCATTGTGGTGAAACTTCATGGCA
1201         1210      1220      1230      1240      1250
1201 GTTGGTTTACACGGAAAGTTGAGAGTACTTCACACTAGTAACACCACTTTGAAGTACCGT
1261 GACGGGCGATTTTGTTAAAGCCACTTGCGAATTTTGTGGCACTGAGAATTTGACTAAAGA
1261         1270      1280      1290      1300      1310
1261 CTGCCCGCTAAAACAATTTCGGTGAACGCTTAAAACACCGTGACTCTTAAACTGATTTCT
1321 AGGTGCCACTACTTGTGGTTACTTACCCCAAAATGCTGTTGTTAAAATTTATTGTCCAGC
1321         1330      1340      1350      1360      1370
1321 TCCACGGTGATGAACACCAATGAATGGGGTTTTACGACAACAATTTTAAATAACAGGTCG
1381 ATGTCACAATTCAGAAGTAGGACCTGAGCATAGTCTTGCCGAATACCATAATGAATCTGG
1381         1390      1400      1410      1420      1430
1381 TACAGTGTTAAGTCTTCATCCTGGACTCGTATCAGAACGGCTTATGGTATTACTTAGACC
1441 CTTGAAAACCATTCTTCGTAAGGGTGGTCGCACTATTGCCTTTGGAGGCTGTGTGTTCTC
1441         1450      1460      1470      1480      1490
1441 GAACTTTTGGTAAGAAGCATTCCCACCAGCGTGATAACGGAAACCTCCGACACACAAGAG
1501 TTATGTTGGTTGCCATAACAAGTGTGCCTATTGGGTTCCACGTGCTAGCGCTAACATAGG
1501         1510      1520      1530      1540      1550
1501 AATACAACCAACGGTATTGTTCACACGGATAACCCAAGGTGCACGATCGCGATTGTATCC
1561 TTGTAACCATACAGGTGTTGTTGGAGAAGGTTCCGAAGGTCTTAATGACAACCTTCTTGA
1561         1570      1580      1590      1600      1610
1561 AACATTGGTATGTCCACAACAACCTCTTCCAAGGCTTCCAGAATTACTGTTGGAAGAACT
1621 AATACTCCAAAAAGAGAAAGTCAACATCAATATTGTTGGTGACTTTAAACTTAATGAAGA
1621         1630      1640      1650      1660      1670
1621 TTATGAGGTTTTTCTCTTTCAGTTGTAGTTATAACAACCACTGAAATTTGAATTACTTCT
                                                  >>>5_F>>> 1724 to 1743
1681 GATCGCCATTATTTTGGCATCTTTTTCTGCTTCCACAAGTGCTTTTGTGGAAACTGTGAA
1681         1690      1700      1710      1720      1730
1681 CTAGCGGTAATAAAACCGTAGAAAAAGACGAAGGTGTTCACGAAAACACCTTTGACACTT
1741 AGGTTTGGATTATAAAGCATTCAAACAAATTGTTGAATCCTGTGGTAATTTTAAAGTTAC
1741         1750      1760      1770      1780      1790
1741 TCCAAACCTAATATTTCGTAAGTTTGTTTAACAACTTAGGACACCATTAAAATTTCAATG
                                                           <<<6_R<<< 1853 to 1874
1801 AAAAGGAAAAGCTAAAAAAGGTGCCTGGAATATTGGTGAACAGAAATCAATACTGAGTCC
1801         1810      1820      1830      1840      1850
1801 TTTTCCTTTTCGATTTTTTCCACGGACCTTATAACCACTTGTCTTTAGTTATGACTCAGG
1861 TCTTTATGCATTTGCATCAGAGGCTGCTCGTGTTGTACGATCAATTTTCTCCCGCACTCT
1861         1870      1880      1890      1900      1910
1861 AGAAATACGTAAACGTAGTCTCCGACGAGCACAACATGCTAGTTAAAAGAGGGCGTGAGA
1921 TGAAACTGCTCAAAATTCTGTGCGTGTTTTACAGAAGGCCGCTATAACAATACTAGATGG
1921         1930      1940      1950      1960      1970
1921 ACTTTGACGAGTTTTAAGACACGCACAAAATGTCTTCCGGCGATATTGTTATGATCTACC
1981 AATTTCACAGTATTCACTGAGACTCATTGATGCTATGATGTTCACATCTGATTTGGCTAC
1981         1990      2000      2010      2020      2030
1981 TTAAAGTGTCATAAGTGACTCTGAGTAACTACGATACTACAAGTGTAGACTAAACCGATG
2041 TAACAATCTAGTTGTAATGGCCTACATTACAGGTGGTGTTGTTCAGTTGACTTCGCAGTG
2041         2050      2060      2070      2080      2090
2041 ATTGTTAGATCAACATTACCGGATGTAATGTCCACCACAACAAGTCAACTGAAGCGTCAC
```

```
2101 GCTAACTAACATCTTTGGCACTGTTTATGAAAAACTCAAACCCGTCCTTGATTGGCTTGA
2101          2110      2120      2130      2140      2150
2101 CGATTGATTGTAGAAACCGTGACAAATACTTTTTGAGTTTGGGCAGGAACTAACCGAACT
2161 AGAGAAGTTTAAGGAAGGTGTAGAGTTTCTTAGAGACGGTTGGGAAATTGTTAAATTTAT
2161          2170      2180      2190      2200      2210
2161 TCTCTTCAAATTCCTTCCACATCTCAAAGAATCTCTGCCAACCCTTTAACAATTTAAATA
2221 CTCAACCTGTGCTTGTGAAATTGTCGGTGGACAAATTGTCACCTGTGCAAAGGAAATTAA
2221          2230      2240      2250      2260      2270
2221 GAGTTGGACACGAACACTTTAACAGCCACCTGTTTAACAGTGGACACGTTTCCTTTAATT
2281 GGAGAGTGTTCAGACATTCTTTAAGCTTGTAAATAAATTTTTGGCTTTGTGTGCTGACTC
2281          2290      2300      2310      2320      2330
2281 CCTCTCACAAGTCTGTAAGAAATTCGAACATTTATTTAAAAACCGAAACACACGACTGAG
2341 TATCATTATTGGTGGAGCTAAACTTAAAGCCTTGAATTTAGGTGAAACATTTGTCACGCA
2341          2350      2360      2370      2380      2390
2341 ATAGTAATAACCACCTCGATTTGAATTTCGGAACTTAAATCCACTTTGTAAACAGTGCGT
2401 CTCAAAGGGATTGTACAGAAAGTGTGTTAAATCCAGAGAAGAAACTGGCCTACTCATGCC
2401          2410      2420      2430      2440      2450
2401 GAGTTTCCCTAACATGTCTTTCACACAATTTAGGTCTCTTCTTTGACCGGATGAGTACGG
2461 TCTAAAAGCCCCAAAAGAAATTATCTTCTTAGAGGGAGAAACACTTCCCACAGAAGTGTT
2461          2470      2480      2490      2500      2510
2461 AGATTTTCGGGGTTTTCTTTAATAGAAGAATCTCCCTCTTTGTGAAGGGTGTCTTCACAA
                                                >>>7_F>>> 2553 to 2575
2521 AACAGAGGAAGTTGTCTTGAAAACTGGTGATTTACAACCATTAGAACAACCTACTAGTGA
2521          2530      2540      2550      2560      2570
2521 TTGTCTCCTTCAACAGAACTTTTGACCACTAAATGTTGGTAATCTTGTTGGATGATCACT
2581 AGCTGTTGAAGCTCCATTGGTTGGTACACCAGTTTGTATTAACGGGCTTATGTTGCTCGA
2581          2590      2600      2610      2620      2630
2581 TCGACAACTTCGAGGTAACCAACCATGTGGTCAAACATAATTGCCCGAATACAACGAGCT
                                                                   <<<8_R<<< 2691 to 2712
2641 AATCAAAGACACAGAAAAGTACTGTGCCCTTGCACCTAATATGATGGTAACAAACAATAC
2641          2650      2660      2670      2680      2690
2641 TTAGTTTCTGTGTCTTTTCATGACACGGGAACGTGGATTATACTACCATTGTTTGTTATG
2701 CTTCACACTCAAAGGCGGTGCACCAACAAAGGTTACTTTTGGTGATGACACTGTGATAGA
2701          2710      2720      2730      2740      2750
2701 GAAGTGTGAGTTTCCGCCACGTGGTTGTTTCCAATGAAAACCACTACTGTGACACTATCT
2761 AGTGCAAGGTTACAAGAGTGTGAATATCACTTTTGAACTTGATGAAAGGATTGATAAAGT
2761          2770      2780      2790      2800      2810
2761 TCACGTTCCAATGTTCTCACACTTATAGTGAAAACTTGAACTACTTTCCTAACTATTTCA
2821 ACTTAATGAGAAGTGCTCTGCCTATACAGTTGAACTCGGTACAGAAGTAAATGAGTTCGC
2821          2830      2840      2850      2860      2870
2821 TGAATTACTCTTCACGAGACGGATATGTCAACTTGAGCCATGTCTTCATTTACTCAAGCG
2881 CTGTGTTGTGGCAGATGCTGTCATAAAAACTTTGCAACCAGTATCTGAATTACTTACACC
2881          2890      2900      2910      2920      2930
2881 GACACAACACCGTCTACGACAGTATTTTTGAAACGTTGGTCATAGACTTAATGAATGTGG
2941 ACTGGGCATTGATTTAGATGAGTGGAGTATGGCTACATACTACTTATTTGATGAGTCTGG
2941          2950      2960      2970      2980      2990
2941 TGACCCGTAACTAAATCTACTCACCTCATACCGATGTATGATGAATAAACTACTCAGACC
3001 TGAGTTTAAATTGGCTTCACATATGTATTGTTCTTTCTACCCTCCAGATGAGGATGAAGA
3001          3010      3020      3030      3040      3050
3001 ACTCAAATTTAACCGAAGTGTATACATAACAAGAAAGATGGGAGGTCTACTCCTACTTCT
3061 AGAAGGTGATTGTGAAGAAGAAGAGTTTGAGCCATCAACTCAATATGAGTATGGTACTGA
3061          3070      3080      3090      3100      3110
3061 TCTTCCACTAACACTTCTTCTTCTCAAACTCGGTAGTTGAGTTATACTCATACCATGACT
3121 AGATGATTACCAAGGTAAACCTTTGGAATTTGGTGCCACTTCTGCTGCTCTTCAACCTGA
3121          3130      3140      3150      3160      3170
3121 TCTACTAATGGTTCCATTTGGAAACCTTAAACCACGGTGAAGACGACGAGAAGTTGGACT
3181 AGAAGAGCAAGAAGAAGATTGGTTAGATGATGATAGTCAACAAACTGTTGGTCAACAAGA
3181          3190      3200      3210      3220      3230
3181 TCTTCTCGTTCTTCTTCTAACCAATCTACTACTATCAGTTGTTTGACAACCAGTTGTTCT
```

```
    3241 CGGCAGTGAGGACAATCAGACAACTACTATTCAAACAATTGTTGAGGTTCAACCTCAATT
    3241          3250      3260      3270      3280      3290
    3241 GCCGTCACTCCTGTTAGTCTGTTGATGATAAGTTTGTTAACAACTCCAAGTTGGAGTTAA
    3301 AGAGATGGAACTTACACCAGTTGTTCAGACTATTGAAGTGAATAGTTTTAGTGGTTATTT
    3301          3310      3320      3330      3340      3350
    3301 TCTCTACCTTGAATGTGGTCAACAAGTCTGATAACTTCACTTATCAAAATCACCAATAAA
    3361 AAAACTTACTGACAATGTATACATTAAAAATGCAGACATTGTGGAAGAAGCTAAAAAGGT
    3361          3370      3380      3390      3400      3410
    3361 TTTTGAATGACTGTTACATATGTAATTTTTACGTCTGTAACACCTTCTTCGATTTTTCCA
                        >>>9_F>>> 3436 to 3457
    3421 AAAACCAACAGTGGTTGTTAATGCAGCCAATGTTTACCTTAAACATGGAGGAGGTGTTGC
    3421          3430      3440      3450      3460      3470
    3421 TTTTGGTTGTCACCAACAATTACGTCGGTTACAAATGGAATTTGTACCTCCTCCACAACG
                                                          <<<10_R<<< 3535 to
3556
    3481 AGGAGCCTTAAATAAGGCTACTAACAATGCCATGCAAGTTGAATCTGATGATTACATAGC
    3481          3490      3500      3510      3520      3530
    3481 TCCTCGGAATTTATTCCGATGATTGTTACGGTACGTTCAACTTAGACTACTAATGTATCG
    3541 TACTAATGGACCACTTAAAGTGGGTGGTAGTTGTGTTTTAAGCGGACACAATCTTGCTAA
    3541          3550      3560      3570      3580      3590
    3541 ATGATTACCTGGTGAATTTCACCCACCATCAACACAAAATTCGCCTGTGTTAGAACGATT
    3601 ACACTGTCTTCATGTTGTCGGCCCAAATGTTAACAAAGGTGAAGACATTCAACTTCTTAA
    3601          3610      3620      3630      3640      3650
    3601 TGTGACAGAAGTACAACAGCCGGGTTTACAATTGTTTCCACTTCTGTAAGTTGAAGAATT
    3661 GAGTGCTTATGAAAATTTTAATCAGCACGAAGTTCTACTTGCACCATTATTATCAGCTGG
    3661          3670      3680      3690      3700      3710
    3661 CTCACGAATACTTTTAAAATTAGTCGTGCTTCAAGATGAACGTGGTAATAATAGTCGACC
    3721 TATTTTTGGTGCTGACCCTATACATTCTTTAAGAGTTTGTGTAGATACTGTTCGCACAAA
    3721          3730      3740      3750      3760      3770
    3721 ATAAAAACCACGACTGGGATATGTAAGAAATTCTCAAACACATCTATGACAAGCGTGTTT
    3781 TGTCTACTTAGCTGTCTTTGATAAAAATCTCTATGACAAACTTGTTTCAAGCTTTTTGGA
    3781          3790      3800      3810      3820      3830
    3781 ACAGATGAATCGACAGAAACTATTTTTAGAGATACTGTTTGAACAAAGTTCGAAAAACCT
    3841 AATGAAGAGTGAAAAGCAAGTTGAACAAAAGATCGCTGAGATTCCTAAAGAGGAAGTTAA
    3841          3850      3860      3870      3880      3890
    3841 TTACTTCTCACTTTTCGTTCAACTTGTTTTCTAGCGACTCTAAGGATTTCTCCTTCAATT
    3901 GCCATTTATAACTGAAAGTAAACCTTCAGTTGAACAGAGAAAACAAGATGATAAGAAAAT
    3901          3910      3920      3930      3940      3950
    3901 CGGTAAATATTGACTTTCATTTGGAAGTCAACTTGTCTCTTTTGTTCTACTATTCTTTTA
    3961 CAAAGCTTGTGTTGAAGAAGTTACAACAACTCTGGAAGAAACTAAGTTCCTCACAGAAAA
    3961          3970      3980      3990      4000      4010
    3961 GTTTCGAACACAACTTCTTCAATGTTGTTGAGACCTTCTTTGATTCAAGGAGTGTCTTTT
    4021 CTTGTTACTTTATATTGACATTAATGGCAATCTTCATCCAGATTCTGCCACTCTTGTTAG
    4021          4030      4040      4050      4060      4070
    4021 GAACAATGAAATATAACTGTAATTACCGTTAGAAGTAGGTCTAAGACGGTGAGAACAATC
    4081 TGACATTGACATCACTTTCTTAAAGAAAGATGCTCCATATATAGTGGGTGATGTTGTTCA
    4081          4090      4100      4110      4120      4130
    4081 ACTGTAACTGTAGTGAAAGAATTTCTTTCTACGAGGTATATATCACCCACTACAACAAGT
    4141 AGAGGGTGTTTTAACTGCTGTGGTTATACCTACTAAAAAGGCTGGTGGCACTACTGAAAT
    4141          4150      4160      4170      4180      4190
    4141 TCTCCCACAAAATTGACGACACCAATATGGATGATTTTTCCGACCACCGTGATGACTTTA
    4201 GCTAGCGAAAGCTTTGAGAAAAGTGCCAACAGACAATTATATAACCACTTACCCGGGTCA
    4201          4210      4220      4230      4240      4250
    4201 CGATCGCTTTCGAAACTCTTTTCACGGTTGTCTGTTAATATATTGGTGAATGGGCCCAGT
               >>>11_F>>> 4267 to 4288
    4261 GGGTTTAAATGGTTACACTGTAGAGGAGGCAAAGACAGTGCTTAAAAAGTGTAAAAGTGC
    4261          4270      4280      4290      4300      4310
    4261 CCCAAATTTACCAATGTGACATCTCCTCCGTTTCTGTCACGAATTTTTCACATTTTCACG
    4321 CTTTTACATTCTACCATCTATTATCTCTAATGAGAAGCAAGAAATTCTTGGAACTGTTTC
```

```
4321         4330      4340      4350      4360      4370
4321 GAAAATGTAAGATGGTAGATAATAGAGATTACTCTTCGTTCTTTAAGAACCTTGACAAAG
                                    <<<12_R<<< 4409 to 4431
4381 TTGGAATTTGCGAGAAATGCTTGCACATGCAGAAGAAACACGCAAATTAATGCCTGTCTG
4381         4390      4400      4410      4420      4430
4381 AACCTTAAACGCTCTTTACGAACGTGTACGTCTTCTTTGTGCGTTTAATTACGGACAGAC
4441 TGTGGAAACTAAAGCCATAGTTTCAACTATACAGCGTAAATATAAGGGTATTAAAATACA
4441         4450      4460      4470      4480      4490
4441 ACACCTTTGATTTCGGTATCAAAGTTGATATGTCGCATTTATATTCCCATAATTTTATGT
4501 AGAGGGTGTGGTTGATTATGGTGCTAGATTTTACTTTTACACCAGTAAAACAACTGTAGC
4501         4510      4520      4530      4540      4550
4501 TCTCCCACACCAACTAATACCACGATCTAAAATGAAAATGTGGTCATTTTGTTGACATCG
4561 GTCACTTATCAACACACTTAACGATCTAAATGAAACTCTTGTTACAATGCCACTTGGCTA
4561         4570      4580      4590      4600      4610
4561 CAGTGAATAGTTGTGTGAATTGCTAGATTTACTTTGAGAACAATGTTACGGTGAACCGAT
4621 TGTAACACATGGCTTAAATTTGGAAGAAGCTGCTCGGTATATGAGATCTCTCAAAGTGCC
4621         4630      4640      4650      4660      4670
4621 ACATTGTGTACCGAATTTAAACCTTCTTCGACGAGCCATATACTCTAGAGAGTTTCACGG
4681 AGCTACAGTTTCTGTTTCTTCACCTGATGCTGTTACAGCGTATAATGGTTATCTTACTTC
4681         4690      4700      4710      4720      4730
4681 TCGATGTCAAAGACAAAGAAGTGGACTACGACAATGTCGCATATTACCAATAGAATGAAG
4741 TTCTTCTAAAACACCTGAAGAACATTTTATTGAAACCATCTCACTTGCTGGTTCCTATAA
4741         4750      4760      4770      4780      4790
4741 AAGAAGATTTTGTGGACTTCTTGTAAAATAACTTTGGTAGAGTGAACGACCAAGGATATT
4801 AGATTGGTCCTATTCTGGACAATCTACACAACTAGGTATAGAATTTCTTAAGAGAGGTGA
4801         4810      4820      4830      4840      4850
4801 TCTAACCAGGATAAGACCTGTTAGATGTGTTGATCCATATCTTAAAGAATTCTCTCCACT
4861 TAAAAGTGTATATTACACTAGTAATCCTACCACATTCCACCTAGATGGTGAAGTTATCAC
4861         4870      4880      4890      4900      4910
4861 ATTTTCACATATAATGTGATCATTAGGATGGTGTAAGGTGGATCTACCACTTCAATAGTG
4921 CTTTGACAATCTTAAGACACTTCTTTCTTTGAGAGAAGTGAGGACTATTAAGGTGTTTAC
4921         4930      4940      4950      4960      4970
4921 GAAACTGTTAGAATTCTGTGAAGAAAGAAACTCTCTTCACTCCTGATAATTCCACAAATG
4981 AACAGTAGACAACATTAACCTCCACACGCAAGTTGTGGACATGTCAATGACATATGGACA
4981         4990      5000      5010      5020      5030
4981 TTGTCATCTGTTGTAATTGGAGGTGTGCGTTCAACACCTGTACAGTTACTGTATACCTGT
5041 ACAGTTTGGTCCAACTTATTTGGATGGAGCTGATGTTACTAAAATAAAACCTCATAATTC
5041         5050      5060      5070      5080      5090
5041 TGTCAAACCAGGTTGAATAAACCTACCTCGACTACAATGATTTTATTTTGGAGTATTAAG
                                 >>>13_F>>> 5126 to 5148
5101 ACATGAAGGTAAAACATTTTATGTTTTACCTAATGATGACACTCTACGTGTTGAGGCTTT
5101         5110      5120      5130      5140      5150
5101 TGTACTTCCATTTTGTAAAATACAAAATGGATTACTACTGTGAGATGCACAACTCCGAAA
5161 TGAGTACTACCACACAACTGATCCTAGTTTTCTGGGTAGGTACATGTCAGCATTAAATCA
5161         5170      5180      5190      5200      5210
5161 ACTCATGATGGTGTGTTGACTAGGATCAAAAGACCCATCCATGTACAGTCGTAATTTAGT
                 <<<14_R<<< 5232 to 5253
5221 CACTAAAAAGTGGAAATACCCACAAGTTAATGGTTTAACTTCTATTAAATGGGCAGATAA
5221         5230      5240      5250      5260      5270
5221 GTGATTTTTCACCTTTATGGGTGTTCAATTACCAAATTGAAGATAATTTACCCGTCTATT
5281 CAACTGTTATCTTGCCACTGCATTGTTAACACTCCAACAAATAGAGTTGAAGTTTAATCC
5281         5290      5300      5310      5320      5330
5281 GTTGACAATAGAACGGTGACGTAACAATTGTGAGGTTGTTTATCTCAACTTCAAATTAGG
5341 ACCTGCTCTACAAGATGCTTATTACAGAGCAAGGGCTGGTGAAGCTGCTAACTTTTGTGC
5341         5350      5360      5370      5380      5390
5341 TGGACGAGATGTTCTACGAATAATGTCTCGTTCCCGACCACTTCGACGATTGAAAACACG
5401 ACTTATCTTAGCCTACTGTAATAAGACAGTAGGTGAGTTAGGTGATGTTAGAGAAACAAT
5401         5410      5420      5430      5440      5450
5401 TGAATAGAATCGGATGACATTATTCTGTCATCCACTCAATCCACTACAATCTCTTTGTTA
```

```
5461 GAGTTACTTGTTTCAACATGCCAATTTAGATTCTTGCAAAAGAGTCTTGAACGTGGTGTG
5461          5470      5480      5490      5500      5510
5461 CTCAATGAACAAAGTTGTACGGTTAAATCTAAGAACGTTTTCTCAGAACTTGCACCACAC
5521 TAAAACTTGTGGACAACAGCAGACAACCCTTAAGGGTGTAGAAGCTGTTATGTACATGGG
5521          5530      5540      5550      5560      5570
5521 ATTTTGAACACCTGTTGTCGTCTGTTGGGAATTCCCACATCTTCGACAATACATGTACCC
5581 CACACTTTCTTATGAACAATTTAAGAAAGGTGTTCAGATACCTTGTACGTGTGGTAAACA
5581          5590      5600      5610      5620      5630
5581 GTGTGAAAGAATACTTGTTAAATTCTTTCCACAAGTCTATGGAACATGCACACCATTTGT
5641 AGCTACAAAATATCTAGTACAACAGGAGTCACCTTTTGTTATGATGTCAGCACCACCTGC
5641          5650      5660      5670      5680      5690
5641 TCGATGTTTTATAGATCATGTTGTCCTCAGTGGAAAACAATACTACAGTCGTGGTGGACG
5701 TCAGTATGAACTTAAGCATGGTACATTTACTTGTGCTAGTGAGTACACTGGTAATTACCA
5701          5710      5720      5730      5740      5750
5701 AGTCATACTTGAATTCGTACCATGTAAATGAACACGATCACTCATGTGACCATTAATGGT
5761 GTGTGGTCACTATAAACATATAACTTCTAAAGAAACTTTGTATTGCATAGACGGTGCTTT
5761          5770      5780      5790      5800      5810
5761 CACACCAGTGATATTTGTATATTGAAGATTTCTTTGAAACATAACGTATCTGCCACGAAA
5821 ACTTACAAAGTCCTCAGAATACAAAGGTCCTATTACGGATGTTTTCTACAAAGAAAACAG
5821          5830      5840      5850      5860      5870
5821 TGAATGTTTCAGGAGTCTTATGTTTCCAGGATAATGCCTACAAAAGATGTTTCTTTTGTC
                                     >>>15_F>>> 5913 to 5934
5881 TTACACAACAACCATAAAACCAGTTACTTATAAATTGGATGGTGTTGTTTGTACAGAAAT
5881          5890      5900      5910      5920      5930
5881 AATGTGTTGTTGGTATTTTGGTCAATGAATATTTAACCTACCACAACAAACATGTCTTTA
5941 TGACCCTAAGTTGGACAATTATTATAAGAAAGACAATTCTTATTTCACAGAGCAACCAAT
5941          5950      5960      5970      5980      5990
5941 ACTGGGATTCAACCTGTTAATAATATTCTTTCTGTTAAGAATAAAGTGTCTCGTTGGTTA
6001 TGATCTTGTACCAAACCAACCATATCCAAACGCAAGCTTCGATAATTTTAAGTTTGTATG
6001          6010      6020      6030      6040      6050
6001 ACTAGAACATGGTTTGGTTGGTATAGGTTTGCGTTCGAAGCTATTAAAATTCAAACATAC
                                              <<<16_R<<< 6102 to 6125
6061 TGATAATATCAAATTTGCTGATGATTTAAACCAGTTAACTGGTTATAAGAAACCTGCTTC
6061          6070      6080      6090      6100      6110
6061 ACTATTATAGTTTAAACGACTACTAAATTTGGTCAATTGACCAATATTCTTTGGACGAAG
6121 AAGAGAGCTTAAAGTTACATTTTTCCCTGACTTAAATGGTGATGTGGTGGCTATTGATTA
6121          6130      6140      6150      6160      6170
6121 TTCTCTCGAATTTCAATGTAAAAAGGGACTGAATTTACCACTACACCACCGATAACTAAT
6181 TAAACACTACACACCCTCTTTTAAGAAAGGAGCTAAATTGTTACATAAACCTATTGTTTG
6181          6190      6200      6210      6220      6230
6181 ATTTGTGATGTGTGGGAGAAAATTCTTTCCTCGATTTAACAATGTATTTGGATAACAAAC
6241 GCATGTTAACAATGCAACTAATAAAGCCACGTATAAACCAAATACCTGGTGTATACGTTG
6241          6250      6260      6270      6280      6290
6241 CGTACAATTGTTACGTTGATTATTTCGGTGCATATTTGGTTTATGGACCACATATGCAAC
6301 TCTTTGGAGCACAAAACCAGTTGAAACATCAAATTCGTTTGATGTACTGAAGTCAGAGGA
6301          6310      6320      6330      6340      6350
6301 AGAAACCTCGTGTTTTGGTCAACTTTGTAGTTTAAGCAAACTACATGACTTCAGTCTCCT
6361 CGCGCAGGGAATGGATAATCTTGCCTGCGAAGATCTAAAACCAGTCTCTGAAGAAGTAGT
6361          6370      6380      6390      6400      6410
6361 GCGCGTCCCTTACCTATTAGAACGGACGCTTCTAGATTTTGGTCAGAGACTTCTTCATCA
6421 GGAAAATCCTACCATACAGAAAGACGTTCTTGAGTGTAATGTGAAAACTACCGAAGTTGT
6421          6430      6440      6450      6460      6470
6421 CCTTTTAGGATGGTATGTCTTTCTGCAAGAACTCACATTACACTTTTGATGGCTTCAACA
6481 AGGAGACATTATACTTAAACCAGCAAATAATAGTTTAAAAATTACAGAAGAGGTTGGCCA
6481          6490      6500      6510      6520      6530
6481 TCCTCTGTAATATGAATTTGGTCGTTTATTATCAAATTTTTAATGTCTTCTCCAACCGGT
6541 CACAGATCTAATGGCTGCTTATGTAGACAATTCTAGTCTTACTATTAAGAAACCTAATGA
6541          6550      6560      6570      6580      6590
6541 GTGTCTAGATTACCGACGAATACATCTGTTAAGATCAGAATGATAATTCTTTGGATTACT
```

```
6601 ATTATCTAGAGTATTAGGTTTGAAAACCCTTGCTACTCATGGTTTAGCTGCTGTTAATAG
6601           6610      6620      6630      6640      6650
6601 TAATAGATCTCATAATCCAAACTTTTGGGAACGATGAGTACCAAATCGACGACAATTATC
6661 TGTCCCTTGGGATACTATAGCTAATTATGCTAAGCCTTTTCTTAACAAAGTTGTTAGTAC
6661           6670      6680      6690      6700      6710
6661 ACAGGGAACCCTATGATATCGATTAATACGATTCGGAAAAGAATTGTTTCAACAATCATG
                                   >>>17_F>>> 6750 to 6774
6721 AACTACTAACATAGTTACACGGTGTTTAAACCGTGTTTGTACTAATTATATGCCTTATTT
6721           6730      6740      6750      6760      6770
6721 TTGATGATTGTATCAATGTGCCACAAATTTGGCACAAACATGATTAATATACGGAATAAA
6781 CTTTACTTTATTGCTACAATTGTGTACTTTTACTAGAAGTACAAATTCTAGAATTAAAGC
6781           6790      6800      6810      6820      6830
6781 GAAATGAAATAACGATGTTAACACATGAAAATGATCTTCATGTTTAAGATCTTAATTTCG
                              <<<18_R<<< 6865 to 6886
6841 ATCTATGCCGACTACTATAGCAAAGAATACTGTTAAGAGTGTCGGTAAATTTTGTCTAGA
6841           6850      6860      6870      6880      6890
6841 TAGATACGGCTGATGATATCGTTTCTTATGACAATTCTCACAGCCATTTAAAACAGATCT
6901 GGCTTCATTTAATTATTTGAAGTCACCTAATTTTTCTAAACTGATAAATATTATAATTTG
6901           6910      6920      6930      6940      6950
6901 CCGAAGTAAATTAATAAACTTCAGTGGATTAAAAAGATTTGACTATTTATAATATTAAAC
6961 GTTTTTACTATTAAGTGTTTGCCTAGGTTCTTTAATCTACTCAACCGCTGCTTTAGGTGT
6961           6970      6980      6990      7000      7010
6961 CAAAAATGATAATTCACAAACGGATCCAAGAAATTAGATGAGTTGGCGACGAAATCCACA
7021 TTTAATGTCTAATTTAGGCATGCCTTCTTACTGTACTGGTTACAGAGAAGGCTATTTGAA
7021           7030      7040      7050      7060      7070
7021 AAATTACAGATTAAATCCGTACGGAAGAATGACATGACCAATGTCTCTTCCGATAAACTT
7081 CTCTACTAATGTCACTATTGCAACCTACTGTACTGGTTCTATACCTTGTAGTGTTTGTCT
7081           7090      7100      7110      7120      7130
7081 GAGATGATTACAGTGATAACGTTGGATGACATGACCAAGATATGGAACATCACAAACAGA
7141 TAGTGGTTTAGATTCTTTAGACACCTATCCTTCTTTAGAAACTATACAAATTACCATTTC
7141           7150      7160      7170      7180      7190
7141 ATCACCAAATCTAAGAAATCTGTGGATAGGAAGAAATCTTTGATATGTTTAATGGTAAAG
7201 ATCTTTTAAATGGGATTTAACTGCTTTTGGCTTAGTTGCAGAGTGGTTTTTGGCATATAT
7201           7210      7220      7230      7240      7250
7201 TAGAAAATTTACCCTAAATTGACGAAAACCGAATCAACGTCTCACCAAAAACCGTATATA
7261 TCTTTTCACTAGGTTTTTCTATGTACTTGGATTGGCTGCAATCATGCAATTGTTTTTCAG
7261           7270      7280      7290      7300      7310
7261 AGAAAAGTGATCCAAAAAGATACATGAACCTAACCGACGTTAGTACGTTAACAAAAAGTC
7321 CTATTTTGCAGTACATTTTATTAGTAATTCTTGGCTTATGTGGTTAATAATTAATCTTGT
7321           7330      7340      7350      7360      7370
7321 GATAAAACGTCATGTAAAATAATCATTAAGAACCGAATACACCAATTATTAATTAGAACA
7381 ACAAATGGCCCCGATTTCAGCTATGGTTAGAATGTACATCTTCTTTGCATCATTTTATTA
7381           7390      7400      7410      7420      7430
7381 TGTTTACCGGGGCTAAAGTCGATACCAATCTTACATGTAGAAGAAACGTAGTAAAATAAT
7441 TGTATGGAAAAGTTATGTGCATGTTGTAGACGGTTGTAATTCATCAACTTGTATGATGTG
7441           7450      7460      7470      7480      7490
7441 ACATACCTTTTCAATACACGTACAACATCTGCCAACATTAAGTAGTTGAACATACTACAC
               >>>19_F>>> 7515 to 7536
7501 TTACAAACGTAATAGAGCAACAAGAGTCGAATGTACAACTATTGTTAATGGTGTTAGAAG
7501           7510      7520      7530      7540      7550
7501 AATGTTTGCATTATCTCGTTGTTCTCAGCTTACATGTTGATAACAATTACCACAATCTTC
7561 GTCCTTTTATGTCTATGCTAATGGAGGTAAAGGCTTTTGCAAACTACACAATTGGAATTG
7561           7570      7580      7590      7600      7610
7561 CAGGAAAATACAGATACGATTACCTCCATTTCCGAAAACGTTTGATGTGTTAACCTTAAC
                                                  <<<20_R<<< 7662 to 7682
7621 TGTTAATTGTGATACATTCTGTGCTGGTAGTACATTTATTAGTGATGAAGTTGCGAGAGA
7621           7630      7640      7650      7660      7670
7621 ACAATTAACACTATGTAAGACACGACCATCATGTAAATAATCACTACTTCAACGCTCTCT
7681 CTTGTCACTACAGTTTAAAAGACCAATAAATCCTACTGACCAGTCTTCTTACATCGTTGA
```

```
7681          7690       7700       7710       7720       7730
7681 GAACAGTGATGTCAAATTTTCTGGTTATTTAGGATGACTGGTCAGAAGAATGTAGCAACT
7741 TAGTGTTACAGTGAAGAATGGTTCCATCCATCTTTACTTTGATAAAGCTGGTCAAAAGAC
7741          7750       7760       7770       7780       7790
7741 ATCACAATGTCACTTCTTACCAAGGTAGGTAGAAATGAAACTATTTCGACCAGTTTTCTG
7801 TTATGAAAGACATTCTCTCTCTCATTTTGTTAACTTAGACAACCTGAGAGCTAATAACAC
7801          7810       7820       7830       7840       7850
7801 AATACTTTCTGTAAGAGAGAGAGTAAAACAATTGAATCTGTTGGACTCTCGATTATTGTG
7861 TAAAGGTTCATTGCCTATTAATGTTATAGTTTTTGATGGTAAATCAAAATGTGAAGAATC
7861          7870       7880       7890       7900       7910
7861 ATTTCCAAGTAACGGATAATTACAATATCAAAAACTACCATTTAGTTTTACACTTCTTAG
7921 ATCTGCAAAATCAGCGTCTGTTTACTACAGTCAGCTTATGTGTCAACCTATACTGTTACT
7921          7930       7940       7950       7960       7970
7921 TAGACGTTTTAGTCGCAGACAAATGATGTCAGTCGAATACACAGTTGGATATGACAATGA
7981 AGATCAGGCATTAGTGTCTGATGTTGGTGATAGTGCGGAAGTTGCAGTTAAAATGTTTGA
7981          7990       8000       8010       8020       8030
7981 TCTAGTCCGTAATCACAGACTACAACCACTATCACGCCTTCAACGTCAATTTTACAAACT
8041 TGCTTACGTTAATACGTTTCATCAACTTTTAACGTACCAATGGAAAAACTCAAAACACT
8041          8050       8060       8070       8080       8090
8041 ACGAATGCAATTATGCAAAAGTAGTTGAAAATTGCATGGTTACCTTTTTGAGTTTTGTGA
8101 AGTTGCAACTGCAGAAGCTGAACTTGCAAAGAATGTGTCCTTAGACAATGTCTTATCTAC
8101          8110       8120       8130       8140       8150
8101 TCAACGTTGACGTCTTCGACTTGAACGTTTCTTACACAGGAATCTGTTACAGAATAGATG
8161 TTTTATTTCAGCAGCTCGGCAAGGGTTTGTTGATTCAGATGTAGAAACTAAAGATGTTGT
8161          8170       8180       8190       8200       8210
8161 AAAATAAAGTCGTCGAGCCGTTCCCAAACAACTAAGTCTACATCTTTGATTTCTACAACA
8221 TGAATGTCTTAAATTGTCACATCAATCTGACATAGAAGTTACTGGCGATAGTTGTAATAA
8221          8230       8240       8250       8260       8270
8221 ACTTACAGAATTTAACAGTGTAGTTAGACTGTATCTTCAATGACCGCTATCAACATTATT
8281 CTATATGCTCACCTATAACAAAGTTGAAAACATGACACCCCGTGACCTTGGTGCTTGTAT
8281          8290       8300       8310       8320       8330
8281 GATATACGAGTGGATATTGTTTCAACTTTTGTACTGTGGGGCACTGGAACCACGAACATA
8341 TGACTGTAGTGCGCGTCATATTAATGCGCAGGTAGCAAAAAGTCACAACATTGCTTTGAT
8341          8350       8360       8370       8380       8390
8341 ACTGACATCACGCGCAGTATAATTACGCGTCCATCGTTTTTCAGTGTTGTAACGAAACTA
      >>>21_F>>> 8402 to 8424
8401 ATGGAACGTTAAAGATTTCATGTCATTGTCTGAACAACTACGAAAACAAATACGTAGTGC
8401          8410       8420       8430       8440       8450
8401 TACCTTGCAATTTCTAAAGTACAGTAACAGACTTGTTGATGCTTTTGTTTATGCATCACG
                                     <<<22_R<<< 8491 to 8513
8461 TGCTAAAAAGAATAACTTACCTTTTAAGTTGACATGTGCAACTACTAGACAAGTTGTTAA
8461          8470       8480       8490       8500       8510
8461 ACGATTTTTCTTATTGAATGGAAAATTCAACTGTACACGTTGATGATCTGTTCAACAATT
8521 TGTTGTAACAACAAAGATAGCACTTAAGGGTGGTAAAATTGTTAATAATTGGTTGAAGCA
8521          8530       8540       8550       8560       8570
8521 ACAACATTGTTGTTTCTATCGTGAATTCCCACCATTTTAACAATTATTAACCAACTTCGT
8581 GTTAATTAAAGTTACACTTGTGTTCCTTTTTGTTGCTGCTATTTTCTATTTAATAACACC
8581          8590       8600       8610       8620       8630
8581 CAATTAATTTCAATGTGAACACAAGGAAAAACAACGACGATAAAAGATAAATTATTGTGG
8641 TGTTCATGTCATGTCTAAACATACTGACTTTTCAAGTGAAATCATAGGATACAAGGCTAT
8641          8650       8660       8670       8680       8690
8641 ACAAGTACAGTACAGATTTGTATGACTGAAAAGTTCACTTTAGTATCCTATGTTCCGATA
8701 TGATGGTGGTGTCACTCGTGACATAGCATCTACAGATACTTGTTTTGCTAACAAACATGC
8701          8710       8720       8730       8740       8750
8701 ACTACCACCACAGTGAGCACTGTATCGTAGATGTCTATGAACAAAACGATTGTTTGTACG
8761 TGATTTTGACACATGGTTTAGCCAGCGTGGTGGTAGTTATACTAATGACAAAGCTTGCCC
8761          8770       8780       8790       8800       8810
8761 ACTAAAACTGTGTACCAAATCGGTCGCACCACCATCAATATGATTACTGTTTCGAACGGG
8821 ATTGATTGCTGCAGTCATAACAAGAGAAGTGGGTTTTGTCGTGCCTGGTTTGCCTGGCAC
```

```
8821          8830      8840      8850      8860      8870
8821 TAACTAACGACGTCAGTATTGTTCTCTTCACCCAAAACAGCACGGACCAAACGGACCGTG
8881 GATATTACGCACAACTAATGGTGACTTTTTGCATTTCTTACCTAGAGTTTTTAGTGCAGT
8881          8890      8900      8910      8920      8930
8881 CTATAATGCGTGTTGATTACCACTGAAAAACGTAAAGAATGGATCTCAAAAATCACGTCA
8941 TGGTAACATCTGTTACACACCATCAAAACTTATAGAGTACACTGACTTTGCAACATCAGC
8941          8950      8960      8970      8980      8990
8941 ACCATTGTAGACAATGTGTGGTAGTTTTGAATATCTCATGTGACTGAAACGTTGTAGTCG
9001 TTGTGTTTTGGCTGCTGAATGTACAATTTTTAAAGATGCTTCTGGTAAGCCAGTACCATA
9001          9010      9020      9030      9040      9050
9001 AACACAAAACCGACGACTTACATGTTAAAAATTTCTACGAAGACCATTCGGTCATGGTAT
9061 TTGTTATGATACCAATGTACTAGAAGGTTCTGTTGCTTATGAAAGTTTACGCCCTGACAC
9061          9070      9080      9090      9100      9110
9061 AACAATACTATGGTTACATGATCTTCCAAGACAACGAATACTTTCAAATGCGGGACTGTG
9121 ACGTTATGTGCTCATGGATGGCTCTATTATTCAATTTCCTAACACCTACCTTGAAGGTTC
9121          9130      9140      9150      9160      9170
9121 TGCAATACACGAGTACCTACCGAGATAATAAGTTAAAGGATTGTGGATGGAACTTCCAAG
                              >>>23_F>>> 9203 to 9224
9181 TGTTAGAGTGGTAACAACTTTTGATTCTGAGTACTGTAGGCACGGCACTTGTGAAAGATC
9181          9190      9200      9210      9220      9230
9181 ACAATCTCACCATTGTTGAAAACTAAGACTCATGACATCCGTGCCGTGAACACTTTCTAG
9241 AGAAGCTGGTGTTTGTGTATCTACTAGTGGTAGATGGGTACTTAACAATGATTATTACAG
9241          9250      9260      9270      9280      9290
9241 TCTTCGACCACAAACACATAGATGATCACCATCTACCCATGAATTGTTACTAATAATGTC
                          <<<24_R<<< 9319 to 9340
9301 ATCTTTACCAGGAGTTTTCTGTGGTGTAGATGCTGTAAATTTACTTACTAATATGTTTAC
9301          9310      9320      9330      9340      9350
9301 TAGAAATGGTCCTCAAAAGACACCACATCTACGACATTTAAATGAATGATTATACAAATG
9361 ACCACTAATTCAACCTATTGGTGCTTTGGACATATCAGCATCTATAGTAGCTGGTGGTAT
9361          9370      9380      9390      9400      9410
9361 TGGTGATTAAGTTGGATAACCACGAAACCTGTATAGTCGTAGATATCATCGACCACCATA
9421 TGTAGCTATCGTAGTAACATGCCTTGCCTACTATTTTATGAGGTTTAGAAGAGCTTTTGG
9421          9430      9440      9450      9460      9470
9421 ACATCGATAGCATCATTGTACGGAACGGATGATAAAATACTCCAAATCTTCTCGAAAACC
9481 TGAATACAGTCATGTAGTTGCCTTTAATACTTTACTATTCCTTATGTCATTCACTGTACT
9481          9490      9500      9510      9520      9530
9481 ACTTATGTCAGTACATCAACGGAAATTATGAAATGATAAGGAATACAGTAAGTGACATGA
9541 CTGTTTAACACCAGTTTACTCATTCTTACCTGGTGTTTATTCTGTTATTTACTTGTACTT
9541          9550      9560      9570      9580      9590
9541 GACAAATTGTGGTCAAATGAGTAAGAATGGACCACAAATAAGACAATAAATGAACATGAA
9601 GACATTTTATCTTACTAATGATGTTTCTTTTTTAGCACATATTCAGTGGATGGTTATGTT
9601          9610      9620      9630      9640      9650
9601 CTGTAAAATAGAATGATTACTACAAAGAAAAAATCGTGTATAAGTCACCTACCAATACAA
9661 CACACCTTTAGTACCTTTCTGGATAACAATTGCTTATATCATTTGTATTTCCACAAAGCA
9661          9670      9680      9690      9700      9710
9661 GTGTGGAAATCATGGAAAGACCTATTGTTAACGAATATAGTAAACATAAAGGTGTTTCGT
9721 TTTCTATTGGTTCTTTAGTAATTACCTAAAGAGACGTGTAGTCTTTAATGGTGTTTCCTT
9721          9730      9740      9750      9760      9770
9721 AAAGATAACCAAGAAATCATTAATGGATTTCTCTGCACATCAGAAATTACCACAAAGGAA
9781 TAGTACTTTTGAAGAAGCTGCGCTGTGCACCTTTTTGTTAAATAAAGAAATGTATCTAAA
9781          9790      9800      9810      9820      9830
9781 ATCATGAAAACTTCTTCGACGCGACACGTGGAAAAACAATTTATTTCTTTACATAGATTT
9841 GTTGCGTAGTGATGTGCTATTACCTCTTACGCAATATAATAGATACTTAGCTCTTTATAA
9841          9850      9860      9870      9880      9890
9841 CAACGCATCACTACACGATAATGGAGAATGCGTTATATTATCTATGAATCGAGAAATATT
9901 TAAGTACAAGTATTTTAGTGGAGCAATGGATACAACTAGCTACAGAGAAGCTGCTTGTTG
9901          9910      9920      9930      9940      9950
9901 ATTCATGTTCATAAAATCACCTCGTTACCTATGTTGATCGATGTCTCTTCGACGAACAAC
9961 TCATCTCGCAAAGGCTCTCAATGACTTCAGTAACTCAGGTTCTGATGTTCTTTACCAACC
```

```
     9961          9970      9980      9990      10000     10010
     9961 AGTAGAGCGTTTCCGAGAGTTACTGAAGTCATTGAGTCCAAGACTACAAGAAATGGTTGG
            >>>25_F>>> 10022 to 10043
    10021 ACCACAAACCTCTATCACCTCAGCTGTTTTGCAGAGTGGTTTTAGAAAAATGGCATTCCC
    10021          10030     10040     10050     10060     10070
    10021 TGGTGTTTGGAGATAGTGGAGTCGACAAAACGTCTCACCAAAATCTTTTTACCGTAAGGG
                                                         <<<26_R<<< 10133 to
10154
    10081 ATCTGGTAAAGTTGAGGGTTGTATGGTACAAGTAACTTGTGGTACAACTACACTTAACGG
    10081          10090     10100     10110     10120     10130
    10081 TAGACCATTTCAACTCCCAACATACCATGTTCATTGAACACCATGTTGATGTGAATTGCC
    10141 TCTTTGGCTTGATGACGTAGTTTACTGTCCAAGACATGTGATCTGCACCTCTGAAGACAT
    10141          10150     10160     10170     10180     10190
    10141 AGAAACCGAACTACTGCATCAAATGACAGGTTCTGTACACTAGACGTGGAGACTTCTGTA
    10201 GCTTAACCCTAATTATGAAGATTTACTCATTCGTAAGTCTAATCATAATTTCTTGGTACA
    10201          10210     10220     10230     10240     10250
    10201 CGAATTGGGATTAATACTTCTAAATGAGTAAGCATTCAGATTAGTATTAAAGAACCATGT
    10261 GGCTGGTAATGTTCAACTCAGGGTTATTGGACATTCTATGCAAAATTGTGTACTTAAGCT
    10261          10270     10280     10290     10300     10310
    10261 CCGACCATTACAAGTTGAGTCCCAATAACCTGTAAGATACGTTTTAACACATGAATTCGA
    10321 TAAGGTTGATACAGCCAATCCTAAGACACCTAAGTATAAGTTTGTTCGCATTCAACCAGG
    10321          10330     10340     10350     10360     10370
    10321 ATTCCAACTATGTCGGTTAGGATTCTGTGGATTCATATTCAAACAAGCGTAAGTTGGTCC
    10381 ACAGACTTTTTCAGTGTTAGCTTGTTACAATGGTTCACCATCTGGTGTTTACCAATGTGC
    10381          10390     10400     10410     10420     10430
    10381 TGTCTGAAAAAGTCACAATCGAACAATGTTACCAAGTGGTAGACCACAAATGGTTACACG
    10441 TATGAGGCCCAATTTCACTATTAAGGGTTCATTCCTTAATGGTTCATGTGGTAGTGTTGG
    10441          10450     10460     10470     10480     10490
    10441 ATACTCCGGGTTAAAGTGATAATTCCCAAGTAAGGAATTACCAAGTACACCATCACAACC
    10501 TTTTAACATAGATTATGACTGTGTCTCTTTTTGTTACATGCACCATATGGAATTACCAAC
    10501          10510     10520     10530     10540     10550
    10501 AAAATTGTATCTAATACTGACACAGAGAAAAACAATGTACGTGGTATACCTTAATGGTTG
    10561 TGGAGTTCATGCTGGCACAGACTTAGAAGGTAACTTTTATGGACCTTTTGTTGACAGGCA
    10561          10570     10580     10590     10600     10610
    10561 ACCTCAAGTACGACCGTGTCTGAATCTTCCATTGAAAATACCTGGAAAACAACTGTCCGT
    10621 AACAGCACAAGCAGCTGGTACGGACACAACTATTACAGTTAATGTTTTAGCTTGGTTGTA
    10621          10630     10640     10650     10660     10670
    10621 TTGTCGTGTTCGTCGACCATGCCTGTGTTGATAATGTCAATTACAAAATCGAACCAACAT
    10681 CGCTGCTGTTATAAATGGAGACAGGTGGTTTCTCAATCGATTTACCACAACTCTTAATGA
    10681          10690     10700     10710     10720     10730
    10681 GCGACGACAATATTTACCTCTGTCCACCAAAGAGTTAGCTAAATGGTGTTGAGAATTACT
    10741 CTTTAACCTTGTGGCTATGAAGTACAATTATGAACCTCTAACACAAGACCATGTTGACAT
    10741          10750     10760     10770     10780     10790
    10741 GAAATTGGAACACCGATACTTCATGTTAATACTTGGAGATTGTGTTCTGGTACAACTGTA
                      >>>27_F>>> 10812 to 10833
    10801 ACTAGGACCTCTTTCTGCTCAAACTGGAATTGCCGTTTTAGATATGTGTGCTTCATTAAA
    10801          10810     10820     10830     10840     10850
    10801 TGATCCTGGAGAAAGACGAGTTTGACCTTAACGGCAAAATCTATACACACGAAGTAATTT
    10861 AGAATTACTGCAAAATGGTATGAATGGACGTACCATATTGGGTAGTGCTTTATTAGAAGA
    10861          10870     10880     10890     10900     10910
    10861 TCTTAATGACGTTTTACCATACTTACCTGCATGGTATAACCCATCACGAAATAATCTTCT
                                          <<<28_R<<< 10949 to 10970
    10921 TGAATTTACACCTTTTGATGTTGTTAGACAATGCTCAGGTGTTACTTTCCAAAGTGCAGT
    10921          10930     10940     10950     10960     10970
    10921 ACTTAAATGTGGAAAACTACAACAATCTGTTACGAGTCCACAATGAAAGGTTTCACGTCA
    10981 GAAAAGAACAATCAAGGGTACACACCACTGGTTGTTACTCACAATTTTGACTTCACTTTT
    10981          10990     11000     11010     11020     11030
    10981 CTTTTCTTGTTAGTTCCCATGTGTGGTGACCAACAATGAGTGTTAAAACTGAAGTGAAAA
    11041 AGTTTTAGTCCAGAGTACTCAATGGTCTTTGTTCTTTTTTTTGTATGAAAATGCCTTTTT
```

```
11041          11050     11060     11070     11080     11090
11041 TCAAAATCAGGTCTCATGAGTTACCAGAAACAAGAAAAAAAACATACTTTTACGGAAAAA
11101 ACCTTTTGCTATGGGTATTATTGCTATGTCTGCTTTTGCAATGATGTTTGTCAAACATAA
11101          11110     11120     11130     11140     11150
11101 TGGAAAACGATACCCATAATAACGATACAGACGAAAACGTTACTACAAACAGTTTGTATT
11161 GCATGCATTTCTCTGTTTGTTTTTGTTACCTTCTCTTGCCACTGTAGCTTATTTTAATAT
11161          11170     11180     11190     11200     11210
11161 CGTACGTAAAGAGACAAACAAAAACAATGGAAGAGAACGGTGACATCGAATAAAATTATA
11221 GGTCTATATGCCTGCTAGTTGGGTGATGCGTATTATGACATGGTTGGATATGGTTGATAC
11221          11230     11240     11250     11260     11270
11221 CCAGATATACGGACGATCAACCCACTACGCATAATACTGTACCAACCTATACCAACTATG
11281 TAGTTTGTCTGGTTTTAAGCTAAAAGACTGTGTTATGTATGCATCAGCTGTAGTGTTACT
11281          11290     11300     11310     11320     11330
11281 ATCAAACAGACCAAAATTCGATTTTCTGACACAATACATACGTAGTCGACATCACAATGA
11341 AATCCTTATGACAGCAAGAACTGTGTATGATGATGGTGCTAGGAGAGTGTGGACACTTAT
11341          11350     11360     11370     11380     11390
11341 TTAGGAATACTGTCGTTCTTGACACATACTACTACCACGATCCTCTCACACCTGTGAATA
11401 GAATGTCTTGACACTCGTTTATAAAGTTTATTATGGTAATGCTTTAGATCAAGCCATTTC
11401          11410     11420     11430     11440     11450
11401 CTTACAGAACTGTGAGCAAATATTTCAAATAATACCATTACGAAATCTAGTTCGGTAAAG
11461 CATGTGGGCTCTTATAATCTCTGTTACTTCTAACTACTCAGGTGTAGTTACAACTGTCAT
11461          11470     11480     11490     11500     11510
11461 GTACACCCGAGAATATTAGAGACAATGAAGATTGATGAGTCCACATCAATGTTGACAGTA
11521 GTTTTTGGCCAGAGGTATTGTTTTTATGTGTGTTGAGTATTGCCCTATTTTCTTCATAAC
11521          11530     11540     11550     11560     11570
11521 CAAAAACCGGTCTCCATAACAAAAATACACACAACTCATAACGGGATAAAAGAAGTATTG
11581 TGGTAATACACTTCAGTGTATAATGCTAGTTTATTGTTTCTTAGGCTATTTTTGTACTTG
11581          11590     11600     11610     11620     11630
11581 ACCATTATGTGAAGTCACATATTACGATCAAATAACAAAGAATCCGATAAAAACATGAAC
                                >>>29_F>>> 11664 to 11685
11641 TTACTTTGGCCTCTTTTGTTTACTCAACCGCTACTTTAGACTGACTCTTGGTGTTTATGA
11641          11650     11660     11670     11680     11690
11641 AATGAAACCGGAGAAAACAAATGAGTTGGCGATGAAATCTGACTGAGAACCACAAATACT
11701 TTACTTAGTTTCTACACAGGAGTTTAGATATATGAATTCACAGGGACTACTCCCACCCAA
11701          11710     11720     11730     11740     11750
11701 AATGAATCAAAGATGTGTCCTCAAATCTATATACTTAAGTGTCCCTGATGAGGGTGGGTT
          <<<30_R<<< 11767 to 11789
11761 GAATAGCATAGATGCCTTCAAACTCAACATTAAATTGTTGGGTGTTGGTGGCAAACCTTG
11761          11770     11780     11790     11800     11810
11761 CTTATCGTATCTACGGAAGTTTGAGTTGTAATTTAACAACCCACAACCACCGTTTGGAAC
11821 TATCAAAGTAGCCACTGTACAGTCTAAAATGTCAGATGTAAAGTGCACATCAGTAGTCTT
11821          11830     11840     11850     11860     11870
11821 ATAGTTTCATCGGTGACATGTCAGATTTTACAGTCTACATTTCACGTGTAGTCATCAGAA
11881 ACTCTCAGTTTTGCAACAACTCAGAGTAGAATCATCATCTAAATTGTGGGCTCAATGTGT
11881          11890     11900     11910     11920     11930
11881 TGAGAGTCAAAACGTTGTTGAGTCTCATCTTAGTAGTAGATTTAACACCCGAGTTACACA
11941 CCAGTTACACAATGACATTCTCTTAGCTAAAGATACTACTGAAGCCTTTGAAAAAATGGT
11941          11950     11960     11970     11980     11990
11941 GGTCAATGTGTTACTGTAAGAGAATCGATTTCTATGATGACTTCGGAAACTTTTTTACCA
12001 TTCACTACTTTCTGTTTTGCTTTCCATGCAGGGTGCTGTAGACATAAACAAGCTTTGTGA
12001          12010     12020     12030     12040     12050
12001 AAGTGATGAAAGACAAAACGAAAGGTACGTCCCACGACATCTGTATTTGTTCGAAACACT
12061 AGAAATGCTGGACAACAGGGCAACCTTACAAGCTATAGCCTCAGAGTTTAGTTCCCTTCC
12061          12070     12080     12090     12100     12110
12061 TCTTTACGACCTGTTGTCCCGTTGGAATGTTCGATATCGGAGTCTCAAATCAAGGGAAGG
12121 ATCATATGCAGCTTTTGCTACTGCTCAAGAAGCTTATGAGCAGGCTGTTGCTAATGGTGA
12121          12130     12140     12150     12160     12170
12121 TAGTATACGTCGAAAACGATGACGAGTTCTTCGAATACTCGTCCGACAACGATTACCACT
12181 TTCTGAAGTTGTTCTTAAAAAGTTGAAGAAGTCTTTGAATGTGGCTAAATCTGAATTTGA
```

```
12181          12190      12200      12210      12220      12230
12181 AAGACTTCAACAAGAATTTTTCAACTTCTTCAGAAACTTACACCGATTTAGACTTAAACT
12241 CCGTGATGCAGCCATGCAACGTAAGTTGGAAAAGATGGCTGATCAAGCTATGACCCAAAT
12241          12250      12260      12270      12280      12290
12241 GGCACTACGTCGGTACGTTGCATTCAACCTTTTCTACCGACTAGTTCGATACTGGGTTTA
12301 GTATAAACAGGCTAGATCTGAGGACAAGAGGGCAAAAGTTACTAGTGCTATGCAGACAAT
12301          12310      12320      12330      12340      12350
12301 CATATTTGTCCGATCTAGACTCCTGTTCTCCCGTTTTCAATGATCACGATACGTCTGTTA
12361 GCTTTTCACTATGCTTAGAAAGTTGGATAATGATGCACTCAACAACATTATCAACAATGC
12361          12370      12380      12390      12400      12410
12361 CGAAAAGTGATACGAATCTTTCAACCTATTACTACGTGAGTTGTTGTAATAGTTGTTACG
                                >>>31_F>>> 12448 to 12469
12421 AAGAGATGGTTGTGTTCCCTTGAACATAATACCTCTTACAACAGCAGCCAAACTAATGGT
12421          12430      12440      12450      12460      12470
12421 TTCTCTACCAACACAAGGGAACTTGTATTATGGAGAATGTTGTCGTCGGTTTGATTACCA
12481 TGTCATACCAGACTATAACACATATAAAAATACGTGTGATGGTACAACATTTACTTATGC
12481          12490      12500      12510      12520      12530
12481 ACAGTATGGTCTGATATTGTGTATATTTTTATGCACACTACCATGTTGTAAATGAATACG
12541 ATCAGCATTGTGGGAAATCCAACAGGTTGTAGATGCAGATAGTAAAATTGTTCAACTTAG
12541          12550      12560      12570      12580      12590
12541 TAGTCGTAACACCCTTTAGGTTGTCCAACATCTACGTCTATCATTTTAACAAGTTGAATC
12601 TGAAATTAGTATGGACAATTCACCTAATTTAGCATGGCCTCTTATTGTAACAGCTTTAAG
12601          12610      12620      12630      12640      12650
12601 ACTTTAATCATACCTGTTAAGTGGATTAAATCGTACCGGAGAATAACATTGTCGAAATTC
      <<<32_R<<< 12661 to 12682
12661 GGCCAATTCTGCTGTCAAATTACAGAATAATGAGCTTAGTCCTGTTGCACTACGACAGAT
12661          12670      12680      12690      12700      12710
12661 CCGGTTAAGACGACAGTTTAATGTCTTATTACTCGAATCAGGACAACGTGATGCTGTCTA
12721 GTCTTGTGCTGCCGGTACTACACAAACTGCTTGCACTGATGACAATGCGTTAGCTTACTA
12721          12730      12740      12750      12760      12770
12721 CAGAACACGACGGCCATGATGTGTTTGACGAACGTGACTACTGTTACGCAATCGAATGAT
12781 CAACACAACAAAGGGAGGTAGGTTTGTACTTGCACTGTTATCCGATTTACAGGATTTGAA
12781          12790      12800      12810      12820      12830
12781 GTTGTGTTGTTTCCCTCCATCCAAACATGAACGTGACAATAGGCTAAATGTCCTAAACTT
12841 ATGGGCTAGATTCCCTAAGAGTGATGGAACTGGTACTATCTATACAGAACTGGAACCACC
12841          12850      12860      12870      12880      12890
12841 TACCCGATCTAAGGGATTCTCACTACCTTGACCATGATAGATATGTCTTGACCTTGGTGG
12901 TTGTAGGTTTGTTACAGACACACCTAAAGGTCCTAAAGTGAAGTATTTATACTTTATTAA
12901          12910      12920      12930      12940      12950
12901 AACATCCAAACAATGTCTGTGTGGATTTCCAGGATTTCACTTCATAAATATGAAATAATT
12961 AGGATTAAACAACCTAAATAGAGGTATGGTACTTGGTAGTTTAGCTGCCACAGTACGTCT
12961          12970      12980      12990      13000      13010
12961 TCCTAATTTGTTGGATTTATCTCCATACCATGAACCATCAAATCGACGGTGTCATGCAGA
13021 ACAAGCTGGTAATGCAACAGAAGTGCCTGCCAATTCAACTGTATTATCTTTCTGTGCTTT
13021          13030      13040      13050      13060      13070
13021 TGTTCGACCATTACGTTGTCTTCACGGACGGTTAAGTTGACATAATAGAAAGACACGAAA
13081 TGCTGTAGATGCTGCTAAAGCTTACAAAGATTATCTAGCTAGTGGGGGACAACCAATCAC
13081          13090      13100      13110      13120      13130
13081 ACGACATCTACGACGATTTCGAATGTTTCTAATAGATCGATCACCCCCTGTTGGTTAGTG
13141 TAATTGTGTTAAGATGTTGTGTACACACACTGGTACTGGTCAGGCAATAACAGTTACACC
13141          13150      13160      13170      13180      13190
13141 ATTAACACAATTCTACAACACATGTGTGTGACCATGACCAGTCCGTTATTGTCAATGTGG
13201 GGAAGCCAATATGGATCAAGAATCCTTTGGTGGTGCATCGTGTTGTCTGTACTGCCGTTG
13201          13210      13220      13230      13240      13250
13201 CCTTCGGTTATACCTAGTTCTTAGGAAACCACCACGTAGCACAACAGACATGACGGCAAC
13261 CCACATAGATCATCCAAATCCTAAAGGATTTTGTGACTTAAAAGGTAAGTATGTACAAAT
13261          13270      13280      13290      13300      13310
13261 GGTGTATCTAGTAGGTTTAGGATTTCCTAAAACACTGAATTTTCCATTCATACATGTTTA
            >>>33_F>>> 13327 to 13348
```

13321 ACCTACAACTTGTGCTAATGACCCTGTGGGTTTTACACTTAAAAACACAGTCTGTACCGT
13321 13330 13340 13350 13360 13370
13321 TGGATGTTGAACACGATTACTGGGACACCCAAAATGTGAATTTTTGTGTCAGACATGGCA
13381 CTGCGGTATGTGGAAAGGTTATGGCTGTAGTTGTGATCAACTCCGCGAACCCATGCTTCA
13381 13390 13400 13410 13420 13430
13381 GACGCCATACACCTTTCCAATACCGACATCAACACTAGTTGAGGCGCTTGGGTACGAAGT
<<<34_R<<< 13478 to 13498
13441 GTCAGCTGATGCACAATCGTTTTTAAACGGGTTTGCGGTGTAAGTGCAGCCCGTCTTACA
13441 13450 13460 13470 13480 13490
13441 CAGTCGACTACGTGTTAGCAAAAATTTGCCCAAACGCCACATTCACGTCGGGCAGAATGT
13501 CCGTGCGGCACAGGCACTAGTACTGATGTCGTATACAGGGCTTTTGACATCTACAATGAT
13501 13510 13520 13530 13540 13550
13501 GGCACGCCGTGTCCGTGATCATGACTACAGCATATGTCCCGAAAACTGTAGATGTTACTA
13561 AAAGTAGCTGGTTTTGCTAAATTCCTAAAAACTAATTGTTGTCGCTTCCAAGAAAAGGAC
13561 13570 13580 13590 13600 13610
13561 TTTCATCGACCAAAACGATTTAAGGATTTTTGATTAACAACAGCGAAGGTTCTTTTCCTG
13621 GAAGATGACAATTTAATTGATTCTTACTTTGTAGTTAAGAGACACACTTTCTCTAACTAC
13621 13630 13640 13650 13660 13670
13621 CTTCTACTGTTAAATTAACTAAGAATGAAACATCAATTCTCTGTGTGAAAGAGATTGATG
13681 CAACATGAAGAAACAATTTATAATTTACTTAAGGATTGTCCAGCTGTTGCTAAACATGAC
13681 13690 13700 13710 13720 13730
13681 GTTGTACTTCTTTGTTAAATATTAAATGAATTCCTAACAGGTCGACAACGATTTGTACTG
13741 TTCTTTAAGTTTAGAATAGACGGTGACATGGTACCACATATATCACGTCAACGTCTTACT
13741 13750 13760 13770 13780 13790
13741 AAGAAATTCAAATCTTATCTGCCACTGTACCATGGTGTATATAGTGCAGTTGCAGAATGA
13801 AAATACACAATGGCAGACCTCGTCTATGCTTTAAGGCATTTTGATGAAGGTAATTGTGAC
13801 13810 13820 13830 13840 13850
13801 TTTATGTGTTACCGTCTGGAGCAGATACGAAATTCCGTAAAACTACTTCCATTAACACTG
13861 ACATTAAAAGAAATACTTGTCACATACAATTGTTGTGATGATGATTATTTCAATAAAAAG
13861 13870 13880 13890 13900 13910
13861 TGTAATTTTCTTTATGAACAGTGTATGTTAACAACACTACTACTAATAAAGTTATTTTTC
13921 GACTGGTATGATTTTGTAGAAAACCCAGATATATTACGCGTATACGCCAACTTAGGTGAA
13921 13930 13940 13950 13960 13970
13921 CTGACCATACTAAAACATCTTTTGGGTCTATATAATGCGCATATGCGGTTGAATCCACTT
13981 CGTGTACGCCAAGCTTTGTTAAAAACAGTACAATTCTGTGATGCCATGCGAAATGCTGGT
13981 13990 14000 14010 14020 14030
13981 GCACATGCGGTTCGAAACAATTTTTGTCATGTTAAGACACTACGGTACGCTTTACGACCA
14041 ATTGTTGGTGTACTGACATTAGATAATCAAGATCTCAATGGTAACTGGTATGATTTCGGT
14041 14050 14060 14070 14080 14090
14041 TAACAACCACATGACTGTAATCTATTAGTTCTAGAGTTACCATTGACCATACTAAAGCCA
14101 GATTTCATACAAACCACGCCAGGTAGTGGAGTTCCTGTTGTAGATTCTTATTATTCATTG
14101 14110 14120 14130 14140 14150
14101 CTAAAGTATGTTTGGTGCGGTCCATCACCTCAAGGACAACATCTAAGAATAATAAGTAAC
>>>35_F>>> 14210 to 14231
14161 TTAATGCCTATATTAACCTTGACCAGGGCTTTAACTGCAGAGTCACATGTTGACACTGAC
14161 14170 14180 14190 14200 14210
14161 AATTACGGATATAATTGGAACTGGTCCCGAAATTGACGTCTCAGTGTACAACTGTGACTG
14221 TTAACAAAGCCTTACATTAAGTGGGATTTGTTAAAATATGACTTCACGGAAGAGAGGTTA
14221 14230 14240 14250 14260 14270
14221 AATTGTTTCGGAATGTAATTCACCCTAAACAATTTTATACTGAAGTGCCTTCTCTCCAAT
<<<36_R<<< 14310 to 14331
14281 AAACTCTTTGACCGTTATTTTAAATATTGGGATCAGACATACCACCCAAATTGTGTTAAC
14281 14290 14300 14310 14320 14330
14281 TTTGAGAAACTGGCAATAAAATTTATAACCCTAGTCTGTATGGTGGGTTTAACACAATTG
14341 TGTTTGGATGACAGATGCATTCTGCATTGTGCAAACTTTAATGTTTTATTCTCTACAGTG
14341 14350 14360 14370 14380 14390
14341 ACAAACCTACTGTCTACGTAAGACGTAACACGTTTGAAATTACAAAATAAGAGATGTCAC
14401 TTCCCACCTACAAGTTTTGGACCACTAGTGAGAAAAATATTTGTTGATGGTGTTCCATTT
14401 14410 14420 14430 14440 14450

```
14401 AAGGGTGGATGTTCAAAACCTGGTGATCACTCTTTTTATAAACAACTACCACAAGGTAAA
14461 GTAGTTTCAACTGGATACCACTTCAGAGAGCTAGGTGTTGTACATAATCAGGATGTAAAC
14461          14470     14480     14490     14500     14510
14461 CATCAAAGTTGACCTATGGTGAAGTCTCTCGATCCACAACATGTATTAGTCCTACATTTG
14521 TTACATAGCTCTAGACTTAGTTTTAAGGAATTACTTGTGTATGCTGCTGACCCTGCTATG
14521          14530     14540     14550     14560     14570
14521 AATGTATCGAGATCTGAATCAAAATTCCTTAATGAACACATACGACGACTGGGACGATAC
14581 CACGCTGCTTCTGGTAATCTATTACTAGATAAACGCACTACGTGCTTTTCAGTAGCTGCA
14581          14590     14600     14610     14620     14630
14581 GTGCGACGAAGACCATTAGATAATGATCTATTTGCGTGATGCACGAAAAGTCATCGACGT
14641 CTTACTAACAATGTTGCTTTTCAAACTGTCAAACCCGGTAATTTTAACAAAGACTTCTAT
14641          14650     14660     14670     14680     14690
14641 GAATGATTGTTACAACGAAAAGTTTGACAGTTTGGGCCATTAAAATTGTTTCTGAAGATA
14701 GACTTTGCTGTGTCTAAGGGTTTCTTTAAGGAAGGAAGTTCTGTTGAATTAAAACACTTC
14701          14710     14720     14730     14740     14750
14701 CTGAAACGACACAGATTCCCAAAGAAATTCCTTCCTTCAAGACAACTTAATTTTGTGAAG
14761 TTCTTTGCTCAGGATGGTAATGCTGCTATCAGCGATTATGACTACTATCGTTATAATCTA
14761          14770     14780     14790     14800     14810
14761 AAGAAACGAGTCCTACCATTACGACGATAGTCGCTAATACTGATGATAGCAATATTAGAT
14821 CCAACAATGTGTGATATCAGACAACTACTATTTGTAGTTGAAGTTGTTGATAAGTACTTT
14821          14830     14840     14850     14860     14870
14821 GGTTGTTACACACTATAGTCTGTTGATGATAAACATCAACTTCAACAACTATTCATGAAA
14881 GATTGTTACGATGGTGGCTGTATTAATGCTAACCAAGTCATCGTCAACAACCTAGACAAA
14881          14890     14900     14910     14920     14930
14881 CTAACAATGCTACCACCGACATAATTACGATTGGTTCAGTAGCAGTTGTTGGATCTGTTT
14941 TCAGCTGGTTTTCCATTTAATAAATGGGGTAAGGCTAGACTTTATTATGATTCAATGAGT
14941          14950     14960     14970     14980     14990
14941 AGTCGACCAAAAGGTAAATTATTTACCCCATTCCGATCTGAAATAATACTAAGTTACTCA
                                                >>>37_F>>> 15036 to 15061
15001 TATGAGGATCAAGATGCACTTTTCGCATATACAAAACGTAATGTCATCCCTACTATAACT
15001          15010     15020     15030     15040     15050
15001 ATACTCCTAGTTCTACGTGAAAAGCGTATATGTTTTGCATTACAGTAGGGATGATATTGA
15061 CAAATGAATCTTAAGTATGCCATTAGTGCAAAGAATAGAGCTCGCACCGTAGCTGGTGTC
15061          15070     15080     15090     15100     15110
15061 GTTTACTTAGAATTCATACGGTAATCACGTTTCTTATCTCGAGCGTGGCATCGACCACAG
15121 TCTATCTGTAGTACTATGACCAATAGACAGTTTCATCAAAAATTATTGAAATCAATAGCC
15121          15130     15140     15150     15160     15170
15121 AGATAGACATCATGATACTGGTTATCTGTCAAAGTAGTTTTTAATAACTTTAGTTATCGG
                        <<<38_R<<< 15197 to 15219
15181 GCCACTAGAGGAGCTACTGTAGTAATTGGAACAAGCAAATTCTATGGTGGTTGGCACAAC
15181          15190     15200     15210     15220     15230
15181 CGGTGATCTCCTCGATGACATCATTAACCTTGTTCGTTTAAGATACCACCAACCGTGTTG
15241 ATGTTAAAAACTGTTTATAGTGATGTAGAAAACCCTCACCTTATGGGTTGGGATTATCCT
15241          15250     15260     15270     15280     15290
15241 TACAATTTTTGACAAATATCACTACATCTTTTGGGAGTGGAATACCCAACCCTAATAGGA
15301 AAATGTGATAGAGCCATGCCTAACATGCTTAGAATTATGGCCTCACTTGTTCTTGCTCGC
15301          15310     15320     15330     15340     15350
15301 TTTACACTATCTCGGTACGGATTGTACGAATCTTAATACCGGAGTGAACAAGAACGAGCG
15361 AAACATACAACGTGTTGTAGCTTGTCACACCGTTTCTATAGATTAGCTAATGAGTGTGCT
15361          15370     15380     15390     15400     15410
15361 TTTGTATGTTGCACAACATCGAACAGTGTGGCAAAGATATCTAATCGATTACTCACACGA
15421 CAAGTATTGAGTGAAATGGTCATGTGTGGCGGTTCACTATATGTTAAACCAGGTGGAACC
15421          15430     15440     15450     15460     15470
15421 GTTCATAACTCACTTTACCAGTACACACCGCCAAGTGATATACAATTTGGTCCACCTTGG
15481 TCATCAGGAGATGCCACAACTGCTTATGCTAATAGTGTTTTTAACATTTGTCAAGCTGTC
15481          15490     15500     15510     15520     15530
15481 AGTAGTCCTCTACGGTGTTGACGAATACGATTATCACAAAAATTGTAAACAGTTCGACAG
15541 ACGGCCAATGTTAATGCACTTTTATCTACTGATGGTAACAAAATTGCCGATAAGTATGTC
15541          15550     15560     15570     15580     15590
```

```
15541 TGCCGGTTACAATTACGTGAAAATAGATGACTACCATTGTTTTAACGGCTATTCATACAG
15601 CGCAATTTACAACACAGACTTTATGAGTGTCTCTATAGAAATAGAGATGTTGACACAGAC
15601          15610     15620     15630     15640     15650
15601 GCGTTAAATGTTGTGTCTGAAATACTCACAGAGATATCTTTATCTCTACAACTGTGTCTG
15661 TTTGTGAATGAGTTTTACGCATATTTGCGTAAACATTTCTCAATGATGATACTCTCTGAC
15661          15670     15680     15690     15700     15710
15661 AAACACTTACTCAAAATGCGTATAAACGCATTTGTAAAGAGTTACTACTATGAGAGACTG
15721 GATGCTGTTGTGTGTTTCAATAGCACTTATGCATCTCAAGGTCTAGTGGCTAGCATAAAG
15721          15730     15740     15750     15760     15770
15721 CTACGACAACACACAAAGTTATCGTGAATACGTAGAGTTCCAGATCACCGATCGTATTTC
15781 AACTTTAAGTCAGTTCTTTATTATCAAAACAATGTTTTTATGTCTGAAGCAAAATGTTGG
15781          15790     15800     15810     15820     15830
15781 TTGAAATTCAGTCAAGAAATAATAGTTTTGTTACAAAAATACAGACTTCGTTTTACAACC
15841 ACTGAGACTGACCTTACTAAAGGACCTCATGAATTTTGCTCTCAACATACAATGCTAGTT
15841          15850     15860     15870     15880     15890
15841 TGACTCTGACTGGAATGATTTCCTGGAGTACTTAAAACGAGAGTTGTATGTTACGATCAA
                       >>>39_F>>> 15919 to 15940
15901 AAACAGGGTGATGATTATGTGTACCTTCCTTACCCAGATCCATCAAGAATCCTAGGGGCC
15901          15910     15920     15930     15940     15950
15901 TTTGTCCCACTACTAATACACATGGAAGGAATGGGTCTAGGTAGTTCTTAGGATCCCCGG
15961 GGCTGTTTTGTAGATGATATCGTAAAAACAGATGGTACACTTATGATTGAACGGTTCGTG
15961          15970     15980     15990     16000     16010
15961 CCGACAAAACATCTACTATAGCATTTTTGTCTACCATGTGAATACTAACTTGCCAAGCAC
                                                <<<40_R<<< 16063 to 16085
16021 TCTTTAGCTATAGATGCTTACCCACTTACTAAACATCCTAATCAGGAGTATGCTGATGTC
16021          16030     16040     16050     16060     16070
16021 AGAAATCGATATCTACGAATGGGTGAATGATTTGTAGGATTAGTCCTCATACGACTACAG
16081 TTTCATTTGTACTTACAATACATAAGAAAGCTACATGATGAGTTAACAGGACACATGTTA
16081          16090     16100     16110     16120     16130
16081 AAAGTAAACATGAATGTTATGTATTCTTTCGATGTACTACTCAATTGTCCTGTGTACAAT
16141 GACATGTATTCTGTTATGCTTACTAATGATAACACTTCAAGGTATTGGGAACCTGAGTTT
16141          16150     16160     16170     16180     16190
16141 CTGTACATAAGACAATACGAATGATTACTATTGTGAAGTTCCATAACCCTTGGACTCAAA
16201 TATGAGGCTATGTACACACCGCATACAGTCTTACAGGCTGTTGGGGCTTGTGTTCTTTGC
16201          16210     16220     16230     16240     16250
16201 ATACTCCGATACATGTGTGGCGTATGTCAGAATGTCCGACAACCCCGAACACAAGAAACG
16261 AATTCACAGACTTCATTAAGATGTGGTGCTTGCATACGTAGACCATTCTTATGTTGTAAA
16261          16270     16280     16290     16300     16310
16261 TTAAGTGTCTGAAGTAATTCTACACCACGAACGTATGCATCTGGTAAGAATACAACATTT
16321 TGCTGTTACGACCATGTCATATCAACATCACATAAATTAGTCTTGTCTGTTAATCCGTAT
16321          16330     16340     16350     16360     16370
16321 ACGACAATGCTGGTACAGTATAGTTGTAGTGTATTTAATCAGAACAGACAATTAGGCATA
16381 GTTTGCAATGCTCCAGGTTGTGATGTCACAGATGTGACTCAACTTTACTTAGGAGGTATG
16381          16390     16400     16410     16420     16430
16381 CAAACGTTACGAGGTCCAACACTACAGTGTCTACACTGAGTTGAAATGAATCCTCCATAC
16441 AGCTATTATTGTAAATCACATAAACCACCCATTAGTTTTCCATTGTGTGCTAATGGACAA
16441          16450     16460     16470     16480     16490
16441 TCGATAATAACATTTAGTGTATTTGGTGGGTAATCAAAAGGTAACACACGATTACCTGTT
16501 GTTTTTGGTTTATATAAAAATACATGTGTTGGTAGCGATAATGTTACTGACTTTAATGCA
16501          16510     16520     16530     16540     16550
16501 CAAAAACCAAATATATTTTTATGTACACAACCATCGCTATTACAATGACTGAAATTACGT
16561 ATTGCAACATGTGACTGGACAAATGCTGGTGATTACATTTAGCTAACACCTGTACTGAA
16561          16570     16580     16590     16600     16610
16561 TAACGTTGTACACTGACCTGTTTACGACCACTAATGTAAAATCGATTGTGGACATGACTT
16621 AGACTCAAGCTTTTTGCAGCAGAAACGCTCAAAGCTACTGAGGAGACATTTAAACTGTCT
16621          16630     16640     16650     16660     16670
16621 TCTGAGTTCGAAAAACGTCGTCTTTGCGAGTTTCGATGACTCCTCTGTAAATTTGACAGA
16681 TATGGTATTGCTACTGTACGTGAAGTGCTGTCTGACAGAGAATTACATCTTTCATGGGAA
16681          16690     16700     16710     16720     16730
```

```
16681 ATACCATAACGATGACATGCACTTCACGACAGACTGTCTCTTAATGTAGAAAGTACCCTT
16741 GTTGGTAAACCTAGACCACCACTTAACCGAAATTATGTCTTTACTGGTTATCGTGTAACT
16741           16750     16760     16770     16780     16790
16741 CAACCATTTGGATCTGGTGGTGAATTGGCTTTAATACAGAAATGACCAATAGCACATTGA
                                                 >>>41_F>>> 16848 to 16870
16801 AAAAACAGTAAAGTACAAATAGGAGAGTACACCTTTGAAAAAGGTGACTATGGTGATGCT
16801           16810     16820     16830     16840     16850
16801 TTTTTGTCATTTCATGTTTATCCTCTCATGTGGAAACTTTTTCCACTGATACCACTACGA
16861 GTTGTTTACCGAGGTACAACAACTTACAAATTAAATGTTGGTGATTATTTTGTGCTGACA
16861           16870     16880     16890     16900     16910
16861 CAACAAATGGCTCCATGTTGTTGAATGTTTAATTTACAACCACTAATAAAACACGACTGT
                                        <<<42_R<<< 16959 to 16980
16921 TCACATACAGTAATGCCATTAAGTGCACCTACACTAGTGCCACAAGAGCACTATGTTAGA
16921           16930     16940     16950     16960     16970
16921 AGTGTATGTCATTACGGTAATTCACGTGGATGTGATCACGGTGTTCTCGTGATACAATCT
16981 ATTACTGGCTTATACCCAACACTCAATATCTCAGATGAGTTTTCTAGCAATGTTGCAAAT
16981           16990     17000     17010     17020     17030
16981 TAATGACCGAATATGGGTTGTGAGTTATAGAGTCTACTCAAAAGATCGTTACAACGTTTA
17041 TATCAAAAGGTTGGTATGCAAAAGTATTCTACACTCCAGGGACCACCTGGTACTGGTAAG
17041           17050     17060     17070     17080     17090
17041 ATAGTTTTCCAACCATACGTTTTCATAAGATGTGAGGTCCCTGGTGGACCATGACCATTC
17101 AGTCATTTTGCTATTGGCCTAGCTCTCTACTACCCTTCTGCTCGCATAGTGTATACAGCT
17101           17110     17120     17130     17140     17150
17101 TCAGTAAAACGATAACCGGATCGAGAGATGATGGGAAGACGAGCGTATCACATATGTCGA
17161 TGCTCTCATGCCGCTGTTGATGCACTATGTGAGAAGGCATTAAAATATTTGCCTATAGAT
17161           17170     17180     17190     17200     17210
17161 ACGAGAGTACGGCGACAACTACGTGATACACTCTTCCGTAATTTTATAAACGGATATCTA
17221 AAATGTAGTAGAATTATACCTGCACGTGCTCGTGTAGAGTGTTTTGATAAATTCAAAGTG
17221           17230     17240     17250     17260     17270
17221 TTTACATCATCTTAATATGGACGTGCACGAGCACATCTCACAAAACTATTTAAGTTTCAC
17281 AATTCAACATTAGAACAGTATGTCTTTTGTACTGTAAATGCATTGCCTGAGACGACAGCA
17281           17290     17300     17310     17320     17330
17281 TTAAGTTGTAATCTTGTCATACAGAAAACATGACATTTACGTAACGGACTCTGCTGTCGT
17341 GATATAGTTGTCTTTGATGAAATTTCAATGGCCACAAATTATGATTTGAGTGTTGTCAAT
17341           17350     17360     17370     17380     17390
17341 CTATATCAACAGAAACTACTTTAAAGTTACCGGTGTTTAATACTAAACTCACAACAGTTA
17401 GCCAGATTACGTGCTAAGCACTATGTGTACATTGGCGACCCTGCTCAATTACCTGCACCA
17401           17410     17420     17430     17440     17450
17401 CGGTCTAATGCACGATTCGTGATACACATGTAACCGCTGGGACGAGTTAATGGACGTGGT
17461 CGCACATTGCTAACTAAGGGCACACTAGAACCAGAATATTTCAATTCAGTGTGTAGACTT
17461           17470     17480     17490     17500     17510
17461 GCGTGTAACGATTGATTCCCGTGTGATCTTGGTCTTATAAAGTTAAGTCACACATCTGAA
17521 ATGAAAACTATAGGTCCAGACATGTTCCTCGGAACTTGTCGGCGTTGTCCTGCTGAAATT
17521           17530     17540     17550     17560     17570
17521 TACTTTTGATATCCAGGTCTGTACAAGGAGCCTTGAACAGCCGCAACAGGACGACTTTAA
17581 GTTGACACTGTGAGTGCTTTGGTTTATGATAATAAGCTTAAAGCACATAAAGACAAATCA
17581           17590     17600     17610     17620     17630
17581 CAACTGTGACACTCACGAAACCAAATACTATTATTCGAATTTCGTGTATTTCTGTTTAGT
17641 GCTCAATGCTTTAAAATGTTTTATAAGGGTGTTATCACGCATGATGTTTCATCTGCAATT
17641           17650     17660     17670     17680     17690
17641 CGAGTTACGAAATTTTACAAAATATTCCCACAATAGTGCGTACTACAAAGTAGACGTTAA
                                   >>>43_F>>> 17729 to 17750
17701 AACAGGCCACAAATAGGCGTGGTAAGAGAATTCCTTACACGTAACCCTGCTTGGAGAAAA
17701           17710     17720     17730     17740     17750
17701 TTGTCCGGTGTTTATCCGCACCATTCTCTTAAGGAATGTGCATTGGGACGAACCTCTTTT
17761 GCTGTCTTTATTTCACCTTATAATTCACAGAATGCTGTAGCCTCAAAGATTTTGGGACTA
17761           17770     17780     17790     17800     17810
17761 CGACAGAAATAAAGTGGAATATTAAGTGTCTTACGACATCGGAGTTTCTAAAACCCTGAT
                        <<<44_R<<< 17836 to 17857
```

```
17821 CCAACTCAAACTGTTGATTCATCACAGGGCTCAGAATATGACTATGTCATATTCACTCAA
17821          17830     17840     17850     17860     17870
17821 GGTTGAGTTTGACAACTAAGTAGTGTCCCGAGTCTTATACTGATACAGTATAAGTGAGTT
17881 ACCACTGAAACAGCTCACTCTTGTAATGTAAACAGATTTAATGTTGCTATTACCAGAGCA
17881          17890     17900     17910     17920     17930
17881 TGGTGACTTTGTCGAGTGAGAACATTACATTTGTCTAAATTACAACGATAATGGTCTCGT
17941 AAAGTAGGCATACTTTGCATAATGTCTGATAGAGACCTTTATGACAAGTTGCAATTTACA
17941          17950     17960     17970     17980     17990
17941 TTTCATCCGTATGAAACGTATTACAGACTATCTCTGGAAATACTGTTCAACGTTAAATGT
18001 AGTCTTGAAATTCCACGTAGGAATGTGGCAACTTTACAAGCTGAAAATGTAACAGGACTC
18001          18010     18020     18030     18040     18050
18001 TCAGAACTTTAAGGTGCATCCTTACACCGTTGAAATGTTCGACTTTTACATTGTCCTGAG
18061 TTTAAAGATTGTAGTAAGGTAATCACTGGGTTACATCCTACACAGGCACCTACACACCTC
18061          18070     18080     18090     18100     18110
18061 AAATTTCTAACATCATTCCATTAGTGACCCAATGTAGGATGTGTCCGTGGATGTGTGGAG
18121 AGTGTTGACACTAAATTCAAAACTGAAGGTTTATGTGTTGACATACCTGGCATACCTAAG
18121          18130     18140     18150     18160     18170
18121 TCACAACTGTGATTTAAGTTTTGACTTCCAAATACACAACTGTATGGACCGTATGGATTC
18181 GACATGACCTATAGAAGACTCATCTCTATGATGGGTTTTAAAATGAATTATCAAGTTAAT
18181          18190     18200     18210     18220     18230
18181 CTGTACTGGATATCTTCTGAGTAGAGATACTACCCAAAATTTTACTTAATAGTTCAATTA
18241 GGTTACCCTAACATGTTTATCACCCGCGAAGAAGCTATAAGACATGTACGTGCATGGATT
18241          18250     18260     18270     18280     18290
18241 CCAATGGGATTGTACAAATAGTGGGCGCTTCTTCGATATTCTGTACATGCACGTACCTAA
18301 GGCTTCGATGTCGAGGGGTGTCATGCTACTAGAGAAGCTGTTGGTACCAATTTACCTTTA
18301          18310     18320     18330     18340     18350
18301 CCGAAGCTACAGCTCCCCACAGTACGATGATCTCTTCGACAACCATGGTTAAATGGAAAT
18361 CAGCTAGGTTTTTCTACAGGTGTTAACCTAGTTGCTGTACCTACAGGTTATGTTGATACA
18361          18370     18380     18390     18400     18410
18361 GTCGATCCAAAAAGATGTCCACAATTGGATCAACGACATGGATGTCCAATACAACTATGT
18421 CCTAATAATACAGATTTTTCCAGAGTTAGTGCTAAACCACCGCCTGGAGATCAATTTAAA
18421          18430     18440     18450     18460     18470
18421 GGATTATTATGTCTAAAAAGGTCTCAATCACGATTTGGTGGCGGACCTCTAGTTAAATTT
                                           >>>45_F>>> 18511 to 18534
18481 CACCTCATACCACTTATGTACAAAGGACTTCCTTGGAATGTAGTGCGTATAAAGATTGTA
18481          18490     18500     18510     18520     18530
18481 GTGGAGTATGGTGAATACATGTTTCCTGAAGGAACCTTACATCACGCATATTTCTAACAT
18541 CAAATGTTAAGTGACACACTTAAAAATCTCTCTGACAGAGTCGTATTTGTCTTATGGGCA
18541          18550     18560     18570     18580     18590
18541 GTTTACAATTCACTGTGTGAATTTTTAGAGAGACTGTCTCAGCATAAACAGAATACCCGT
                                                             <<<46_R<<< 18652 to
18673
18601 CATGGCTTTGAGTTGACATCTATGAAGTATTTTGTGAAAATAGGACCTGAGCGCACCTGT
18601          18610     18620     18630     18640     18650
18601 GTACCGAAACTCAACTGTAGATACTTCATAAAACACTTTTATCCTGGACTCGCGTGGACA
18661 TGTCTATGTGATAGACGTGCCACATGCTTTTCCACTGCTTCAGACACTTATGCCTGTTGG
18661          18670     18680     18690     18700     18710
18661 ACAGATACACTATCTGCACGGTGTACGAAAAGGTGACGAAGTCTGTGAATACGGACAACC
18721 CATCATTCTATTGGATTTGATTACGTCTATAATCCGTTTATGATTGATGTTCAACAATGG
18721          18730     18740     18750     18760     18770
18721 GTAGTAAGATAACCTAAACTAATGCAGATATTAGGCAAATACTAACTACAAGTTGTTACC
18781 GGTTTTACAGGTAACCTACAAAGCAACCATGATCTGTATTGTCAAGTCCATGGTAATGCA
18781          18790     18800     18810     18820     18830
18781 CCAAAATGTCCATTGGATGTTTCGTTGGTACTAGACATAACAGTTCAGGTACCATTACGT
18841 CATGTAGCTAGTTGTGATGCAATCATGACTAGGTGTCTAGCTGTCCACGAGTGCTTTGTT
18841          18850     18860     18870     18880     18890
18841 GTACATCGATCAACACTACGTTAGTACTGATCCACAGATCGACAGGTGCTCACGAAACAA
18901 AAGCGTGTTGACTGGACTATTGAATATCCTATAATTGGTGATGAACTGAAGATTAATGCG
18901          18910     18920     18930     18940     18950
```

```
18901 TTCGCACAACTGACCTGATAACTTATAGGATATTAACCACTACTTGACTTCTAATTACGC
18961 GCTTGTAGAAAGGTTCAACACATGGTTGTTAAAGCTGCATTATTAGCAGACAAATTCCCA
18961          18970     18980     18990     19000     19010
18961 CGAACATCTTTCCAAGTTGTGTACCAACAATTTCGACGTAATAATCGTCTGTTTAAGGGT
19021 GTTCTTCACGACATTGGTAACCCTAAAGCTATTAAGTGTGTACCTCAAGCTGATGTAGAA
19021          19030     19040     19050     19060     19070
19021 CAAGAAGTGCTGTAACCATTGGGATTTCGATAATTCACACATGGAGTTCGACTACATCTT
19081 TGGAAGTTCTATGATGCACAGCCTTGTAGTGACAAAGCTTATAAAATAGAAGAATTATTC
19081          19090     19100     19110     19120     19130
19081 ACCTTCAAGATACTACGTGTCGGAACATCACTGTTTCGAATATTTTATCTTCTTAATAAG
19141 TATTCTTATGCCACACATTCTGACAAATTCACAGATGGTGTATGCCTATTTTGGAATTGC
19141          19150     19160     19170     19180     19190
19141 ATAAGAATACGGTGTGTAAGACTGTTTAAGTGTCTACCACATACGGATAAAACCTTAACG
19201 AATGTCGATAGATATCCTGCTAATTCCATTGTTTGTAGATTTGACACTAGAGTGCTATCT
19201          19210     19220     19230     19240     19250
19201 TTACAGCTATCTATAGGACGATTAAGGTAACAAACATCTAAACTGTGATCTCACGATAGA
         >>>47_F>>> 19264 to 19285
19261 AACCTTAACTTGCCTGGTTGTGATGGTGGCAGTTTGTATGTAAATAAACATGCATTCCAC
19261          19270     19280     19290     19300     19310
19261 TTGGAATTGAACGGACCAACACTACCACCGTCAAACATACATTTATTTGTACGTAAGGTG
19321 ACACCAGCTTTTGATAAAAGTGCTTTTGTTAATTTAAAACAATTACCATTTTTCTATTAC
19321          19330     19340     19350     19360     19370
19321 TGTGGTCGAAAACTATTTTCACGAAAACAATTAAATTTTGTTAATGGTAAAAAGATAATG
19381 TCTGACAGTCCATGTGAGTCTCATGGAAAACAAGTAGTGTCAGATATAGATTATGTACCA
19381          19390     19400     19410     19420     19430
19381 AGACTGTCAGGTACACTCAGAGTACCTTTTGTTCATCACAGTCTATATCTAATACATGGT
                                   <<<48_R<<< 19470 to 19491
19441 CTAAAGTCTGCTACGTGTATAACACGTTGCAATTTAGGTGGTGCTGTCTGTAGACATCAT
19441          19450     19460     19470     19480     19490
19441 GATTTCAGACGATGCACATATTGTGCAACGTTAAATCCACCACGACAGACATCTGTAGTA
19501 GCTAATGAGTACAGATTGTATCTCGATGCTTATAACATGATGATCTCAGCTGGCTTTAGC
19501          19510     19520     19530     19540     19550
19501 CGATTACTCATGTCTAACATAGAGCTACGAATATTGTACTACTAGAGTCGACCGAAATCG
19561 TTGTGGGTTTACAAACAATTTGATACTTATAACCTCTGGAACACTTTTACAAGACTTCAG
19561          19570     19580     19590     19600     19610
19561 AACACCCAAATGTTTGTTAAACTATGAATATTGGAGACCTTGTGAAAATGTTCTGAAGTC
19621 AGTTTAGAAAATGTGGCTTTTAATGTTGTAAATAAGGGACACTTTGATGGACAACAGGGT
19621          19630     19640     19650     19660     19670
19621 TCAAATCTTTTACACCGAAAATTACAACATTTATTCCCTGTGAAACTACCTGTTGTCCCA
19681 GAAGTACCAGTTTCTATCATTAATAACACTGTTTACACAAAAGTTGATGGTGTTGATGTA
19681          19690     19700     19710     19720     19730
19681 CTTCATGGTCAAAGATAGTAATTATTGTGACAAATGTGTTTTCAACTACCACAACTACAT
19741 GAATTGTTTGAAAATAAAACAACATTACCTGTTAATGTAGCATTTGAGCTTTGGGCTAAG
19741          19750     19760     19770     19780     19790
19741 CTTAACAAACTTTTATTTTGTTGTAATGGACAATTACATCGTAAACTCGAAACCCGATTC
19801 CGCAACATTAAACCAGTACCAGAGGTGAAAATACTCAATAATTTGGGTGTGGACATTGCT
19801          19810     19820     19830     19840     19850
19801 GCGTTGTAATTTGGTCATGGTCTCCACTTTTATGAGTTATTAAACCCACACCTGTAACGA
19861 GCTAATACTGTGATCTGGGACTACAAAAGAGATGCTCCAGCACATATATCTACTATTGGT
19861          19870     19880     19890     19900     19910
19861 CGATTATGACACTAGACCCTGATGTTTTCTCTACGAGGTCGTGTATATAGATGATAACCA
19921 GTTTGTTCTATGACTGACATAGCCAAGAAACCAACTGAAACGATTTGTGCACCACTCACT
19921          19930     19940     19950     19960     19970
19921 CAAACAAGATACTGACTGTATCGGTTCTTTGGTTGACTTTGCTAAACACGTGGTGAGTGA
19981 GTCTTTTTTGATGGTAGAGTTGATGGTCAAGTAGACTTATTTAGAAATGCCCGTAATGGT
19981          19990     20000     20010     20020     20030
19981 CAGAAAAAACTACCATCTCAACTACCAGTTCATCTGAATAAATCTTTACGGGCATTACCA
                                                >>>49_F>>> 20081 to 20102
20041 GTTCTTATTACAGAAGGTAGTGTTAAAGGTTTACAACCATCTGTAGGTCCCAAACAAGCT
```

```
20041          20050     20060     20070     20080     20090
20041 CAAGAATAATGTCTTCCATCACAATTTCCAAATGTTGGTAGACATCCAGGGTTTGTTCGA
20101 AGTCTTAATGGAGTCACATTAATTGGAGAAGCCGTAAAAACACAGTTCAATTATTATAAG
20101          20110     20120     20130     20140     20150
20101 TCAGAATTACCTCAGTGTAATTAACCTCTTCGGCATTTTTGTGTCAAGTTAATAATATTC
20161 AAAGTTGATGGTGTTGTCCAACAATTACCTGAAACTTACTTTACTCAGAGTAGAAATTTA
20161          20170     20180     20190     20200     20210
20161 TTTCAACTACCACAACAGGTTGTTAATGGACTTTGAATGAAATGAGTCTCATCTTTAAAT
                 <<<50_R<<< 20233 to 20254
20221 CAAGAATTTAAACCCAGGAGTCAAATGGAAATTGATTTCTTAGAATTAGCTATGGATGAA
20221          20230     20240     20250     20260     20270
20221 GTTCTTAAATTTGGGTCCTCAGTTTACCTTTAACTAAAGAATCTTAATCGATACCTACTT
20281 TTCATTGAACGGTATAAATTAGAAGGCTATGCCTTCGAACATATCGTTTATGGAGATTTT
20281          20290     20300     20310     20320     20330
20281 AAGTAACTTGCCATATTTAATCTTCCGATACGGAAGCTTGTATAGCAAATACCTCTAAAA
20341 AGTCATAGTCAGTTAGGTGGTTTACATCTACTGATTGGACTAGCTAAACGTTTTAAGGAA
20341          20350     20360     20370     20380     20390
20341 TCAGTATCAGTCAATCCACCAAATGTAGATGACTAACCTGATCGATTTGCAAAATTCCTT
20401 TCACCTTTTGAATTAGAAGATTTTATTCCTATGGACAGTACAGTTAAAAACTATTTCATA
20401          20410     20420     20430     20440     20450
20401 AGTGGAAAACTTAATCTTCTAAAATAAGGATACCTGTCATGTCAATTTTTGATAAAGTAT
20461 ACAGATGCGCAAACAGGTTCATCTAAGTGTGTGTGTTCTGTTATTGATTTATTACTTGAT
20461          20470     20480     20490     20500     20510
20461 TGTCTACGCGTTTGTCCAAGTAGATTCACACACACAAGACAATAACTAAATAATGAACTA
20521 GATTTTGTTGAAATAATAAAATCCCAAGATTTATCTGTAGTTTCTAAGGTTGTCAAAGTG
20521          20530     20540     20550     20560     20570
20521 CTAAAACAACTTTATTATTTTAGGGTTCTAAATAGACATCAAAGATTCCAACAGTTTCAC
20581 ACTATTGACTATACAGAAATTTCATTTATGCTTTGGTGTAAAGATGGCCATGTAGAAACA
20581          20590     20600     20610     20620     20630
20581 TGATAACTGATATGTCTTTAAAGTAAATACGAAACCACATTTCTACCGGTACATCTTTGT
20641 TTTTACCCAAAATTACAATCTAGTCAAGCGTGGCAACCGGGTGTTGCTATGCCTAATCTT
20641          20650     20660     20670     20680     20690
20641 AAAATGGGTTTTAATGTTAGATCAGTTCGCACCGTTGGCCCACAACGATACGGATTAGAA
20701 TACAAAATGCAAAGAATGCTATTAGAAAAGTGTGACCTTCAAAATTATGGTGATAGTGCA
20701          20710     20720     20730     20740     20750
20701 ATGTTTTACGTTTCTTACGATAATCTTTTCACACTGGAAGTTTTAATACCACTATCACGT
20761 ACATTACCTAAAGGCATAATGATGAATGTCGCAAAATATACTCAACTGTGTCAATATTTA
20761          20770     20780     20790     20800     20810
20761 TGTAATGGATTTCCGTATTACTACTTACAGCGTTTTATATGAGTTGACACAGTTATAAAT
                 >>>51_F>>> 20833 to 20857
20821 AACACATTAACATTAGCTGTACCCTATAATATGAGAGTTATACATTTTGGTGCTGGTTCT
20821          20830     20840     20850     20860     20870
20821 TTGTGTAATTGTAATCGACATGGGATATTATACTCTCAATATGTAAAACCACGACCAAGA
20881 GATAAAGGAGTTGCACCAGGTACAGCTGTTTTAAGACAGTGGTTGCCTACGGGTACGCTG
20881          20890     20900     20910     20920     20930
20881 CTATTTCCTCAACGTGGTCCATGTCGACAAAATTCTGTCACCAACGGATGCCCATGCGAC
                                                         <<<52_R<<< 20991 to
21012
20941 CTTGTCGATTCAGATCTTAATGACTTTGTCTCTGATGCAGATTCAACTTTGATTGGTGAT
20941          20950     20960     20970     20980     20990
20941 GAACAGCTAAGTCTAGAATTACTGAAACAGAGACTACGTCTAAGTTGAAACTAACCACTA
21001 TGTGCAACTGTACATACAGCTAATAAATGGGATCTCATTATTAGTGATATGTACGACCCT
21001          21010     21020     21030     21040     21050
21001 ACACGTTGACATGTATGTCGATTATTTACCCTAGAGTAATAATCACTATACATGCTGGGA
21061 AAGACTAAAAATGTTACAAAAGAAAATGACTCTAAAGAGGGTTTTTTCACTTACATTTGT
21061          21070     21080     21090     21100     21110
21061 TTCTGATTTTTACAATGTTTTCTTTTACTGAGATTTCTCCCAAAAAAGTGAATGTAAACA
21121 GGGTTTATACAACAAAAGCTAGCTCTTGGAGGTTCCGTGGCTATAAAGATAACAGAACAT
21121          21130     21140     21150     21160     21170
```

```
21121 CCCAAATATGTTGTTTTCGATCGAGAACCTCCAAGGCACCGATATTTCTATTGTCTTGTA
21181 TCTTGGAATGCTGATCTTTATAAGCTCATGGGACACTTCGCATGGTGGACAGCCTTTGTT
21181           21190      21200      21210      21220      21230
21181 AGAACCTTACGACTAGAAATATTCGAGTACCCTGTGAAGCGTACCACCTGTCGGAAACAA
21241 ACTAATGTGAATGCGTCATCATCTGAAGCATTTTTAATTGGATGTAATTATCTTGGCAAA
21241           21250      21260      21270      21280      21290
21241 TGATTACACTTACGCAGTAGTAGACTTCGTAAAAATTAACCTACATTAATAGAACCGTTT
21301 CCACGCGAACAAATAGATGGTTATGTCATGCATGCAAATTACATATTTGGAGGAATACA
21301           21310      21320      21330      21340      21350
21301 GGTGCGCTTGTTTATCTACCAATACAGTACGTACGTTTAATGTATAAAACCTCCTTATGT
21361 AATCCAATTCAGTTGTCTTCCTATTCTTTATTTGACATGAGTAAATTTCCCCTTAAATTA
21361           21370      21380      21390      21400      21410
21361 TTAGGTTAAGTCAACAGAAGGATAAGAAATAAACTGTACTCATTTAAAGGGGAATTTAAT
21421 AGGGGTACTGCTGTTATGTCTTTAAAAGAAGGTCAAATCAATGATATGATTTTATCTCTT
21421           21430      21440      21450      21460      21470
21421 TCCCCATGACGACAATACAGAAATTTTCTTCCAGTTTAGTTACTATACTAAAATAGAGAA
21481 CTTAGTAAAGGTAGACTTATAATTAGAGAAAACAACAGAGTTGTTATTTCTAGTGATGTT
21481           21490      21500      21510      21520      21530
21481 GAATCATTTCCATCTGAATATTAATCTCTTTTGTTGTCTCAACAATAAAGATCACTACAA
21541 CTTGTTAACAACTAAACGAACAATGTTTGTTTTTCTTGTTTTATTGCCACTAGTCTCTAG
21541           21550      21560      21570      21580      21590
21541 GAACAATTGTTGATTTGCTTGTTACAAACAAAAAGAACAAAATAACGGTGATCAGAGATC
                        >>>53_F>>> 21616 to 21637
21601 TCAGTGTGTTAATCTTACAACCAGAACTCAATTACCCCCTGCATACACTAATTCTTTCAC
21601           21610      21620      21630      21640      21650
21601 AGTCACACAATTAGAATGTTGGTCTTGAGTTAATGGGGGACGTATGTGATTAAGAAAGTG
21661 ACGTGGTGTTTATTACCCTGACAAAGTTTTCAGATCCTCAGTTTTACATTCAACTCAGGA
21661           21670      21680      21690      21700      21710
21661 TGCACCACAAATAATGGGACTGTTTCAAAAGTCTAGGAGTCAAAATGTAAGTTGAGTCCT
                              <<<54_R<<< 21742 to 21763
21721 CTTGTTCTTACCTTTCTTTTCCAATGTTACTTGGTTCCATGCTATACATGTCTCTGGGAC
21721           21730      21740      21750      21760      21770
21721 GAACAAGAATGGAAAGAAAAGGTTACAATGAACCAAGGTACGATATGTACAGAGACCCTG
21781 CAATGGTACTAAGAGGTTTGATAACCCTGTCCTACCATTTAATGATGGTGTTTATTTTGC
21781           21790      21800      21810      21820      21830
21781 GTTACCATGATTCTCCAAACTATTGGGACAGGATGGTAAATTACTACCACAAATAAAACG
21841 TTCCACTGAGAAGTCTAACATAATAAGAGGCTGGATTTTTGGTACTACTTTAGATTCGAA
21841           21850      21860      21870      21880      21890
21841 AAGGTGACTCTTCAGATTGTATTATTCTCCGACCTAAAAACCATGATGAAATCTAAGCTT
21901 GACCCAGTCCCTACTTATTGTTAATAACGCTACTAATGTTGTTATTAAAGTCTGTGAATT
21901           21910      21920      21930      21940      21950
21901 CTGGGTCAGGGATGAATAACAATTATTGCGATGATTACAACAATAATTTCAGACACTTAA
21961 TCAATTTTGTAATGATCCATTTTTGGGTGTTTATTACCACAAAAACAACAAAAGTTGGAT
21961           21970      21980      21990      22000      22010
21961 AGTTAAAACATTACTAGGTAAAAACCCACAAATAATGGTGTTTTTGTTGTTTTCAACCTA
22021 GGAAAGTGAGTTCAGAGTTTATTCTAGTGCGAATAATTGCACTTTTGAATATGTCTCTCA
22021           22030      22040      22050      22060      22070
22021 CCTTTCACTCAAGTCTCAAATAAGATCACGCTTATTAACGTGAAAACTTATACAGAGAGT
22081 GCCTTTTCTTATGGACCTTGAAGGAAAACAGGGTAATTTCAAAAATCTTAGGGAATTTGT
22081           22090      22100      22110      22120      22130
22081 CGGAAAAGAATACCTGGAACTTCCTTTTGTCCCATTAAAGTTTTTAGAATCCCTTAAACA
22141 GTTTAAGAATATTGATGGTTATTTTAAAATATATTCTAAGCACACGCCTATTAATTTAGT
22141           22150      22160      22170      22180      22190
22141 CAAATTCTTATAACTACCAATAAAATTTTATATAAGATTCGTGTGCGGATAATTAAATCA
22201 GCGTGATCTCCCTCAGGGTTTTTCGGCTTTAGAACCATTGGTAGATTTGCCAATAGGTAT
22201           22210      22220      22230      22240      22250
22201 CGCACTAGAGGGAGTCCCAAAAAGCCGAAATCTTGGTAACCATCTAAACGGTTATCCATA
22261 TAACATCACTAGGTTTCAAACTTTACTTGCTTTACATAGAAGTTATTTGACTCCTGGTGA
22261           22270      22280      22290      22300      22310
```

```
22261 ATTGTAGTGATCCAAAGTTTGAAATGAACGAAATGTATCTTCAATAAACTGAGGACCACT
22321 TTCTTCTTCAGGTTGGACAGCTGGTGCTGCAGCTTATTATGTGGGTTATCTTCAACCTAG
22321          22330     22340     22350     22360     22370
22321 AAGAAGAAGTCCAACCTGTCGACCACGACGTCGAATAATACACCCAATAGAAGTTGGATC
22381 GACTTTTCTATTAAAATATAATGAAAATGGAACCATTACAGATGCTGTAGACTGTGCACT
22381          22390     22400     22410     22420     22430
22381 CTGAAAAGATAATTTTATATTACTTTTACCTTGGTAATGTCTACGACATCTGACACGTGA
        >>>55_F>>> 22443 to 22464
22441 TGACCCTCTCTCAGAAACAAAGTGTACGTTGAAATCCTTCACTGTAGAAAAAGGAATCTA
22441          22450     22460     22470     22480     22490
22441 ACTGGGAGAGAGTCTTTGTTTCACATGCAACTTTAGGAAGTGACATCTTTTTCCTTAGAT
22501 TCAAACTTCTAACTTTAGAGTCCAACCAACAGAATCTATTGTTAGATTTCCTAATATTAC
22501          22510     22520     22530     22540     22550
22501 AGTTTGAAGATTGAAATCTCAGGTTGGTTGTCTTAGATAACAATCTAAAGGATTATAATG
                                   <<<56_R<<< 22591 to 22609
22561 AAACTTGTGCCCTTTTGGTGAAGTTTTTAACGCCACCAGATTTGCATCTGTTTATGCTTG
22561          22570     22580     22590     22600     22610
22561 TTTGAACACGGGAAAACCACTTCAAAAATTGCGGTGGTCTAAACGTAGACAAATACGAAC
22621 GAACAGGAAGAGAATCAGCAACTGTGTTGCTGATTATTCTGTCCTATATAATTCCGCATC
22621          22630     22640     22650     22660     22670
22621 CTTGTCCTTCTCTTAGTCGTTGACACAACGACTAATAAGACAGGATATATTAAGGCGTAG
22681 ATTTTCCACTTTTAAGTGTTATGGAGTGTCTCCTACTAAATTAAATGATCTCTGCTTTAC
22681          22690     22700     22710     22720     22730
22681 TAAAAGGTGAAAATTCACAATACCTCACAGAGGATGATTTAATTTACTAGAGACGAAATG
22741 TAATGTCTATGCAGATTCATTTGTAATTAGAGGTGATGAAGTCAGACAAATCGCTCCAGG
22741          22750     22760     22770     22780     22790
22741 ATTACAGATACGTCTAAGTAAACATTAATCTCCACTACTTCAGTCTGTTTAGCGAGGTCC
22801 GCAAACTGGAAAGATTGCTGATTATAATTATAAATTACCAGATGATTTTACAGGCTGCGT
22801          22810     22820     22830     22840     22850
22801 CGTTTGACCTTTCTAACGACTAATATTAATATTTAATGGTCTACTAAAATGTCCGACGCA
22861 TATAGCTTGGAATTCTAACAATCTTGATTCTAAGGTTGGTGGTAATTATAATTACCTGTA
22861          22870     22880     22890     22900     22910
22861 ATATCGAACCTTAAGATTGTTAGAACTAAGATTCCAACCACCATTAATATTAATGGACAT
22921 TAGATTGTTTAGGAAGTCTAATCTCAAACCTTTTGAGAGAGATATTTCAACTGAAATCTA
22921          22930     22940     22950     22960     22970
22921 ATCTAACAAATCCTTCAGATTAGAGTTTGGAAAACTCTCTCTATAAAGTTGACTTTAGAT
22981 TCAGGCCGGTAGCACACCTTGTAATGGTGTTGAAGGTTTTAATTGTTACTTTCCTTTACA
22981          22990     23000     23010     23020     23030
22981 AGTCCGGCCATCGTGTGGAACATTACCACAACTTCCAAAATTAACAATGAAAGGAAATGT
23041 ATCATATGGTTTCCAACCCACTAATGGTGTTGGTTACCAACCATACAGAGTAGTAGTACT
23041          23050     23060     23070     23080     23090
23041 TAGTATACCAAAGGTTGGGTGATTACCACAACCAATGGTTGGTATGTCTCATCATCATGA
23101 TTCTTTTGAACTTCTACATGCACCAGCAACTGTTTGTGGACCTAAAAAGTCTACTAATTT
23101          23110     23120     23130     23140     23150
23101 AAGAAAACTTGAAGATGTACGTGGTCGTTGACAAACACCTGGATTTTTCAGATGATTAAA
23161 GGTTAAAAACAAATGTGTCAATTTCAACTTCAATGGTTTAACAGGCACAGGTGTTCTTAC
23161          23170     23180     23190     23200     23210
23161 CCAATTTTTGTTTACACAGTTAAAGTTGAAGTTACCAAATTGTCCGTGTCCACAAGAATG
23221 TGAGTCTAACAAAAAGTTTCTGCCTTTCCAACAATTTGGCAGAGACATTGCTGACACTAC
23221          23230     23240     23250     23260     23270
23221 ACTCAGATTGTTTTTCAAAGACGGAAAGGTTGTTAAACCGTCTCTGTAACGACTGTGATG
                          >>>57_F>>> 23297 to 23318
23281 TGATGCTGTCCGTGATCCACAGACACTTGAGATTCTTGACATTACACCATGTTCTTTTGG
23281          23290     23300     23310     23320     23330
23281 ACTACGACAGGCACTAGGTGTCTGTGAACTCTAAGAACTGTAATGTGGTACAAGAAAACC
23341 TGGTGTCAGTGTTATAACACCAGGAACAAATACTTCTAACCAGGTTGCTGTTCTTTATCA
23341          23350     23360     23370     23380     23390
23341 ACCACAGTCACAATATTGTGGTCCTTGTTTATGAAGATTGGTCCAACGACAAGAAATAGT
```

<<<58_R<<< 23457 to 23478

```
23401 GGATGTTAACTGCACAGAAGTCCCTGTTGCTATTCATGCAGATCAACTTACTCCTACTTG
23401           23410     23420     23430     23440     23450
23401 CCTACAATTGACGTGTCTTCAGGGACAACGATAAGTACGTCTAGTTGAATGAGGATGAAC
23461 GCGTGTTTATTCTACAGGTTCTAATGTTTTTCAAACACGTGCAGGCTGTTTAATAGGGGC
23461           23470     23480     23490     23500     23510
23461 CGCACAAATAAGATGTCCAAGATTACAAAAAGTTTGTGCACGTCCGACAAATTATCCCCG
23521 TGAACATGTCAACAACTCATATGAGTGTGACATACCCATTGGTGCAGGTATATGCGCTAG
23521           23530     23540     23550     23560     23570
23521 ACTTGTACAGTTGTTGAGTATACTCACACTGTATGGGTAACCACGTCCATATACGCGATC
23581 TTATCAGACTCAGACTAATTCTCCTCGGCGGGCACGTAGTGTAGCTAGTCAATCCATCAT
23581           23590     23600     23610     23620     23630
23581 AATAGTCTGAGTCTGATTAAGAGGAGCCGCCCGTGCATCACATCGATCAGTTAGGTAGTA
23641 TGCCTACACTATGTCACTTGGTGCAGAAAATTCAGTTGCTTACTCTAATAACTCTATTGC
23641           23650     23660     23670     23680     23690
23641 ACGGATGTGATACAGTGAACCACGTCTTTTAAGTCAACGAATGAGATTATTGAGATAACG
23701 CATACCCACAAATTTTACTATTAGTGTTACCACAGAAATTCTACCAGTGTCTATGACCAA
23701           23710     23720     23730     23740     23750
23701 GTATGGGTGTTTAAAATGATAATCACAATGGTGTCTTTAAGATGGTCACAGATACTGGTT
23761 GACATCAGTAGATTGTACAATGTACATTTGTGGTGATTCAACTGAATGCAGCAATCTTTT
23761           23770     23780     23790     23800     23810
23761 CTGTAGTCATCTAACATGTTACATGTAAACACCACTAAGTTGACTTACGTCGTTAGAAAA
23821 GTTGCAATATGGCAGTTTTTGTACACAATTAAACCGTGCTTTAACTGGAATAGCTGTTGA
23821           23830     23840     23850     23860     23870
23821 CAACGTTATACCGTCAAAAACATGTGTTAATTTGGCACGAAATTGACCTTATCGACAACT
23881 ACAAGACAAAAACACCCAAGAAGTTTTTGCACAAGTCAAACAAATTTACAAAACACCACC
23881           23890     23900     23910     23920     23930
23881 TGTTCTGTTTTTGTGGGTTCTTCAAAAACGTGTTCAGTTTGTTTAAATGTTTTGTGGTGG
23941 AATTAAAGATTTTGGTGGTTTTAATTTTTCACAAATATTACCAGATCCATCAAAACCAAG
23941           23950     23960     23970     23980     23990
23941 TTAATTTCTAAAACCACCAAAATTAAAAAGTGTTTATAATGGTCTAGGTAGTTTTGGTTC
24001 CAAGAGGTCATTTATTGAAGATCTACTTTTCAACAAAGTGACACTTGCAGATGCTGGCTT
24001           24010     24020     24030     24040     24050
24001 GTTCTCCAGTAAATAACTTCTAGATGAAAAGTTGTTTCACTGTGAACGTCTACGACCGAA
                    >>>59_F>>> 24077 to 24098
24061 CATCAAACAATATGGTGATTGCCTTGGTGATATTGCTGCTAGAGACCTCATTTGTGCACA
24061           24070     24080     24090     24100     24110
24061 GTAGTTTGTTATACCACTAACGGAACCACTATAACGACGATCTCTGGAGTAAACACGTGT
24121 AAAGTTTAACGGCCTTACTGTTTTGCCACCTTTGCTCACAGATGAAATGATTGCTCAATA
24121           24130     24140     24150     24160     24170
24121 TTTCAAATTGCCGGAATGACAAAACGGTGGAAACGAGTGTCTACTTTACTAACGAGTTAT
                                                 <<<60_R<<< 24233 to 24254
24181 CACTTCTGCACTGTTAGCGGGTACAATCACTTCTGGTTGGACCTTTGGTGCAGGTGCTGC
24181           24190     24200     24210     24220     24230
24181 GTGAAGACGTGACAATCGCCCATGTTAGTGAAGACCAACCTGGAAACCACGTCCACGACG
24241 ATTACAAATACCATTTGCTATGCAAATGGCTTATAGGTTTAATGGTATTGGAGTTACACA
24241           24250     24260     24270     24280     24290
24241 TAATGTTTATGGTAAACGATACGTTTACCGAATATCCAAATTACCATAACCTCAATGTGT
24301 GAATGTTCTCTATGAGAACCAAAAATTGATTGCCAACCAATTTAATAGTGCTATTGGCAA
24301           24310     24320     24330     24340     24350
24301 CTTACAAGAGATACTCTTGGTTTTTAACTAACGGTTGGTTAAATTATCACGATAACCGTT
24361 AATTCAAGACTCACTTTCTTCCACAGCAAGTGCACTTGGAAAACTTCAAGATGTGGTCAA
24361           24370     24380     24390     24400     24410
24361 TTAAGTTCTGAGTGAAAGAAGGTGTCGTTCACGTGAACCTTTTGAAGTTCTACACCAGTT
24421 CCAAAATGCACAAGCTTTAAACACGCTTGTTAAACAACTTAGCTCCAATTTTGGTGCAAT
24421           24430     24440     24450     24460     24470
24421 GGTTTTACGTGTTCGAAATTTGTGCGAACAATTTGTTGAATCGAGGTTAAAACCACGTTA
```

```
24481 TTCAAGTGTTTTAAATGATATCCTTTCACGTCTTGACAAAGTTGAGGCTGAAGTGCAAAT
24481           24490     24500     24510     24520     24530
24481 AAGTTCACAAAATTTACTATAGGAAAGTGCAGAACTGTTTCAACTCCGACTTCACGTTTA
24541 TGATAGGTTGATCACAGGCAGACTTCAAAGTTTGCAGACATATGTGACTCAACAATTAAT
24541           24550     24560     24570     24580     24590
24541 ACTATCCAACTAGTGTCCGTCTGAAGTTTCAAACGTCTGTATACACTGAGTTGTTAATTA
24601 TAGAGCTGCAGAAATCAGAGCTTCTGCTAATCTTGCTGCTACTAAAATGTCAGAGTGTGT
24601           24610     24620     24630     24640     24650
24601 ATCTCGACGTCTTTAGTCTCGAAGACGATTAGAACGACGATGATTTTACAGTCTCACACA
24661 ACTTGGACAATCAAAAAGAGTTGATTTTTGTGGAAAGGGCTATCATCTTATGTCCTTCCC
24661           24670     24680     24690     24700     24710
24661 TGAACCTGTTAGTTTTTCTCAACTAAAAACACCTTTCCCGATAGTAGAATACAGGAAGGG
24721 TCAGTCAGCACCTCATGGTGTAGTCTTCTTGCATGTGACTTATGTCCCTGCACAAGAAAA
24721           24730     24740     24750     24760     24770
24721 AGTCAGTCGTGGAGTACCACATCAGAAGAACGTACACTGAATACAGGGACGTGTTCTTTT
24781 GAACTTCACAACTGCTCCTGCCATTTGTCATGATGGAAAAGCACACTTTCCTCGTGAAGG
24781           24790     24800     24810     24820     24830
24781 CTTGAAGTGTTGACGAGGACGGTAAACAGTACTACCTTTTCGTGTGAAAGGAGCACTTCC
                                                        >>>61_F>>> 24895 to
24919
24841 TGTCTTTGTTTCAAATGGCACACACTGGTTTGTAACACAAAGGAATTTTTATGAACCACA
24841           24850     24860     24870     24880     24890
24841 ACAGAAACAAAGTTTACCGTGTGTGACCAAACATTGTGTTTCCTTAAAAATACTTGGTGT
24901 AATCATTACTACAGACAACACATTTGTGTCTGGTAACTGTGATGTTGTAATAGGAATTGT
24901           24910     24920     24930     24940     24950
24901 TTAGTAATGATGTCTGTTGTGTAAACACAGACCATTGACACTACAACATTATCCTTAACA
24961 CAACAACACAGTTTATGATCCTTTGCAACCTGAATTAGACTCATTCAAGGAGGAGTTAGA
24961           24970     24980     24990     25000     25010
24961 GTTGTTGTGTCAAATACTAGGAAACGTTGGACTTAATCTGAGTAAGTTCCTCCTCAATCT
                                          <<<62_R<<< 25055 to 25076
25021 TAAATATTTTAAGAATCATACATCACCAGATGTTGATTTAGGTGACATCTCTGGCATTAA
25021           25030     25040     25050     25060     25070
25021 ATTTATAAAATTCTTAGTATGTAGTGGTCTACAACTAAATCCACTGTAGAGACCGTAATT
25081 TGCTTCAGTTGTAAACATTCAAAAAGAAATTGACCGCCTCAATGAGGTTGCCAAGAATTT
25081           25090     25100     25110     25120     25130
25081 ACGAAGTCAACATTTGTAAGTTTTTCTTTAACTGGCGGAGTTACTCCAACGGTTCTTAAA
25141 AAATGAATCTCTCATCGATCTCCAAGAACTTGGAAAGTATGAGCAGTATATAAAATGGCC
25141           25150     25160     25170     25180     25190
25141 TTTACTTAGAGAGTAGCTAGAGGTTCTTGAACCTTTCATACTCGTCATATATTTTACCGG
25201 ATGGTACATTTGGCTAGGTTTTATAGCTGGCTTGATTGCCATAGTAATGGTGACAATTAT
25201           25210     25220     25230     25240     25250
25201 TACCATGTAAACCGATCCAAAATATCGACCGAACTAACGGTATCATTACCACTGTTAATA
25261 GCTTTGCTGTATGACCAGTTGCTGTAGTTGTCTCAAGGGCTGTTGTTCTTGTGGATCCTG
25261           25270     25280     25290     25300     25310
25261 CGAAACGACATACTGGTCAACGACATCAACAGAGTTCCCGACAACAAGAACACCTAGGAC
25321 CTGCAAATTTGATGAAGACGACTCTGAGCCAGTGCTCAAAGGAGTCAAATTACATTACAC
25321           25330     25340     25350     25360     25370
25321 GACGTTTAAACTACTTCTGCTGAGACTCGGTCACGAGTTTCCTCAGTTTAATGTAATGTG
25381 ATAAACGAACTTATGGATTTGTTTATGAGAATCTTCACAATTGGAACTGTAACTTTGAAG
25381           25390     25400     25410     25420     25430
25381 TATTTGCTTGAATACCTAAACAAATACTCTTAGAAGTGTTAACCTTGACATTGAAACTTC
25441 CAAGGTGAAATCAAGGATGCTACTCCTTCAGATTTTGTTCGCGCTACTGCAACGATACCG
25441           25450     25460     25470     25480     25490
25441 GTTCCACTTTAGTTCCTACGATGAGGAAGTCTAAAACAAGCGCGATGACGTTGCTATGGC
25501 ATACAAGCCTCACTCCCTTTCGGATGGCTTATTGTTGGCGTTGCACTTCTTGCTGTTTTT
25501           25510     25520     25530     25540     25550
25501 TATGTTCGGAGTGAGGGAAAGCCTACCGAATAACAACCGCAACGTGAAGAACGACAAAAA
25561 CAGAGCGCTTCCAAAATCATAACCCTCAAAAAGAGATGGCAACTAGCACTCTCCAAGGGT
25561           25570     25580     25590     25600     25610
```

```
25561 GTCTCGCGAAGGTTTTAGTATTGGGAGTTTTTCTCTACCGTTGATCGTGAGAGGTTCCCA
                        >>>63_F>>> 25637 to 25658
25621 GTTCACTTTGTTTGCAACTTGCTGTTGTTGTTTGTAACAGTTTACTCACACCTTTTGCTC
25621          25630      25640      25650      25660      25670
25621 CAAGTGAAACAAACGTTGAACGACAACAACAAACATTGTCAAATGAGTGTGGAAAACGAG
25681 GTTGCTGCTGGCCTTGAAGCCCCTTTTCTCTATCTTTATGCTTTAGTCTACTTCTTGCAG
25681          25690      25700      25710      25720      25730
25681 CAACGACGACCGGAACTTCGGGGAAAAGAGATAGAAATACGAAATCAGATGAAGAACGTC
25741 AGTATAAACTTTGTAAGAATAATAATGAGGCTTTGGCTTTGCTGGAAATGCCGTTCCAAA
25741          25750      25760      25770      25780      25790
25741 TCATATTTGAAACATTCTTATTATTACTCCGAAACCGAAACGACCTTTACGGCAAGGTTT
                                  <<<64_R<<< 25835 to 25857
25801 AACCCATTACTTTATGATGCCAACTATTTTCTTTGCTGGCATACTAATTGTTACGACTAT
25801          25810      25820      25830      25840      25850
25801 TTGGGTAATGAAATACTACGGTTGATAAAAGAAACGACCGTATGATTAACAATGCTGATA
25861 TGTATACCTTACAATAGTGTAACTTCTTCAATTGTCATTACTTCAGGTGATGGCACAACA
25861          25870      25880      25890      25900      25910
25861 ACATATGGAATGTTATCACATTGAAGAAGTTAACAGTAATGAAGTCCACTACCGTGTTGT
25921 AGTCCTATTTCTGAACATGACTACCAGATTGGTGGTTATACTGAAAAATGGGAATCTGGA
25921          25930      25940      25950      25960      25970
25921 TCAGGATAAAGACTTGTACTGATGGTCTAACCACCAATATGACTTTTTACCCTTAGACCT
25981 GTAAAAGACTGTGTTGTATTACACAGTTACTTCACTTCAGACTATTACCAGCTGTACTCA
25981          25990      26000      26010      26020      26030
25981 CATTTTCTGACACAACATAATGTGTCAATGAAGTGAAGTCTGATAATGGTCGACATGAGT
26041 ACTCAATTGAGTACAGACACTGGTGTTGAACATGTTACCTTCTTCATCTACAATAAAATT
26041          26050      26060      26070      26080      26090
26041 TGAGTTAACTCATGTCTGTGACCACAACTTGTACAATGGAAGAAGTAGATGTTATTTTAA
26101 GTTGATGAGCCTGAAGAACATGTCCAAATTCACACAATCGACGGTTCATCCGGAGTTGTT
26101          26110      26120      26130      26140      26150
26101 CAACTACTCGGACTTCTTGTACAGGTTTAAGTGTGTTAGCTGCCAAGTAGGCCTCAACAA
26161 AATCCAGTAATGGAACCAATTTATGATGAACCGACGACGACTACTAGCGTGCCTTTGTAA
26161          26170      26180      26190      26200      26210
26161 TTAGGTCATTACCTTGGTTAAATACTACTTGGCTGCTGCTGATGATCGCACGGAAACATT
26221 GCACAAGCTGATGAGTACGAACTTATGTACTCATTCGTTTCGGAAGAGACAGGTACGTTA
26221          26230      26240      26250      26260      26270
26221 CGTGTTCGACTACTCATGCTTGAATACATGAGTAAGCAAAGCCTTCTCTGTCCATGCAAT
26281 ATAGTTAATAGCGTACTTCTTTTTCTTGCTTTCGTGGTATTCTTGCTAGTTACACTAGCC
26281          26290      26300      26310      26320      26330
26281 TATCAATTATCGCATGAAGAAAAAGAACGAAAGCACCATAAGAACGATCAATGTGATCGG
26341 ATCCTTACTGCGCTTCGATTGTGTGCGTACTGCTGCAATATTGTTAACGTGAGTCTTGTA
26341          26350      26360      26370      26380      26390
26341 TAGGAATGACGCGAAGCTAACACACGCATGACGACGTTATAACAATTGCACTCAGAACAT
                                          >>>65_F>>> 26443 to 26466
26401 AAACCTTCTTTTTACGTTTACTCTCGTGTTAAAAATCTGAATTCTTCTAGAGTTCCTGAT
26401          26410      26420      26430      26440      26450
26401 TTTGGAAGAAAAATGCAAATGAGAGCACAATTTTTAGACTTAAGAAGATCTCAAGGACTA
26461 CTTCTGGTCTAAACGAACTAAATATTATATTAGTTTTTCTGTTTGGAACTTTAATTTTAG
26461          26470      26480      26490      26500      26510
26461 GAAGACCAGATTTGCTTGATTTATAATATAATCAAAAAGACAAACCTTGAAATTAAAATC
                                                     <<<66_R<<< 26570 to 26591
26521 CCATGGCAGATTCCAACGGTACTATTACCGTTGAAGAGCTTAAAAAGCTCCTTGAACAAT
26521          26530      26540      26550      26560      26570
26521 GGTACCGTCTAAGGTTGCCATGATAATGGCAACTTCTCGAATTTTTCGAGGAACTTGTTA
26581 GGAACCTAGTAATAGGTTTCCTATTCCTTACATGGATTTGTCTTCTACAATTTGCCTATG
26581          26590      26600      26610      26620      26630
26581 CCTTGGATCATTATCCAAAGGATAAGGAATGTACCTAAACAGAAGATGTTAAACGGATAC
26641 CCAACAGGAATAGGTTTTTGTATATAATTAAGTTAATTTTCCTCTGGCTGTTATGGCCAG
26641          26650      26660      26670      26680      26690
26641 GGTTGTCCTTATCCAAAAACATATATTAATTCAATTAAAAGGAGACCGACAATACCGGTC
```

26701 TAACTTTAGCTTGTTTTGTGCTTGCTGCTGTTTACAGAATAAATTGGATCACCGGTGGAA
26701 26710 26720 26730 26740 26750
26701 ATTGAAATCGAACAAAACACGAACGACGACAAATGTCTTATTTAACCTAGTGGCCACCTT
26761 TTGCTATCGCAATGGCTTGTCTTGTAGGCTTGATGTGGCTCAGCTACTTCATTGCTTCTT
26761 26770 26780 26790 26800 26810
26761 AACGATAGCGTTACCGAACAGAACATCCGAACTACACCGAGTCGATGAAGTAACGAAGAA
26821 TCAGACTGTTTGCGCGTACGCGTTCCATGTGGTCATTCAATCCAGAAACTAACATTCTTC
26821 26830 26840 26850 26860 26870
26821 AGTCTGACAAACGCGCATGCGCAAGGTACACCAGTAAGTTAGGTCTTTGATTGTAAGAAG
26881 TCAACGTGCCACTCCATGGCACTATTCTGACCAGACCGCTTCTAGAAAGTGAACTCGTAA
26881 26890 26900 26910 26920 26930
26881 AGTTGCACGGTGAGGTACCGTGATAAGACTGGTCTGGCGAAGATCTTTCACTTGAGCATT
26941 TCGGAGCTGTGATCCTTCGTGGACATCTTCGTATTGCTGGACACCATCTAGGACGCTGTG
26941 26950 26960 26970 26980 26990
26941 AGCCTCGACACTAGGAAGCACCTGTAGAAGCATAACGACCTGTGGTAGATCCTGCGACAC
27001 ACATCAAGGACCTGCCTAAAGAAATCACTGTTGCTACATCACGAACGCTTTCTTATTACA
27001 27010 27020 27030 27040 27050
27001 TGTAGTTCCTGGACGGATTTCTTTAGTGACAACGATGTAGTGCTTGCGAAAGAATAATGT
27061 AATTGGGAGCTTCGCAGCGTGTAGCAGGTGACTCAGGTTTTGCTGCATACAGTCGCTACA
27061 27070 27080 27090 27100 27110
27061 TTAACCCTCGAAGCGTCGCACATCGTCCACTGAGTCCAAAACGACGTATGTCAGCGATGT
27121 GGATTGGCAACTATAAATTAAACACAGACCATTCCAGTAGCAGTGACAATATTGCTTTGC
27121 27130 27140 27150 27160 27170
27121 CCTAACCGTTGATATTTAATTTGTGTCTGGTAAGGTCATCGTCACTGTTATAACGAAACG
>>>67_F>>> 27204 to 27226
27181 TTGTACAGTAAGTGACAACAGATGTTTCATCTCGTTGACTTTCAGGTTACTATAGCAGAG
27181 27190 27200 27210 27220 27230
27181 AACATGTCATTCACTGTTGTCTACAAAGTAGAGCAACTGAAAGTCCAATGATATCGTCTC
27241 ATATTACTAATTATTATGAGGACTTTTAAAGTTTCCATTTGGAATCTTGATTACATCATA
27241 27250 27260 27270 27280 27290
27241 TATAATGATTAATAATACTCCTGAAAATTTCAAAGGTAAACCTTAGAACTAATGTAGTAT
27301 AACCTCATAATTAAAAATTTATCTAAGTCACTAACTGAGAATAAATATTCTCAATTAGAT
27301 27310 27320 27330 27340 27350
27301 TTGGAGTATTAATTTTTAAATAGATTCAGTGATTGACTCTTATTTATAAGAGTTAATCTA
<<<68_R<<< 27415 to
27436
27361 GAAGAGCAACCAATGGAGATTGATTAAACGAACATGAAAATTATTCTTTTCTTGGCACTG
27361 27370 27380 27390 27400 27410
27361 CTTCTCGTTGGTTACCTCTAACTAATTTGCTTGTACTTTTAATAAGAAAAGAACCGTGAC
27421 ATAACACTCGCTACTTGTGAGCTTTATCACTACCAAGAGTGTGTTAGAGGTACAACAGTA
27421 27430 27440 27450 27460 27470
27421 TATTGTGAGCGATGAACACTCGAAATAGTGATGGTTCTCACACAATCTCCATGTTGTCAT
27481 CTTTTAAAAGAACCTTGCTCTTCTGGAACATACGAGGGCAATTCACCATTTCATCCTCTA
27481 27490 27500 27510 27520 27530
27481 GAAAATTTTCTTGGAACGAGAAGACCTTGTATGCTCCCGTTAAGTGGTAAAGTAGGAGAT
27541 GCTGATAACAAATTTGCACTGACTTGCTTTAGCACTCAATTTGCTTTTGCTTGTCCTGAC
27541 27550 27560 27570 27580 27590
27541 CGACTATTGTTTAAACGTGACTGAACGAAATCGTGAGTTAAACGAAAACGAACAGGACTG
27601 GGCGTAAAACACGTCTATCAGTTACGTGCCAGATCAGTTTCACCTAAACTGTTCATCAGA
27601 27610 27620 27630 27640 27650
27601 CCGCATTTTGTGCAGATAGTCAATGCACGGTCTAGTCAAAGTGGATTTGACAAGTAGTCT
27661 CAAGAGGAAGTTCAAGAACTTTACTCTCCAATTTTTCTTATTGTTGCGGCAATAGTGTTT
27661 27670 27680 27690 27700 27710
27661 GTTCTCCTTCAAGTTCTTGAAATGAGAGGTTAAAAAGAATAACAACGCCGTTATCACAAA
27721 ATAACACTTTGCTTCACACTCAAAAGAAAGACAGAATGATTGAACTTTCATTAATTGACT
27721 27730 27740 27750 27760 27770
27721 TATTGTGAAACGAAGTGTGAGTTTTCTTTCTGTCTTACTAACTTGAAAGTAATTAACTGA
27781 TCTATTTGTGCTTTTTAGCCTTTCTGCTATTCCTTGTTTTAATTATGCTTATTATCTTTT
27781 27790 27800 27810 27820 27830

```
   27781 AGATAAACACGAAAAATCGGAAAGACGATAAGGAACAAAATTAATACGAATAATAGAAAA
   27841 GGTTCTCACTTGAACTGCAAGATCATAATGAAACTTGTCACGCCTAAACGAACATGAAAT
   27841           27850     27860     27870     27880     27890
   27841 CCAAGAGTGAACTTGACGTTCTAGTATTACTTTGAACAGTGCGGATTTGCTTGTACTTTA
   27901 TTCTTGTTTTCTTAGGAATCATCACAACTGTAGCTGCATTTCACCAAGAATGTAGTTTAC
   27901           27910     27920     27930     27940     27950
   27901 AAGAACAAAAGAATCCTTAGTAGTGTTGACATCGACGTAAAGTGGTTCTTACATCAAATG
   27961 AGTCATGTACTCAACATCAACCATATGTAGTTGATGACCCGTGTCCTATTCACTTCTATT
   27961           27970     27980     27990     28000     28010
   27961 TCAGTACATGAGTTGTAGTTGGTATACATCAACTACTGGGCACAGGATAAGTGAAGATAA
                                                        >>>69_F>>> 28072 to
28093
   28021 CTAAATGGTATATTAGAGTAGGAGCTAGAAAATCAGCACCTTTAATTGAATTGTGCGTGG
   28021           28030     28040     28050     28060     28070
   28021 GATTTACCATATAATCTCATCCTCGATCTTTTAGTCGTGGAAATTAACTTAACACGCACC
   28081 ATGAGGCTGGTTCTAAATCACCCATTCAGTACATCGATATCGGTAATTATACAGTTTCCT
   28081           28090     28100     28110     28120     28130
   28081 TACTCCGACCAAGATTTAGTGGGTAAGTCATGTAGCTATAGCCATTAATATGTCAAAGGA
   28141 GTTTACCTTTTACAATTAATTGCCAGGAACCTAAATTGGGTAGTCTTGTAGTGCGTTGTT
   28141           28150     28160     28170     28180     28190
   28141 CAAATGGAAAATGTTAATTAACGGTCCTTGGATTTAACCCATCAGAACATCACGCAACAA
                              <<<70_R<<< 28221 to 28242
   28201 CGTTCTATGAAGACTTTTTAGAGTATCATGACGTTCGTGTTGTTTTAGATTTCATCTAAA
   28201           28210     28220     28230     28240     28250
   28201 GCAAGATACTTCTGAAAAATCTCATAGTACTGCAAGCACAACAAAATCTAAAGTAGATTT
   28261 CGAACAAACTAAAATGTCTGATAATGGACCCCAAAATCAGCGAAATGCACCCCGCATTAC
   28261           28270     28280     28290     28300     28310
   28261 GCTTGTTTGATTTTACAGACTATTACCTGGGGTTTTAGTCGCTTTACGTGGGGCGTAATG
   28321 GTTTGGTGGACCCTCAGATTCAACTGGCAGTAACCAGAATGGAGAACGCAGTGGGGCGCG
   28321           28330     28340     28350     28360     28370
   28321 CAAACCACCTGGGAGTCTAAGTTGACCGTCATTGGTCTTACCTCTTGCGTCACCCCGCGC
   28381 ATCAAAACAACGTCGGCCCCAAGGTTTACCCAATAATACTGCGTCTTGGTTCACCGCTCT
   28381           28390     28400     28410     28420     28430
   28381 TAGTTTTGTTGCAGCCGGGGTTCCAAATGGGTTATTATGACGCAGAACCAAGTGGCGAGA
   28441 CACTCAACATGGCAAGGAAGACCTTAAATTCCCTCGAGGACAAGGCGTTCCAATTAACAC
   28441           28450     28460     28470     28480     28490
   28441 GTGAGTTGTACCGTTCCTTCTGGAATTTAAGGGAGCTCCTGTTCCGCAAGGTTAATTGTG
   28501 CAATAGCAGTCCAGATGACCAAATTGGCTACTACCGAAGAGCTACCAGACGAATTCGTGG
   28501           28510     28520     28530     28540     28550
   28501 GTTATCGTCAGGTCTACTGGTTTAACCGATGATGGCTTCTCGATGGTCTGCTTAAGCACC
   28561 TGGTGACGGTAAAATGAAAGATCTCAGTCCAAGATGGTATTTCTACTACCTAGGAACTGG
   28561           28570     28580     28590     28600     28610
   28561 ACCACTGCCATTTTACTTTCTAGAGTCAGGTTCTACCATAAAGATGATGGATCCTTGACC
   28621 GCCAGAAGCTGGACTTCCCTATGGTGCTAACAAAGACGGCATCATATGGGTTGCAACTGA
   28621           28630     28640     28650     28660     28670
   28621 CGGTCTTCGACCTGAAGGGATACCACGATTGTTTCTGCCGTAGTATACCCAACGTTGACT
   28681 GGGAGCCTTGAATACACCAAAAGATCACATTGGCACCCGCAATCCTGCTAACAATGCTGC
   28681           28690     28700     28710     28720     28730
   28681 CCCTCGGAACTTATGTGGTTTTCTAGTGTAACCGTGGGCGTTAGGACGATTGTTACGACG
   28741 AATCGTGCTACAACTTCCTCAAGGAACAACATTGCCAAAAGGCTTCTACGCAGAAGGGAG
   28741           28750     28760     28770     28780     28790
   28741 TTAGCACGATGTTGAAGGAGTTCCTTGTTGTAACGGTTTTCCGAAGATGCGTCTTCCCTC
                                                           >>>71_F>>> 28855 to
28876
   28801 CAGAGGCGGCAGTCAAGCCTCTTCTCGTTCCTCATCACGTAGTCGCAACAGTTCAAGAAA
   28801           28810     28820     28830     28840     28850
   28801 GTCTCCGCCGTCAGTTCGGAGAAGAGCAAGGAGTAGTGCATCAGCGTTGTCAAGTTCTTT
   28861 TTCAACTCCAGGCAGCAGTAGGGGAACTTCTCCTGCTAGAATGGCTGGCAATGGCGGTGA
   28861           28870     28880     28890     28900     28910
```

```
28861 AAGTTGAGGTCCGTCGTCATCCCCTTGAAGAGGACGATCTTACCGACCGTTACCGCCACT
28921 TGCTGCTCTTGCTTTGCTGCTGCTTGACAGATTGAACCAGCTTGAGAGCAAAATGTCTGG
28921          28930     28940     28950     28960     28970
28921 ACGACGAGAACGAAACGACGACGAACTGTCTAACTTGGTCGAACTCTCGTTTTACAGACC
                                   <<<72_R<<< 29009 to 29030
28981 TAAAGGCCAACAACAACAAGGCCAAACTGTCACTAAGAAATCTGCTGCTGAGGCTTCTAA
28981          28990     29000     29010     29020     29030
28981 ATTTCCGGTTGTTGTTGTTCCGGTTTGACAGTGATTCTTTAGACGACGACTCCGAAGATT
29041 GAAGCCTCGGCAAAAACGTACTGCCACTAAAGCATACAATGTAACACAAGCTTTCGGCAG
29041          29050     29060     29070     29080     29090
29041 CTTCGGAGCCGTTTTTGCATGACGGTGATTTCGTATGTTACATTGTGTTCGAAAGCCGTC
29101 ACGTGGTCCAGAACAAACCCAAGGAAATTTTGGGGACCAGGAACTAATCAGACAAGGAAC
29101          29110     29120     29130     29140     29150
29101 TGCACCAGGTCTTGTTTGGGTTCCTTTAAAACCCCTGGTCCTTGATTAGTCTGTTCCTTG
29161 TGATTACAAACATTGGCCGCAAATTGCACAATTTGCCCCCAGCGCTTCAGCGTTCTTCGG
29161          29170     29180     29190     29200     29210
29161 ACTAATGTTTGTAACCGGCGTTTAACGTGTTAAACGGGGGTCGCGAAGTCGCAAGAAGCC
                                                   >>>73_F>>> 29269 to 29290
29221 AATGTCGCGCATTGGCATGGAAGTCACACCTTCGGGAACGTGGTTGACCTACACAGGTGC
29221          29230     29240     29250     29260     29270
29221 TTACAGCGCGTAACCGTACCTTCAGTGTGGAAGCCCTTGCACCAACTGGATGTGTCCACG
29281 CATCAAATTGGATGACAAAGATCCAAATTTCAAAGATCAAGTCATTTTGCTGAATAAGCA
29281          29290     29300     29310     29320     29330
29281 GTAGTTTAACCTACTGTTTCTAGGTTTAAAGTTTCTAGTTCAGTAAAACGACTTATTCGT
29341 TATTGACGCATACAAAACATTCCCACCAACAGAGCCTAAAAAGGACAAAAAGAAGAAGGC
29341          29350     29360     29370     29380     29390
29341 ATAACTGCGTATGTTTTGTAAGGGTGGTTGTCTCGGATTTTTCCTGTTTTTCTTCTTCCG
29401 TGATGAAACTCAAGCCTTACCGCAGAGACAGAAGAAACAGCAAACTGTGACTCTTCTTCC
29401          29410     29420     29430     29440     29450
29401 ACTACTTTGAGTTCGGAATGGCGTCTCTGTCTTCTTTGTCGTTTGACACTGAGAAGAAGG
29461 TGCTGCAGATTTGGATGATTTCTCCAAACAATTGCAACAATCCATGAGCAGTGCTGACTC
29461          29470     29480     29490     29500     29510
29461 ACGACGTCTAAACCTACTAAAGAGGTTTGTTAACGTTGTTAGGTACTCGTCACGACTGAG
29521 AACTCAGGCCTAAACTCATGCAGACCACACAAGGCAGATGGGCTATATAAACGTTTTCGC
29521          29530     29540     29550     29560     29570
29521 TTGAGTCCGGATTTGAGTACGTCTGGTGTGTTCCGTCTACCCGATATATTTGCAAAAGCG
29581 TTTTCCGTTTACGATATATAGTCTACTCTTGTGCAGAATGAATTCTCGTAACTACATAGC
29581          29590     29600     29610     29620     29630
29581 AAAAGGCAAATGCTATATATCAGATGAGAACACGTCTTACTTAAGAGCATTGATGTATCG
29641 ACAAGTAGATGTAGTTAACTTTAATCTCACATAGCAATCTTTAATCAGTGTGTAACATTA
29641          29650     29660     29670     29680     29690
29641 TGTTCATCTACATCAATTGAAATTAGAGTGTATCGTTAGAAATTAGTCACACATTGTAAT
29701 GGGAGGACTTGAAAGAGCCACCACATTTTCACCGAGGCCACGCGGAGTACGATCGAGTGT
29701          29710     29720     29730     29740     29750
29701 CCCTCCTGAACTTTCTCGGTGGTGTAAAAGTGGCTCCGGTGCGCCTCATGCTAGCTCACA
                                        <<<74_R<<< 29794 to 29814
29761 ACAGTGAACAATGCTAGGGAGAGCTGCCTATATGGAAGAGCCCTAATGTGTAAAATTAAT
29761          29770     29780     29790     29800     29810
29761 TGTCACTTGTTACGATCCCTCTCGACGGATATACCTTCTCGGGATTACACATTTTAATTA
29821 TTTAGTAGTGCTATCCCCATGTGATTTTAATAGCTTCTTAGGAGAATGACAAAAAAAAAA
29821          29830     29840     29850     29860     29870
29821 AAATCATCACGATAGGGGTACACTAAAATTATCGAAGAATCCTCTTACTGTTTTTTTTTT
29881 AAAAAAAAAAAAAAAAAAAAAAA
29881          29890     29900
29881 TTTTTTTTTTTTTTTTTTTTTTT
```

```
Primer:    Sequence:
1_F   5'-ACCAACTTTCGATCTCTTGTAG-3'
2_R   5'-ACTTCTACTAAGCCACAAGTGC-3'
```

3_F 5'-TTCATGCACTTTGTCCGAAC-3'
4_R 5'-CGTGTACCAAGCAATTTCATG-3'
5_F 5'-TTTGTGGAAACTGTGAAAGG-3'
6_R 5'-CAAATGCATAAAGAGGACTCAG-3'
7_F 5'-TACAACCATTAGAACAACCTACT-3'
8_R 5'-TTGAGTGTGAAGGTATTGTTTG-3'
9_F 5'-TGTTAATGCAGCCAATGTTTAC-3'
10_R 5'-AAGTGGTCCATTAGTAGCTATG-3'
11_F 5'-AAATGGTTACACTGTAGAGGAG-3'
12_R 5'-ATTAATTTGCGTGTTTCTTCTGC-3'
13_F 5'-TTACCTAATGATGACACTCTACG-3'
14_R 5'-CCATTAACTTGTGGGTATTTCC-3'
15_F 5'-AATTGGATGGTGTTGTTTGTAC-3'
16_R 5'-CTCTTGAAGCAGGTTTCTTATAAC-3'
17_F 5'-ACCGTGTTTGTACTAATTATATGCC-3'
18_R 5'-ACCGACACTCTTAACAGTATTC-3'
19_F 5'-GAGCAACAAGAGTCGAATGTAC-3'
20_R 5'-AGTCTCTCGCAACTTCATCAC-3'
21_F 5'-TGGAACGTTAAAGATTTCATGTC-3'
22_R 5'-CTTGTCTAGTAGTTGCACATGTC-3'
23_F 5'-GATTCTGAGTACTGTAGGCACG-3'
24_R 5'-ATTTACAGCATCTACACCACAG-3'
25_F 5'-CCACAAACCTCTATCACCTCAG-3'
26_R 5'-CATCAAGCCAAAGACCGTTAAG-3'
27_F 5'-TTTCTGCTCAAACTGGAATTGC-3'
28_R 5'-GGAAAGTAACACCTGAGCATTG-3'
29_F 5'-TCAACCGCTACTTTAGACTGAC-3'
30_R 5'-TGTTGAGTTTGAAGGCATCTATG-3'
31_F 5'-AATACCTCTTACAACAGCAGCC-3'
32_R 5'-TAATTTGACAGCAGAATTGGCC-3'
33_F 5'-AACTTGTGCTAATGACCCTGTG-3'
34_R 5'-TAAGACGGGCTGCACTTACAC-3'
35_F 5'-TTGACACTGACTTAACAAAGCC-3'
36_R 5'-ATTTGGGTGGTATGTCTGATCC-3'
37_F 5'-ACGTAATGTCATCCCTACTATAACTC-3'
38_R 5'-TTTGCTTGTTCCAATTACTACAG-3'
39_F 5'-GTGTACCTTCCTTACCCAGATC-3'
40_R 5'-TGAAAGACATCAGCATACTCCTG-3'
41_F 5'-CTATGGTGATGCTGTTGTTTACC-3'
42_R 5'-TCTAACATAGTGCTCTTGTGGC-3'
43_F 5'-AATTCCTTACACGTAACCCTGC-3'
44_R 5'-ATTCTGAGCCCTGTGATGAATC-3'
45_F 5'-CCTTGGAATGTAGTGCGTATAAAG-3'
46_R 5'-TATCACATAGACAACAGGTGCG-3'
47_F 5'-CTTAACTTGCCTGGTTGTGATG-3'
48_R 5'-ACAGACAGCACCACCTAAATTG-3'
49_F 5'-CTGTAGGTCCCAAACAAGCTAG-3'
50_R 5'-CAATTTCCATTTGACTCCTGGG-3'
51_F 5'-TTAGCTGTACCCTATAATATGAGAG-3'
52_R 5'-TACAGTTGCACAATCACCAATC-3'
53_F 5'-TACAACCAGAACTCAATTACCC-3'
54_R 5'-AGCATGGAACCAAGTAACATTG-3'
55_F 5'-ACCCTCTCTCAGAAACAAAGTG-3'
56_R 5'-AGATGCAAATCTGGTGGCG-3'
57_F 5'-CCACAGACACTTGAGATTCTTG-3'
58_R 5'-CCTGTAGAATAAACACGCCAAG-3'
59_F 5'-GATTGCCTTGGTGATATTGCTG-3'
60_R 5'-ATGGTATTTGTAATGCAGCACC-3'
61_F 5'-ACCACAAATCATTACTACAGACAAC-3'

62_R 5'-TGCCAGAGATGTCACCTAAATC-3'
63_F 5'-ACTTGCTGTTGTTGTTTGTAAC-3'
64_R 5'-GTCGTAACAATTAGTATGCCAGC-3'
65_F 5'-TCTTCTAGAGTTCCTGATCTTCTG-3'
66_R 5'-TACTAGGTTCCATTGTTCAAGG-3'
67_F 5'-GTTTCATCTCGTTGACTTTCAGG-3'
68_R 5'-AAGTAGCGAGTGTTATCAGTGC-3'
69_F 5'-TGTGCSTGGATGAGGCTGGTTC-3'
70_R 5'-CAACACGAACGTCATGATACTC-3'
71_F 5'-AAGAAATTCAACTCCAGGCAGC-3'
72_R 5'-CAGCAGCAGATTTCTTAGTGAC-3'
73_F 5'-CTACACAGGTGCCATCAAATTG-3'
74_R 5'-TTTACACATTAGGGCTCTTCC-3'
